# Supplementary material for: Efficient Synthesis of 4,8-Dibromo Derivative of Strong Electron-Deficient Benzo[1,2-d:4,5-d’]bis([1,2,3]thiadiazole) and Its SNAr and Cross-Coupling Reactions
Source: Molecules. 2022 Oct 30;27(21):7372. doi: 10.3390/molecules27217372 (PMC9655153; doi:10.3390/molecules27217372)
Supplement: Supplementary file 1 [file molecules-27-07372-s001.zip › molecules-1999475-supplementary.pdf]

Supplementary Materials

Efficient synthesis of 4,8-dibromo derivative of strong electron-deficient benzo[1,2-d:4,5-d']bis([1,2,3]thiadiazole) and its SNAr and cross-coupling reactions

Timofey N. Chmovzh [1,2], Daria A. Alekhina[1], Timofey A. Kudryashev [1,3], and Oleg A. Rakitin [1,*]

[1] *N. D. Zelinsky Institute of Organic Chemistry, Russian Academy of Sciences, 119991 Moscow, Russian Federation*

[2] *Nanotechnology Education and Research Center, South Ural State University, 454080 Chelyabinsk, Russia*

[3] *Department of Chemistry, Moscow State University, 119899 Moscow, Russia*

* *Correspondence: orakitin@ioc.ac.ru; Tel.: +7 499 135 5327*

Table of contents:

^{1}H, ^{13}C NMR and HRMS spectra………………………………………………………… S2

Quantum-chemical calculations……………………………………………………..S56

^{1}H, ^{13}C NMR and HRMS spectra

4,8-Dibromobenzo[1,2-d:4,5-d']bis([1,2,3]thiadiazole) (1)

^{13}C NMR(75 MHz)

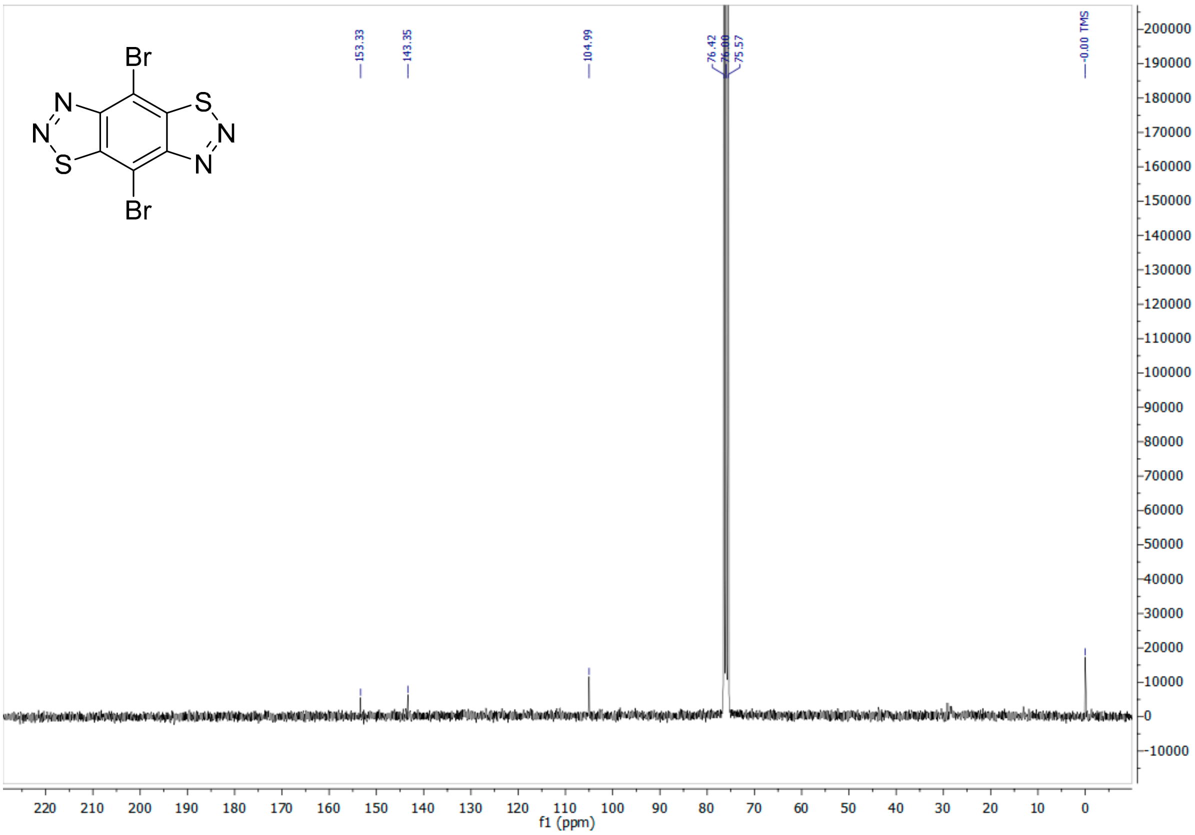

Display Report

Analysis Info

Analysis Name	D:\Data\Kolotyrkina\2021\Chmovsh\0831016.d	Acquisition Date	31.08.2021 12:58:20
Method	tune_50-1600.m	Operator	BDAL@DE
Sample Name	/SUSU Tim-491	Instrument / Ser#	micrOTOF 10248
Comment	C6Br2N4S2 mH 350.800 clb added, CH3OH		

Acquisition Parameter

Source Type	ESI	Ion Polarity	Positive	Set Nebulizer	1.0 Bar
Focus	Not active			Set Dry Heater	200 °C
Scan Begin	50 m/z	Set Capillary	4500 V	Set Dry Gas	4.0 l/min
Scan End	1600 m/z	Set End Plate Offset	-500 V	Set Divert Valve	Waste

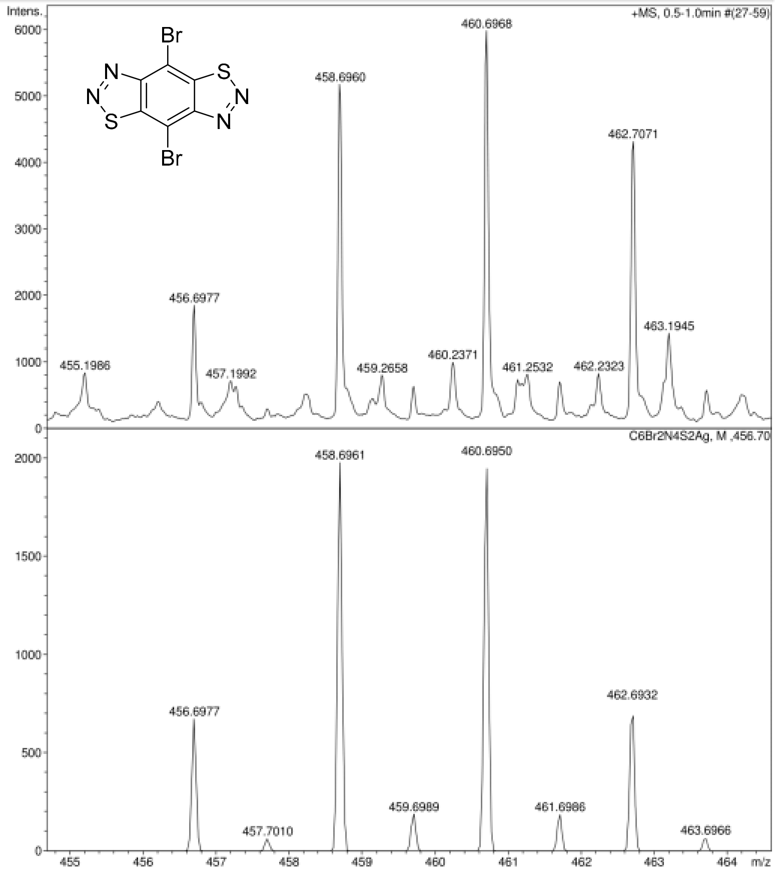

Bruker Compass DataAnalysis 4.0 printed: 31.08.2021 13:06:50 Page 1 of 1

4-(8-Bromobenzo[1,2-d:4,5-d']bis([1,2,3]thiadiazole)-4-yl)morpholine (12a)

^{1}H NMR (300 MHz)

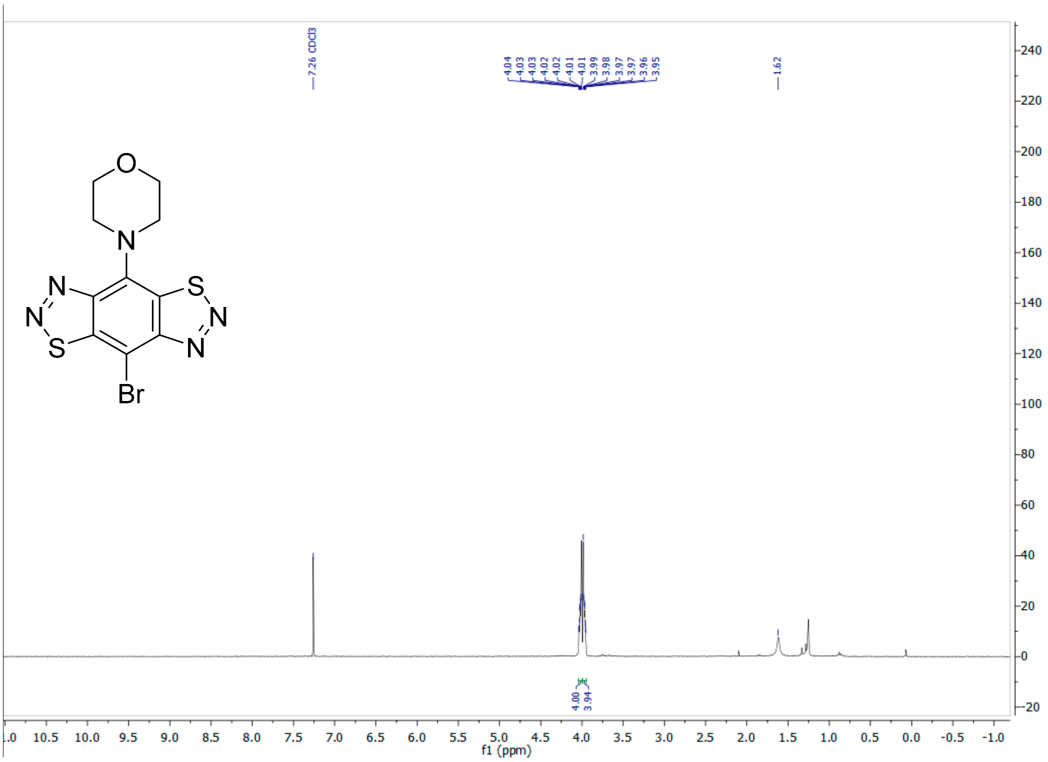

^{13}C NMR(75 MHz)

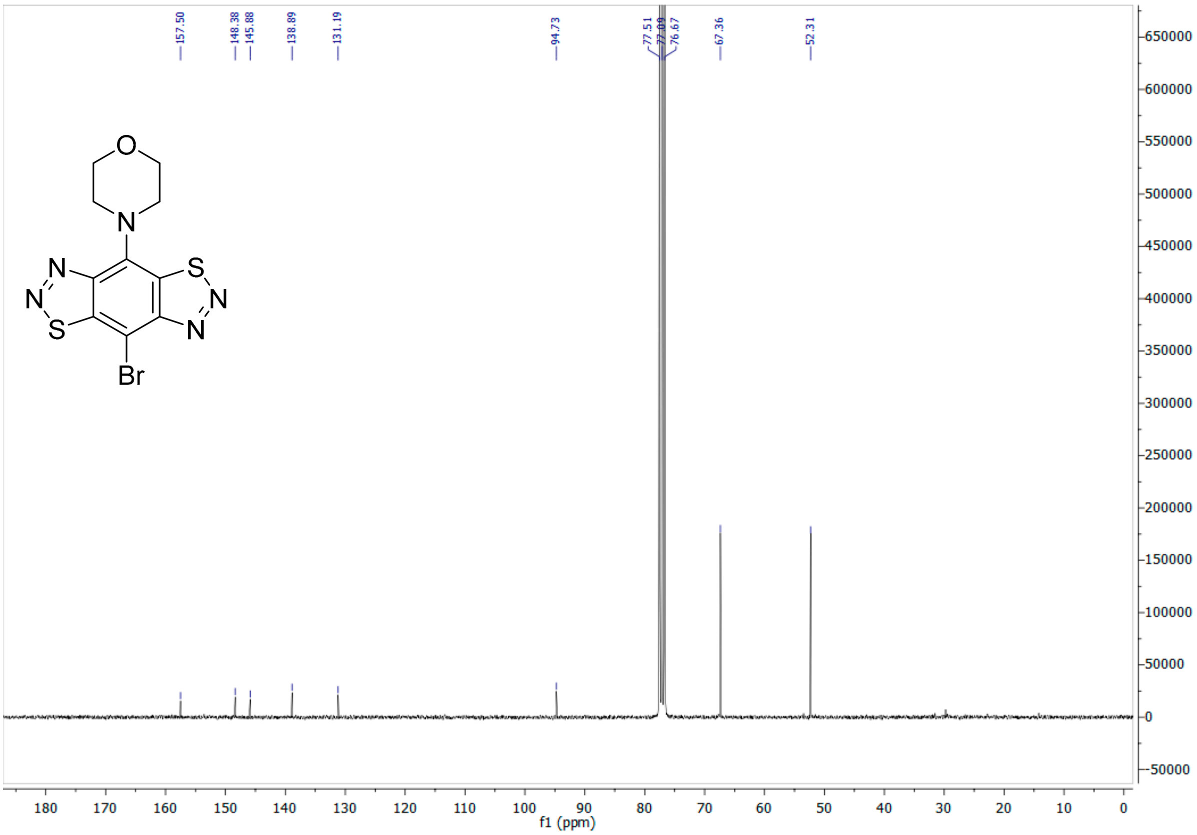
157.50
148.38
145.88
138.89
131.19
94.73
77.51
77.09
76.67
67.36
52.31
O
N
N
N
S
S
N
N
Br
650000
600000
550000
500000
450000
400000
350000
300000
250000
200000
150000
100000
50000
0
-50000
180 170 160 150 140 130 120 110 100 90 80 70 60 50 40 30 20 10 0
f1 (ppm)

Display Report

Analysis Info

Analysis Name	D:\Data\Kolotyrkina\2021\Chmovsh\0928014.d	Acquisition Date	28.09.2021 11:53:23
Method	tune_50-1600.m	Operator	BDAL@DE
Sample Name	/SUSU Tim-493	Instrument / Ser#	micrOTOF 10248
Comment	C10H8BrN5OS2 mH 357.9426 calibrant added CH3CN		

Acquisition Parameter

Source Type	ESI	Ion Polarity	Positive	Set Nebulizer	1.0 Bar
Focus	Not active			Set Dry Heater	200 °C
Scan Begin	50 m/z	Set Capillary	4500 V	Set Dry Gas	4.0 l/min
Scan End	1600 m/z	Set End Plate Offset	-500 V	Set Divert Valve	Waste

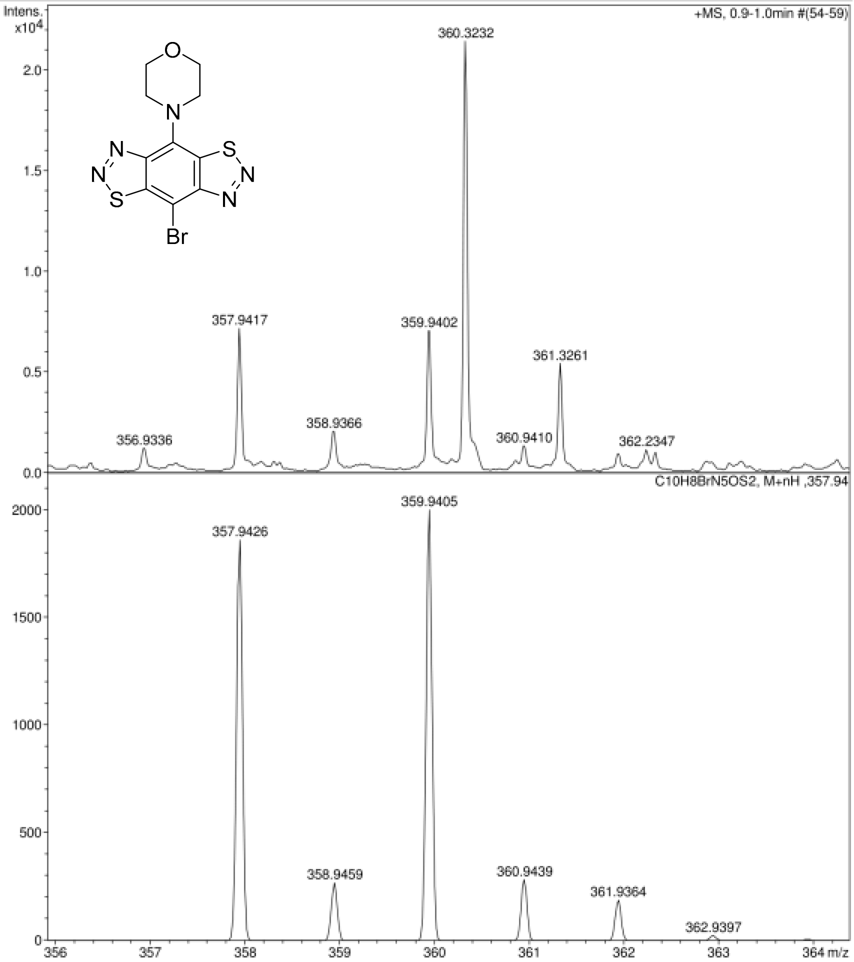

Bruker Compass DataAnalysis 4.0 printed: 28.09.2021 12:04:04 Page 1 of 1

4-Bromo-8-(piperidin-1-yl)benzo[1,2-d:4,5-d']bis([1,2,3]thiadiazole) (12b)

^{1}H NMR (300 MHz)

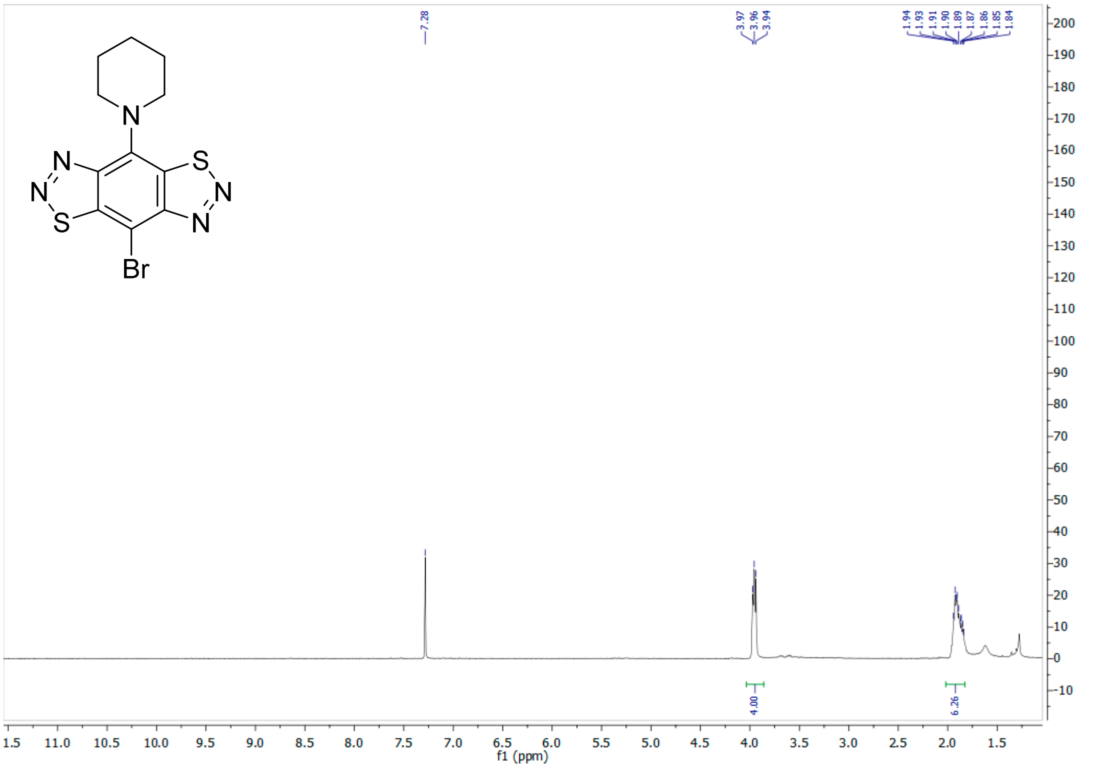

^{13}C NMR(75 MHz)

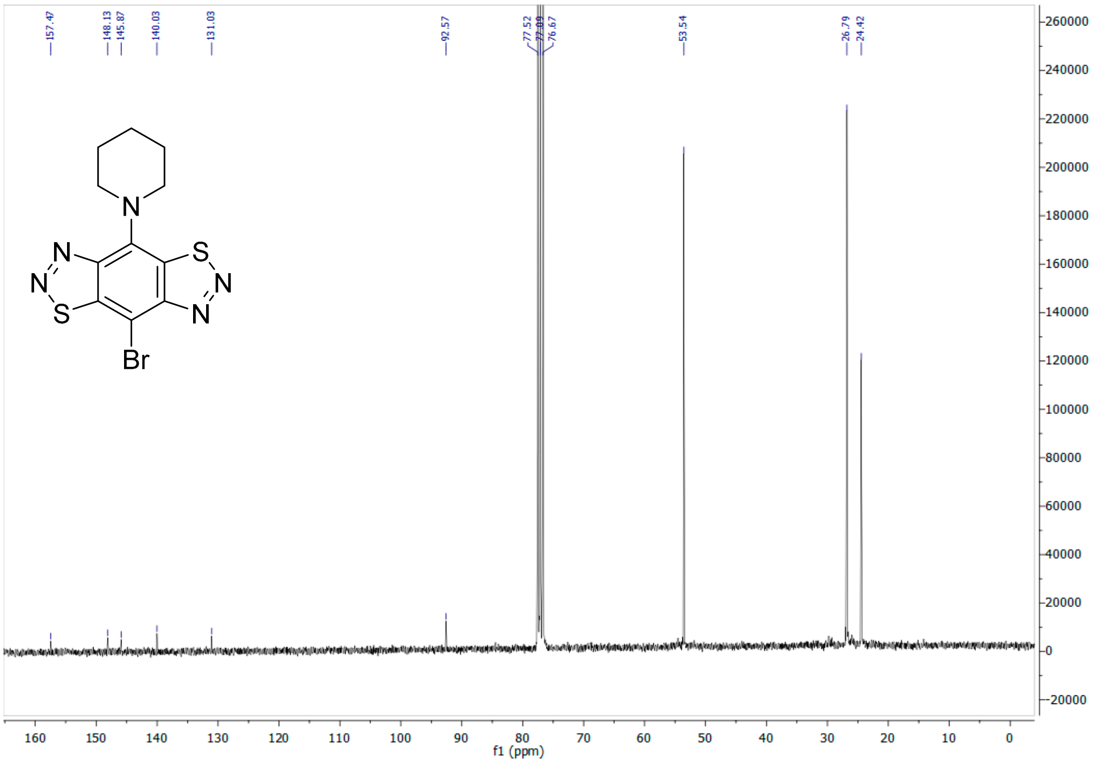

Display Report

Analysis Info

Analysis Name	D:\Data\Kolotyrkina\2022\Chmovsh\0601011.d	Acquisition Date	01.06.2022 12:44:42
Method	tune_50-1600_pos_15_12.m	Operator	BDAL@DE
Sample Name	/SUSU Tim-531	Instrument / Ser#	micrOTOF 10248
Comment	C11H10BrN5S2 mH 355.9644 calibrant added CH3CN		

Acquisition Parameter

Source Type	ESI	Ion Polarity	Positive	Set Nebulizer	0.4 Bar
Focus	Not active			Set Dry Heater	180 °C
Scan Begin	50 m/z	Set Capillary	4500 V	Set Dry Gas	4.0 l/min
Scan End	1600 m/z	Set End Plate Offset	-500 V	Set Divert Valve	Waste

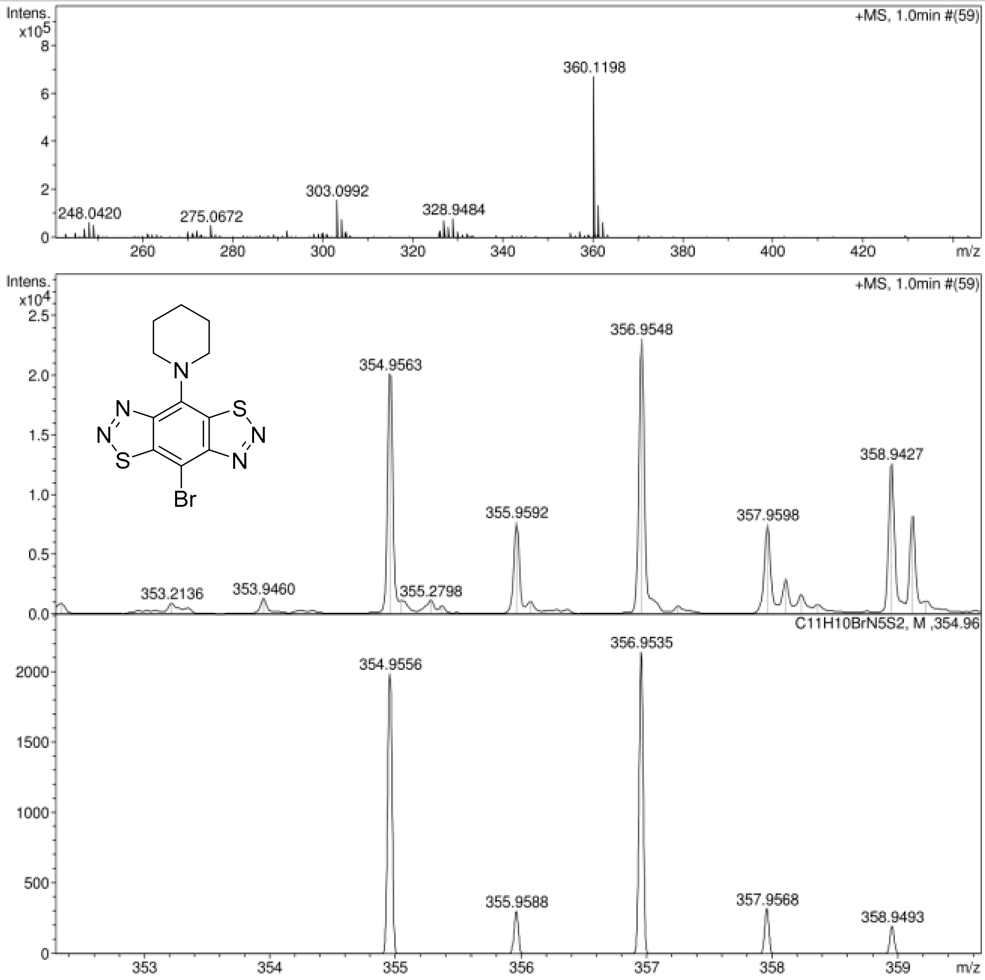

4-Bromo-8-(pyrrolidin-1-yl)benzo[1,2-d:4,5-d']bis([1,2,3]thiadiazole) (12c)

^{1}H NMR (300 MHz)

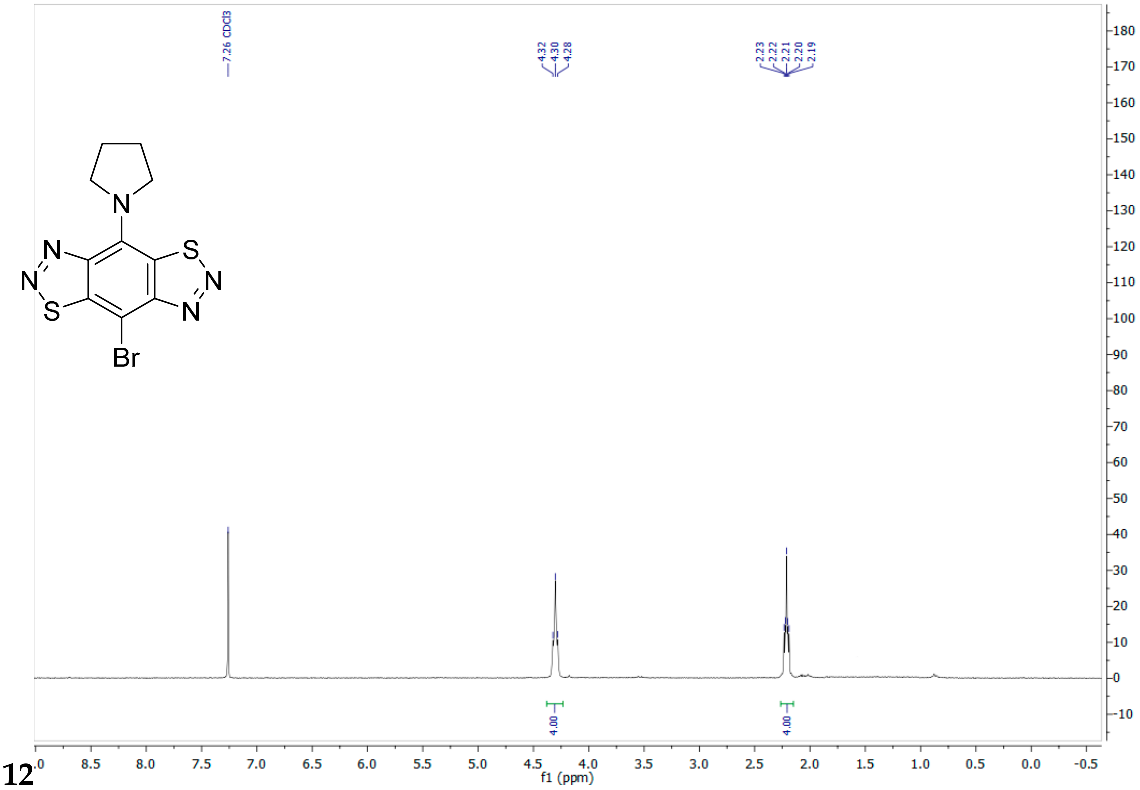

^{13}C NMR(75 MHz)

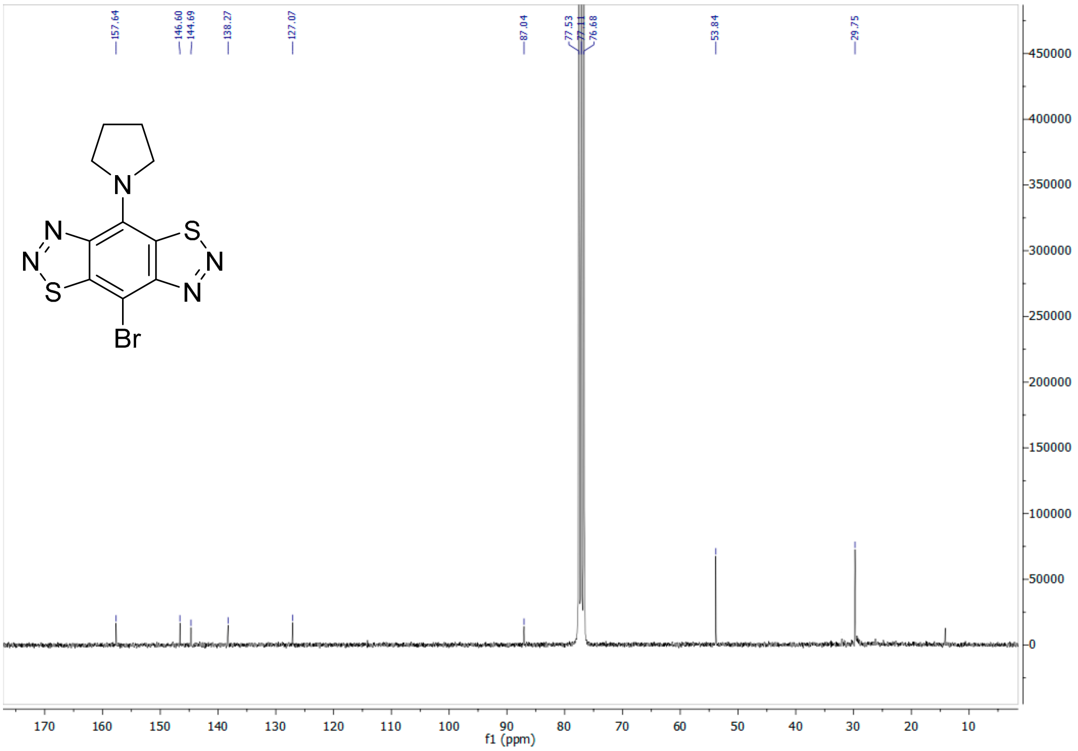

Display Report

Analysis Info

Analysis Name	D:\Data\Kolotyrkina\2021\Chmovsh\0928045.d	Acquisition Date	28.09.2021 17:11:46
Method	tune_50-1600.m	Operator	BDAL@DE
Sample Name	/SUSU Tim-496	Instrument / Ser#	micrOTOF 10248
Comment	C10H8BrN5S2 mH 341.9477 calibrant added CH3OH		

Acquisition Parameter

Source Type	ESI	Ion Polarity	Positive			Set Nebulizer	1.0 Bar
Focus	Not active					Set Dry Heater	200 °C
Scan Begin	50 m/z	Set Capillary	4500 V			Set Dry Gas	4.0 l/min
Scan End	1600 m/z	Set End Plate Offset	-500 V			Set Divert Valve	Waste

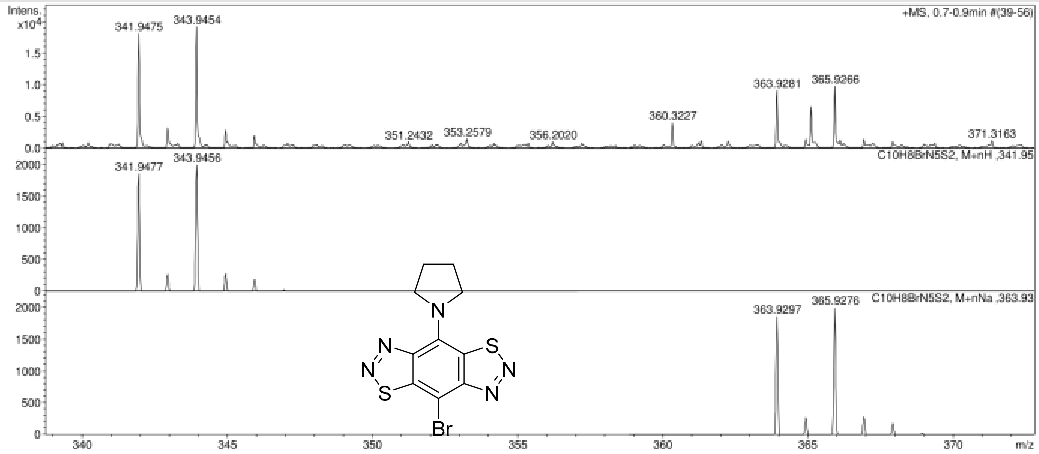

Bruker Compass DataAnalysis 4.0 printed: 28.09.2021 17:15:42 Page 1 of 1

4-Bromo-8-(2,3,3a,8b-tetrahydrocyclopenta[b]indol-4(1H)-yl)benzo[1,2-d:4,5-d']bis([1,2,3]thiadiazole) (12d)

^{1}H NMR (300 MHz)

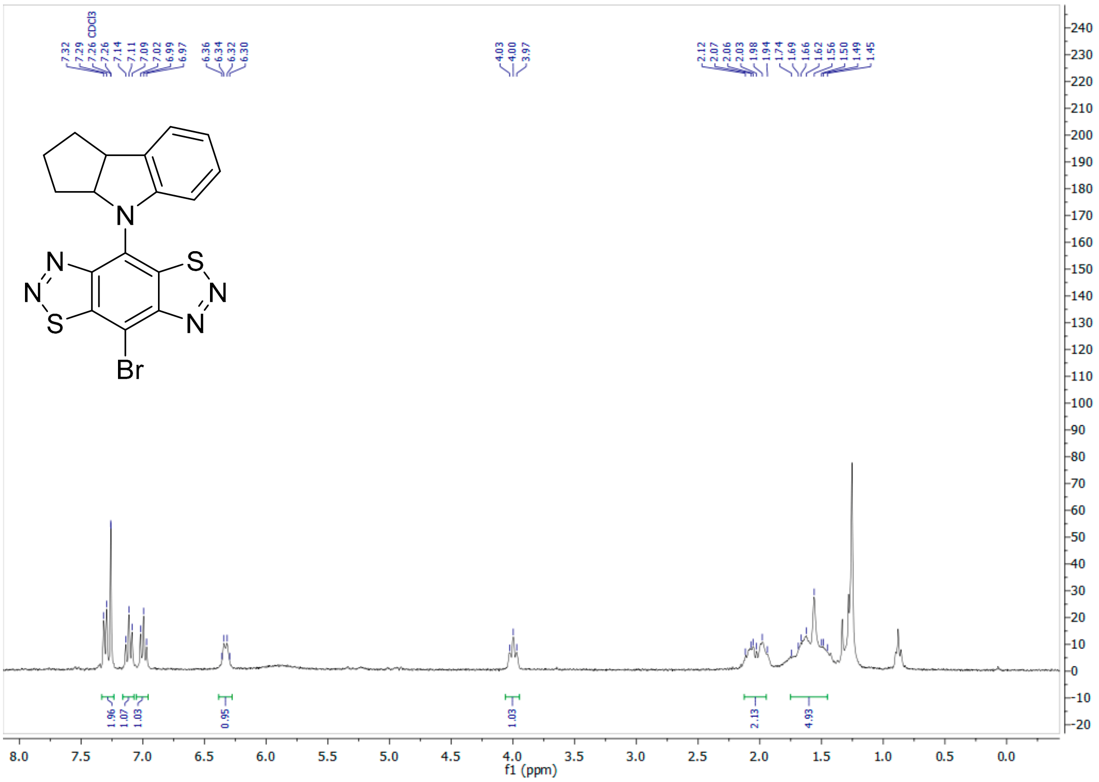

^{13}C NMR(75 MHz)

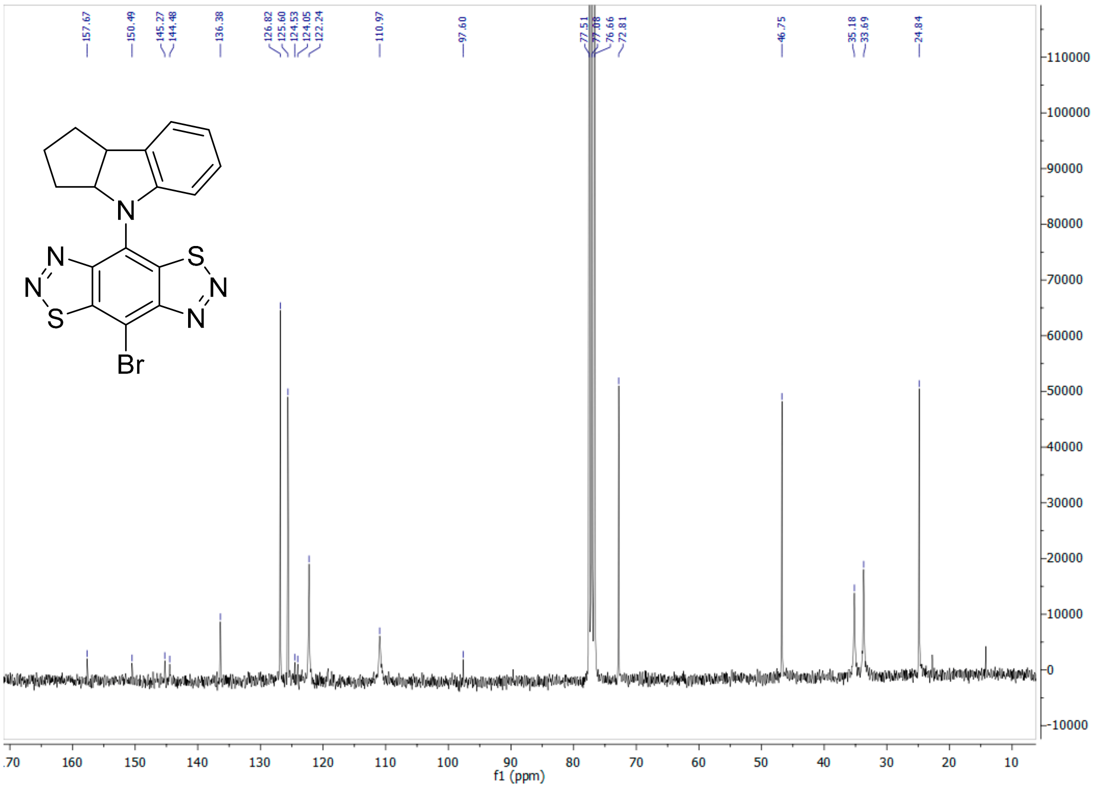
157.67
150.49
145.27
144.48
136.38
126.82
125.60
124.53
124.05
122.24
110.97
97.60
77.51
77.08
76.66
72.81
46.75
35.18
33.69
24.84
N
S
N
N
S
N
N
Br
110000
100000
90000
80000
70000
60000
50000
40000
30000
20000
10000
0
-10000
f1 (ppm)

Display Report

Analysis Info

Analysis Name	D:\Data\Chizhov\Rakitin\Chmovzh\tim-550_&clblow.d	Acquisition Date	02.09.2022 16:28:34
Method	tune_low.m	Operator	BDAL@DE
Sample Name	/SUSU Tim-550	Instrument / Ser#	micrOTOF 10248
Comment	CH3CN 100 %, dil. 200, calibrant added		

Acquisition Parameter

Source Type	ESI	Ion Polarity	Positive	Set Nebulizer	0.4 Bar
Focus	Not active			Set Dry Heater	180 °C
Scan Begin	50 m/z	Set Capillary	4500 V	Set Dry Gas	4.0 l/min
Scan End	3000 m/z	Set End Plate Offset	-500 V	Set Divert Valve	Waste

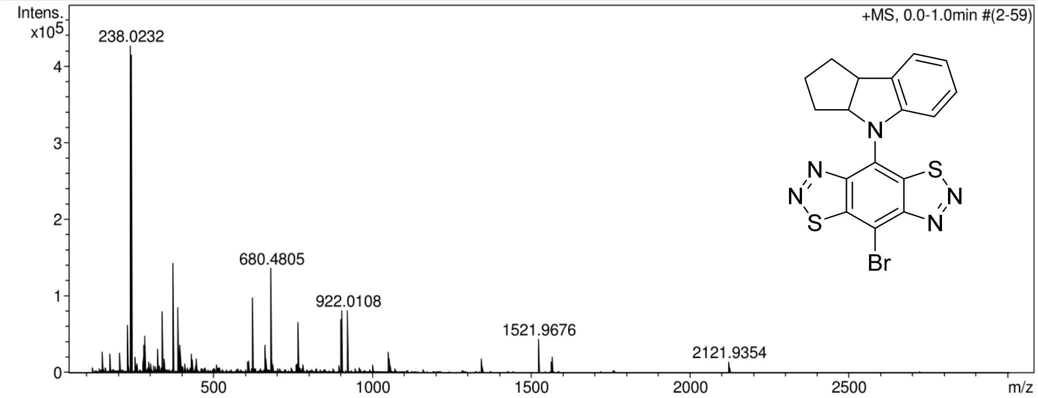

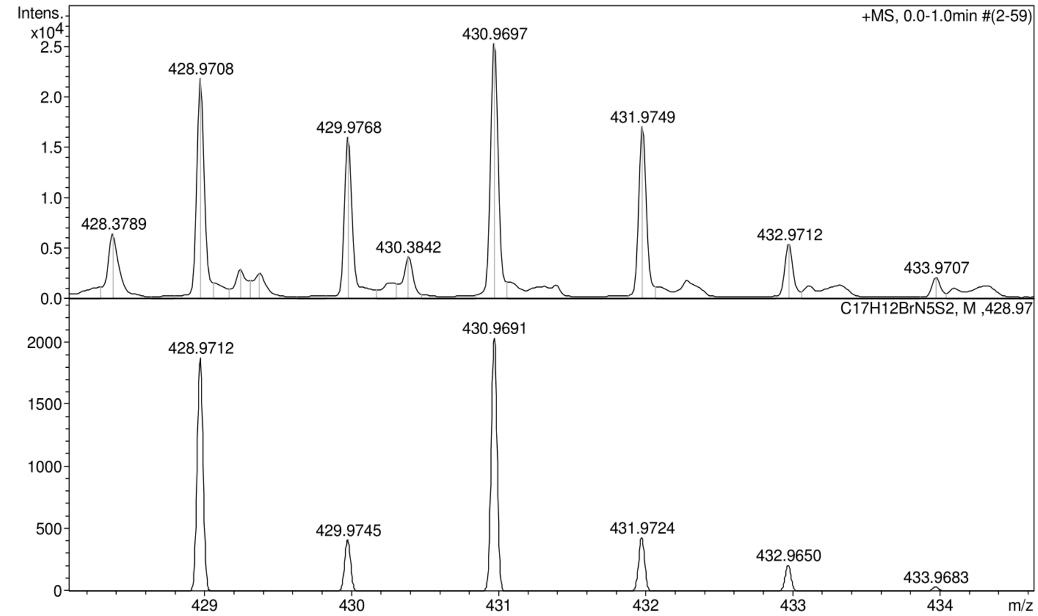

8-Bromo-N-phenylbenzo[1,2-d:4,5-d']bis([1,2,3]thiadiazole)-4-amine (12e)

^{1}H NMR (300 MHz)

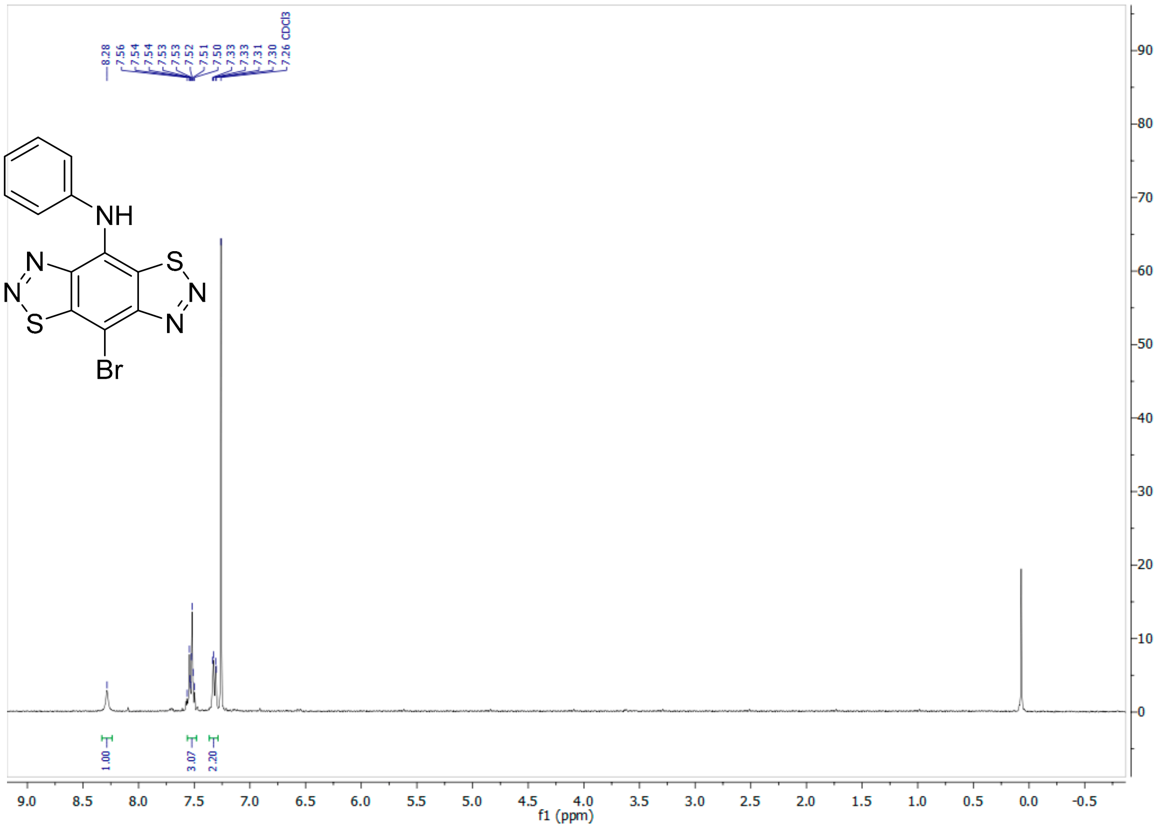

^{13}C NMR(75 MHz)

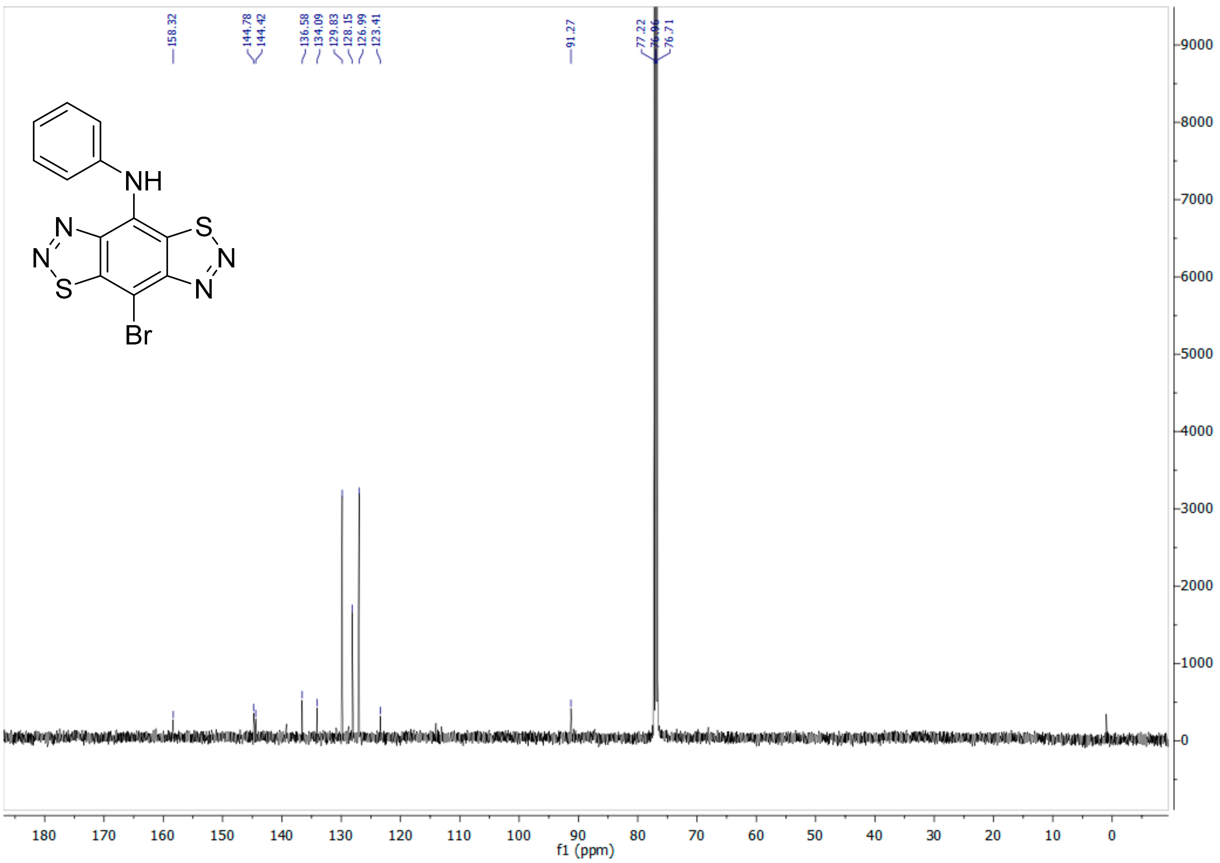
158.32
144.78
144.42
136.58
134.09
129.83
128.15
126.99
123.41
91.27
77.22
76.96
76.71
NH
N
N
S
S
N
N
Br
9000
8000
7000
6000
5000
4000
3000
2000
1000
0
180
170
160
150
140
130
120
110
100
90
80
70
60
50
40
30
20
10
0
f1 (ppm)

Display Report

Analysis Info

Analysis Name	D:\Data\Kolotyrkina\2022\Chmovsh\0608027.d	Acquisition Date	08.06.2022 16:38:37
Method	tune_50-1600_pos_15_12.m	Operator	BDAL@DE
Sample Name	/SUSU Tim-532	Instrument / Ser#	micrOTOF 10248
Comment	C12H6BrN5S2 mH 363.9320 clb added CH3CN		

Acquisition Parameter

Source Type	ESI	Ion Polarity	Positive	Set Nebulizer	0.4 Bar
Focus	Not active			Set Dry Heater	180 °C
Scan Begin	50 m/z	Set Capillary	4500 V	Set Dry Gas	4.0 l/min
Scan End	1600 m/z	Set End Plate Offset	-500 V	Set Divert Valve	Waste

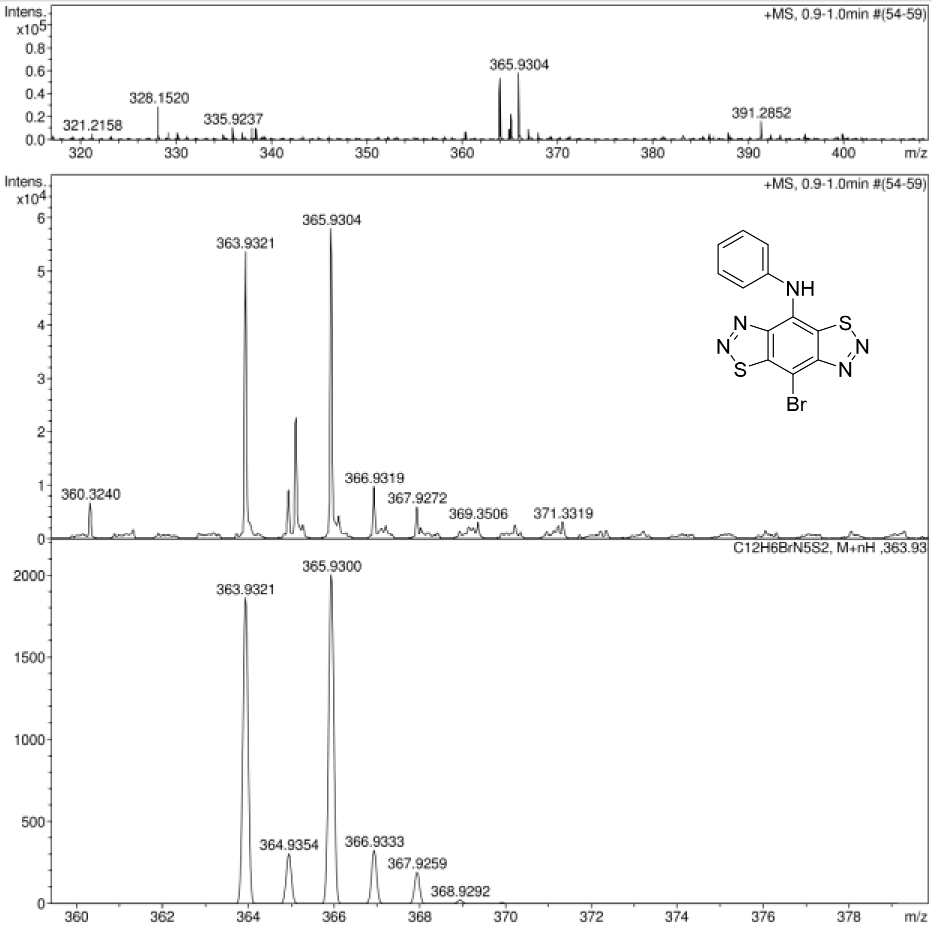

4,8-Dimorpholinobenzo[1,2-d:4,5-d']bis([1,2,3]thiadiazole) (13a)

^{1}H NMR (300 MHz)

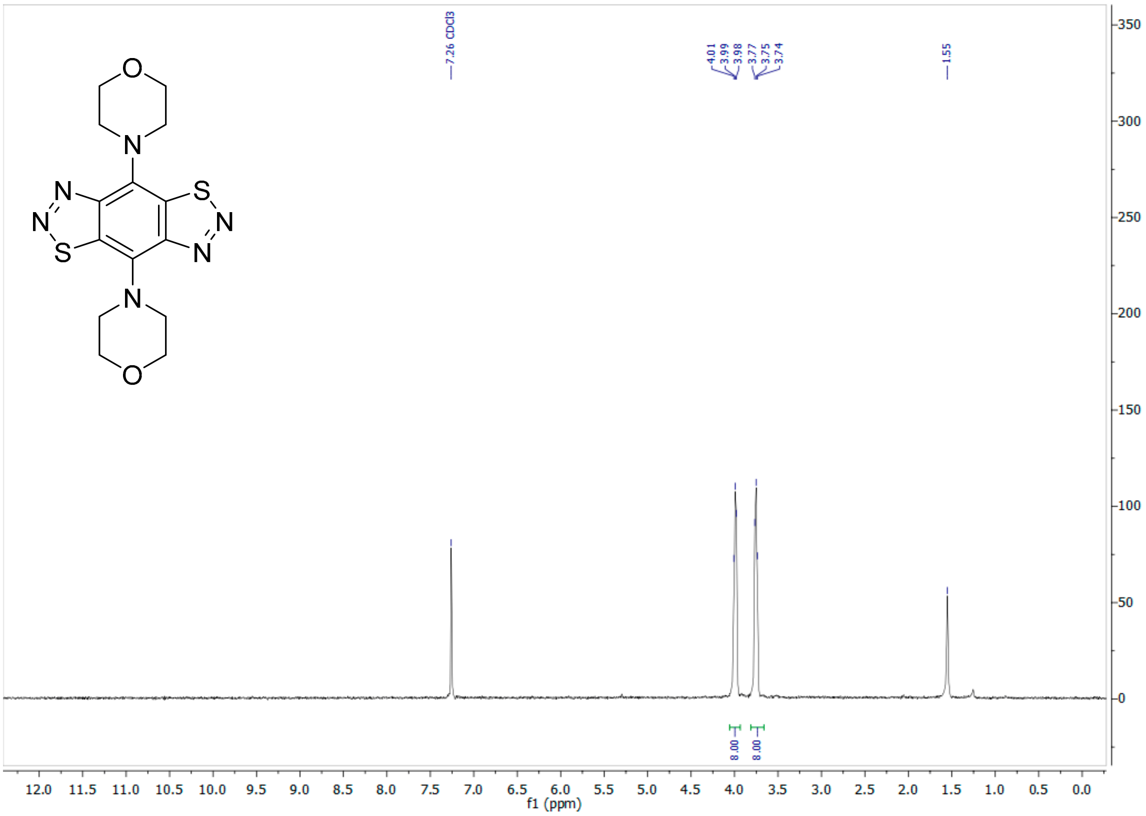

^{13}C NMR(75 MHz)

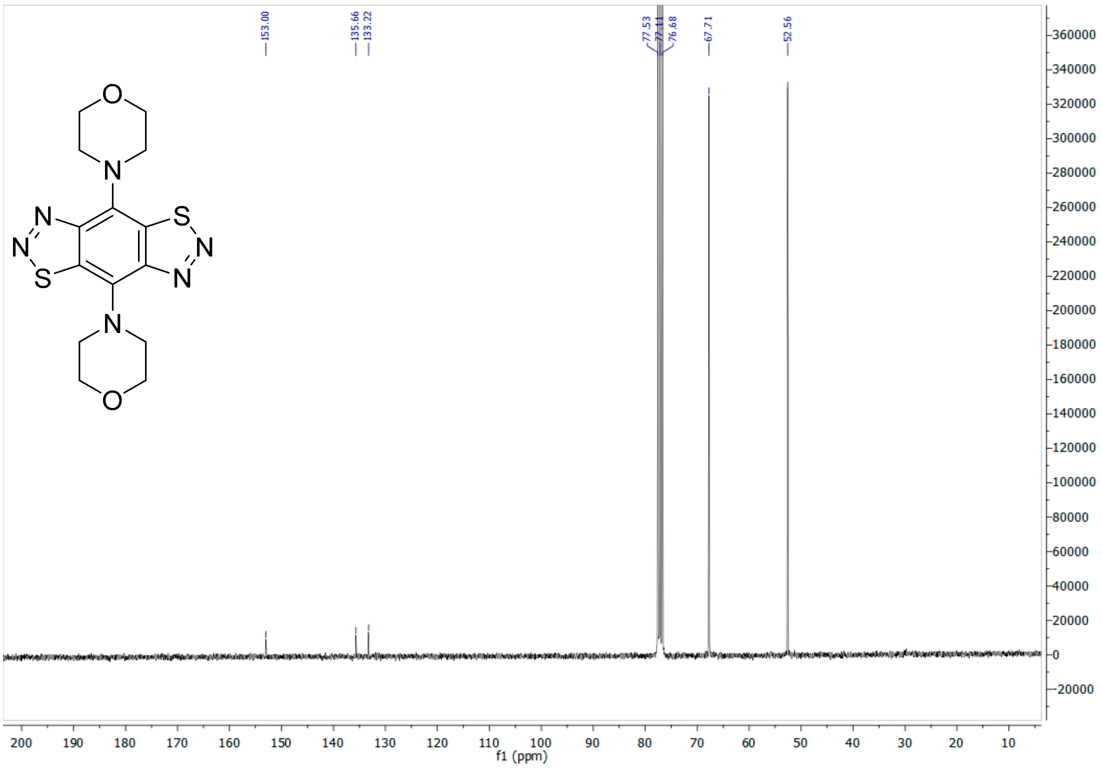

Display Report

Analysis Info

Analysis Name	D:\Data\Kolotyrkina\2022\Chmovsh\0727007.d	Acquisition Date	27.07.2022 16:08:20
Method	tune_100-1200.m	Operator	BDAL@DE
Sample Name	/SUSU Tim-543	Instrument / Ser#	maXis 43
Comment	C14H16N6O2S2 mH 365.0848 clb added CH3CN		

Acquisition Parameter

Source Type	ESI	Ion Polarity	Positive	Set Nebulizer	1.0 Bar
Focus	Active			Set Dry Heater	200 °C
Scan Begin	50 m/z	Set Capillary	4500 V	Set Dry Gas	4.0 l/min
Scan End	1800 m/z	Set End Plate Offset	-500 V	Set Divert Valve	Waste

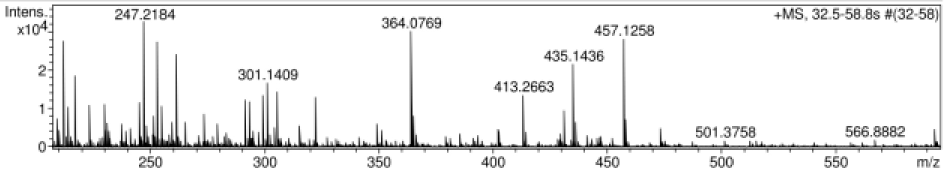

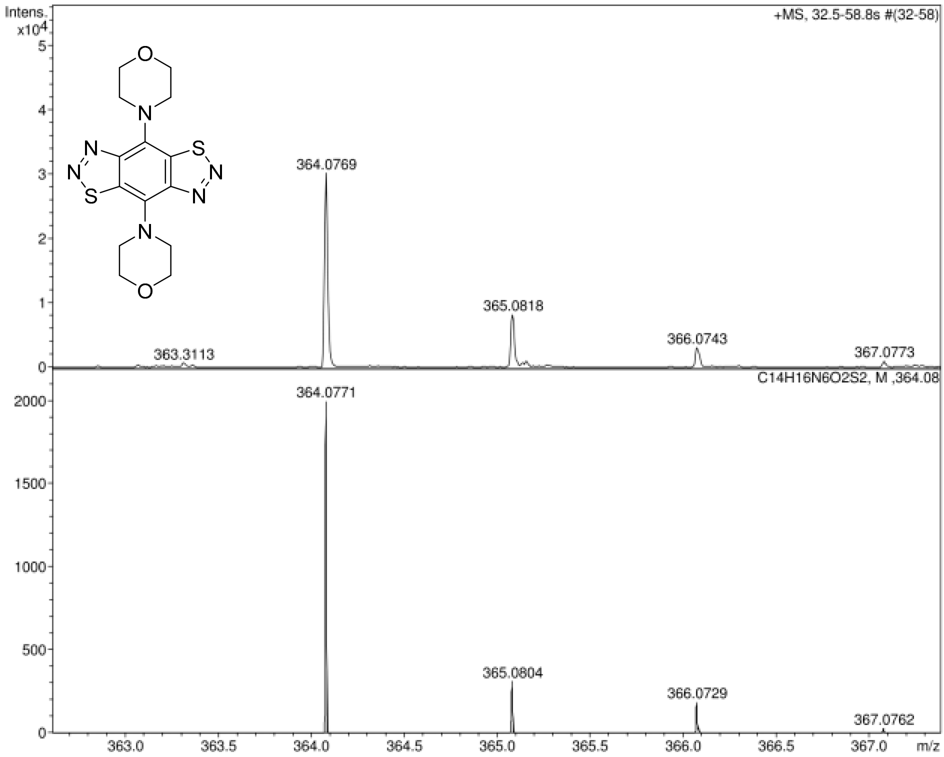

4,8-Di(piperidin-1-yl)benzo[1,2-d:4,5-d']bis([1,2,3]thiadiazole) (13b)

^{1}H NMR (300 MHz)

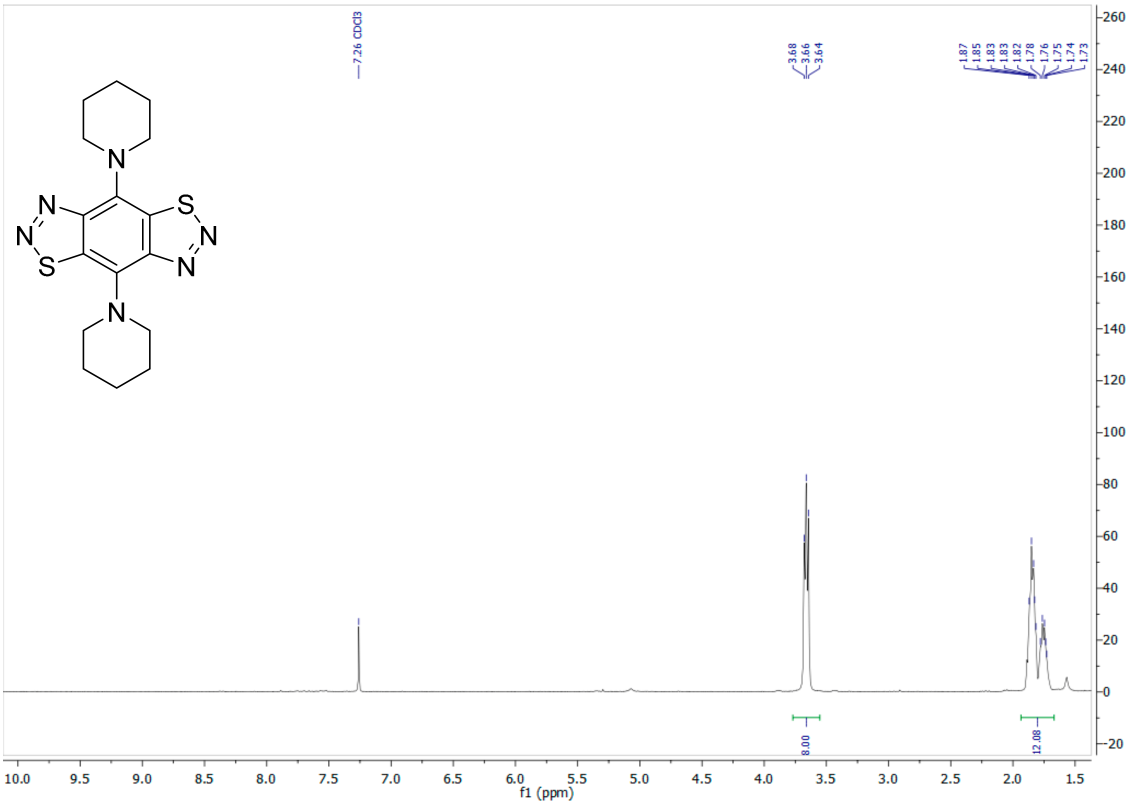

^{13}C NMR(75 MHz)

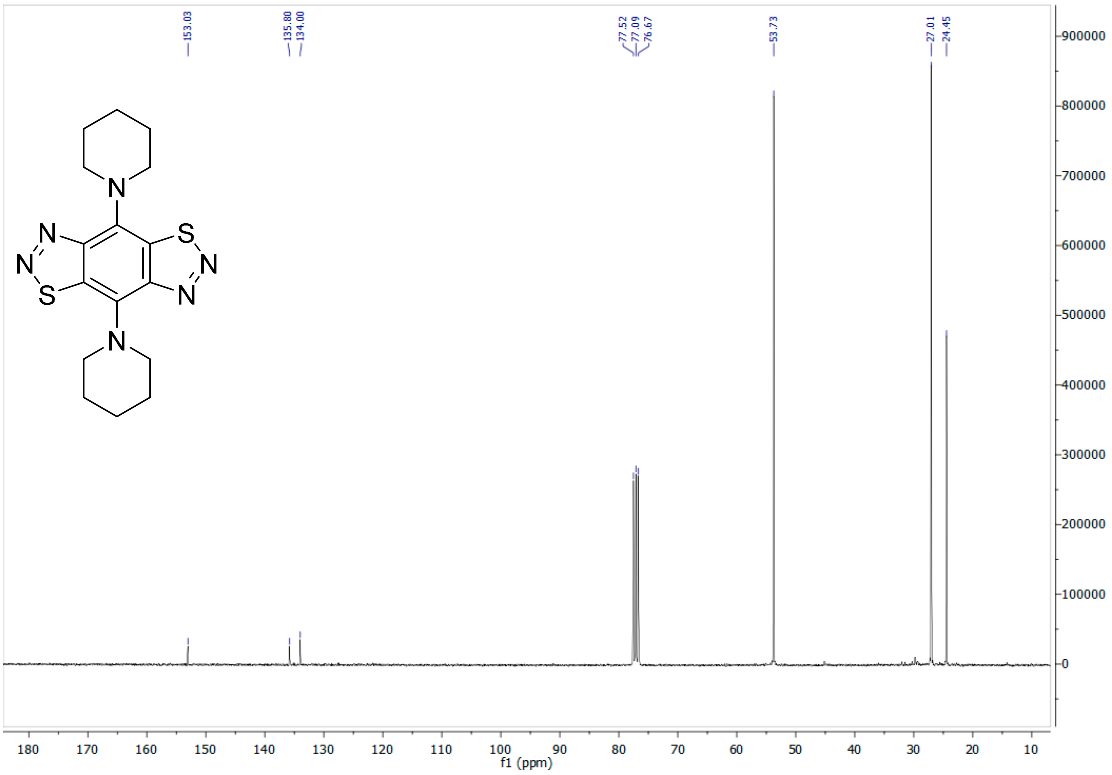

Display Report

Analysis Info

Analysis Name	D:\Data\Chizhov\Miscellaneous\Rakitin\Chmovzh\tim-547_&clblow.d	Acquisition Date	25.08.2022 15:20:18
Method	tune_low_1550.m	Operator	BDAL@DE
Sample Name	/SUSU Tim-547	Instrument / Ser#	maXis 43
Comment	CH3CN 100 %, dil. 200, calibrant added		

Acquisition Parameter

Source Type	ESI	Ion Polarity	Positive	Set Nebulizer	0.4 Bar
Focus	Not active			Set Dry Heater	180 °C
Scan Begin	50 m/z	Set Capillary	4500 V	Set Dry Gas	4.0 l/min
Scan End	1550 m/z	Set End Plate Offset	-500 V	Set Divert Valve	Source

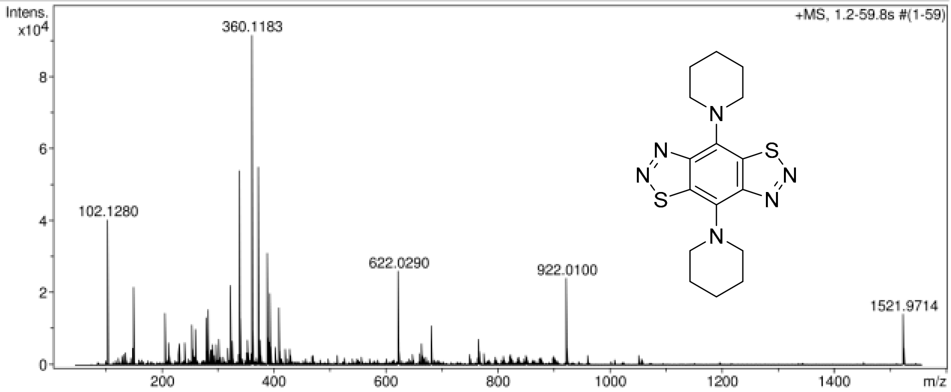

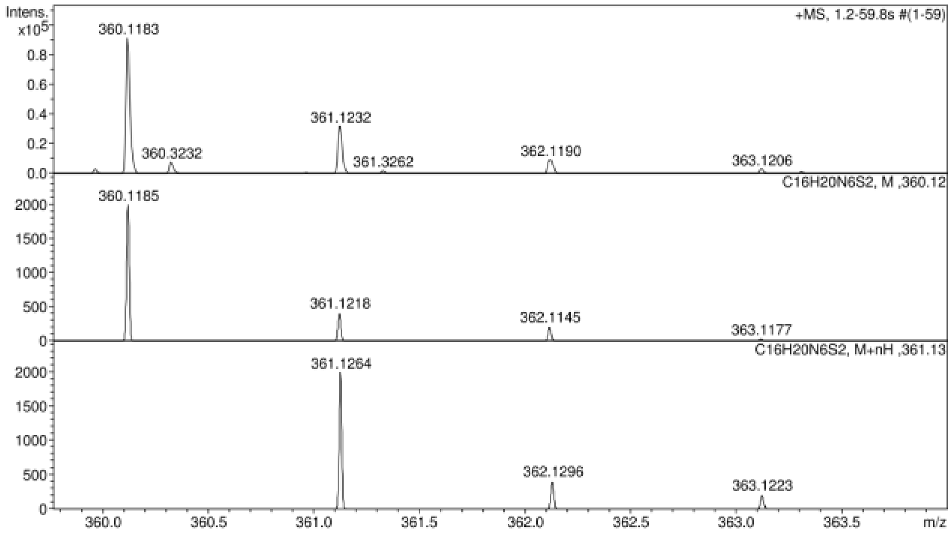

Bruker Compass DataAnalysis 4.0 printed: 25.08.2022 15:47:28 Page 1 of 1

4,8-Bis(phenylthio)benzo[1,2-d:4,5-d']bis([1,2,3]thiadiazole) (15a)

^{1}H NMR (300 MHz)

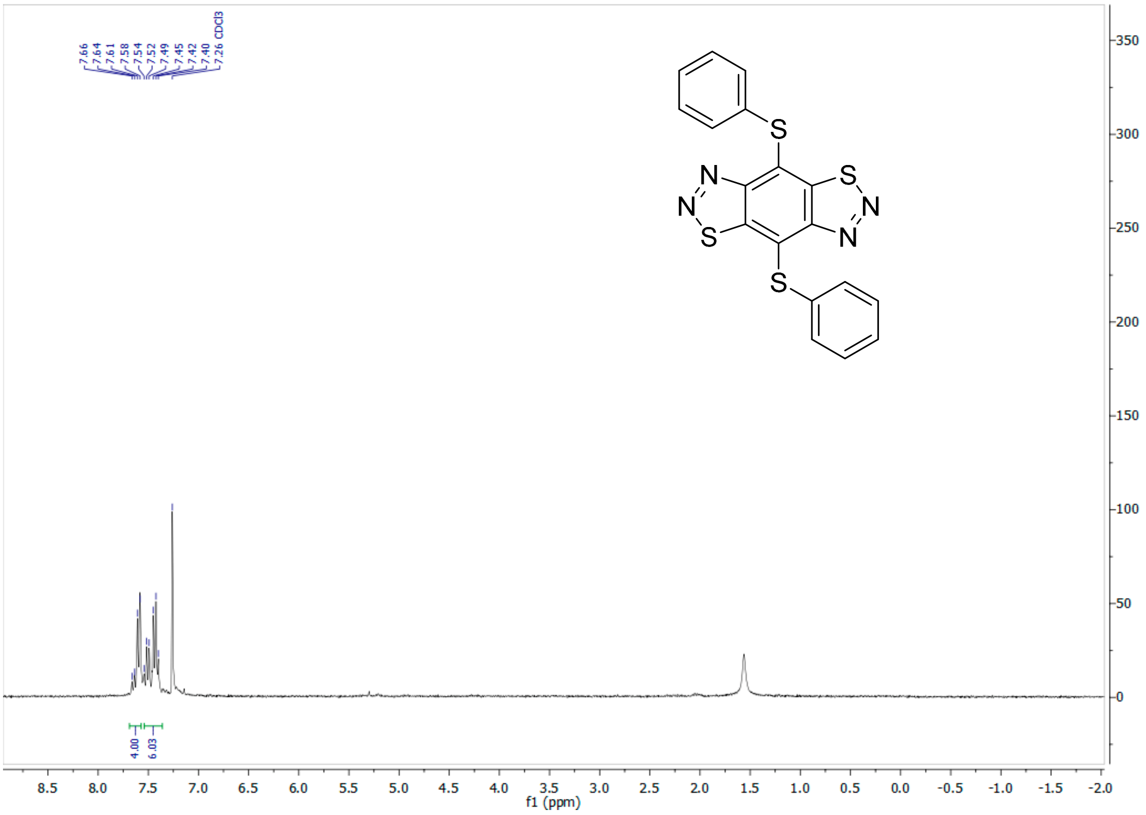

^{13}C NMR(75 MHz)

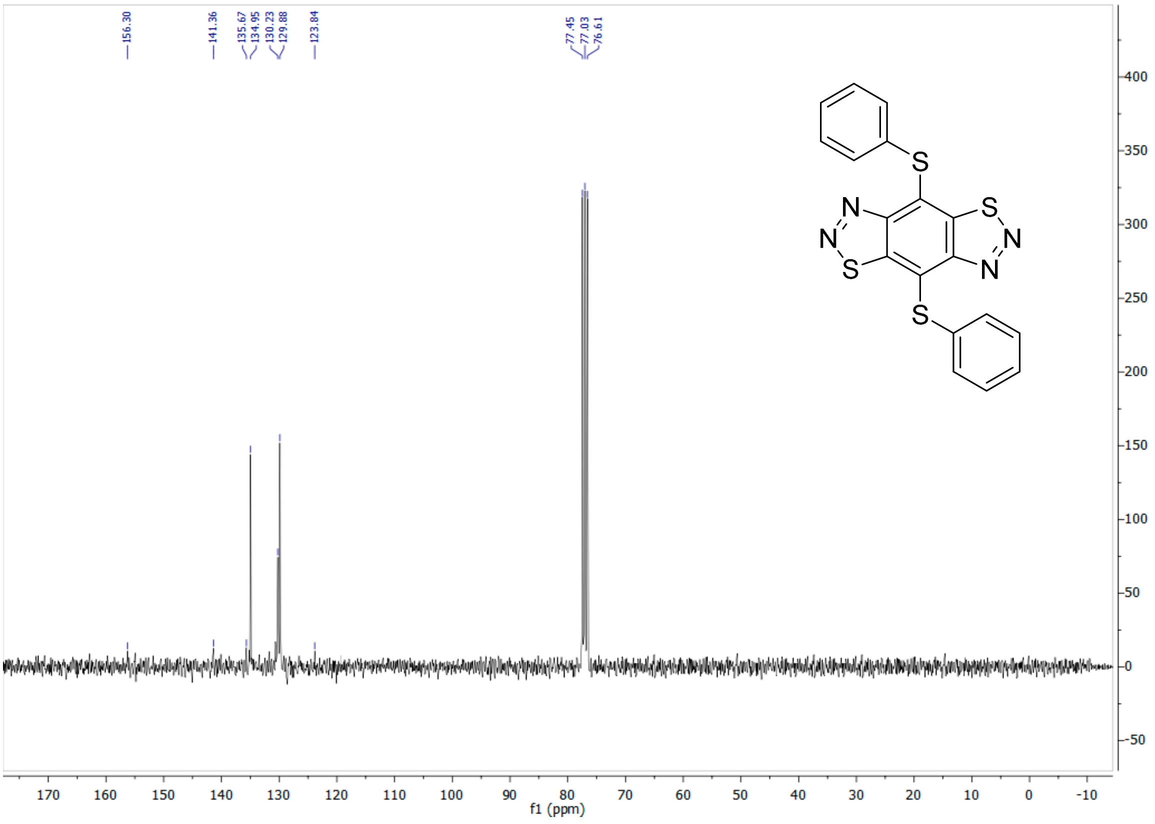

Display Report

Analysis Info

Analysis Name	D:\Data\Kolotyrkina\2022\Chmovsh\0608028.d	Acquisition Date	08.06.2022 16:45:25
Method	tune_50-1600_pos_15_12.m	Operator	BDAL@DE
Sample Name	/SUSU Tim-533	Instrument / Ser#	micrOTOF 10248
Comment	C18H10N4S2 mH 347.0419 clb added CH3CN		

Acquisition Parameter

Source Type	ESI	Ion Polarity	Positive	Set Nebulizer	0.4 Bar
Focus	Not active			Set Dry Heater	180 °C
Scan Begin	50 m/z	Set Capillary	4500 V	Set Dry Gas	4.0 l/min
Scan End	1600 m/z	Set End Plate Offset	-500 V	Set Divert Valve	Waste

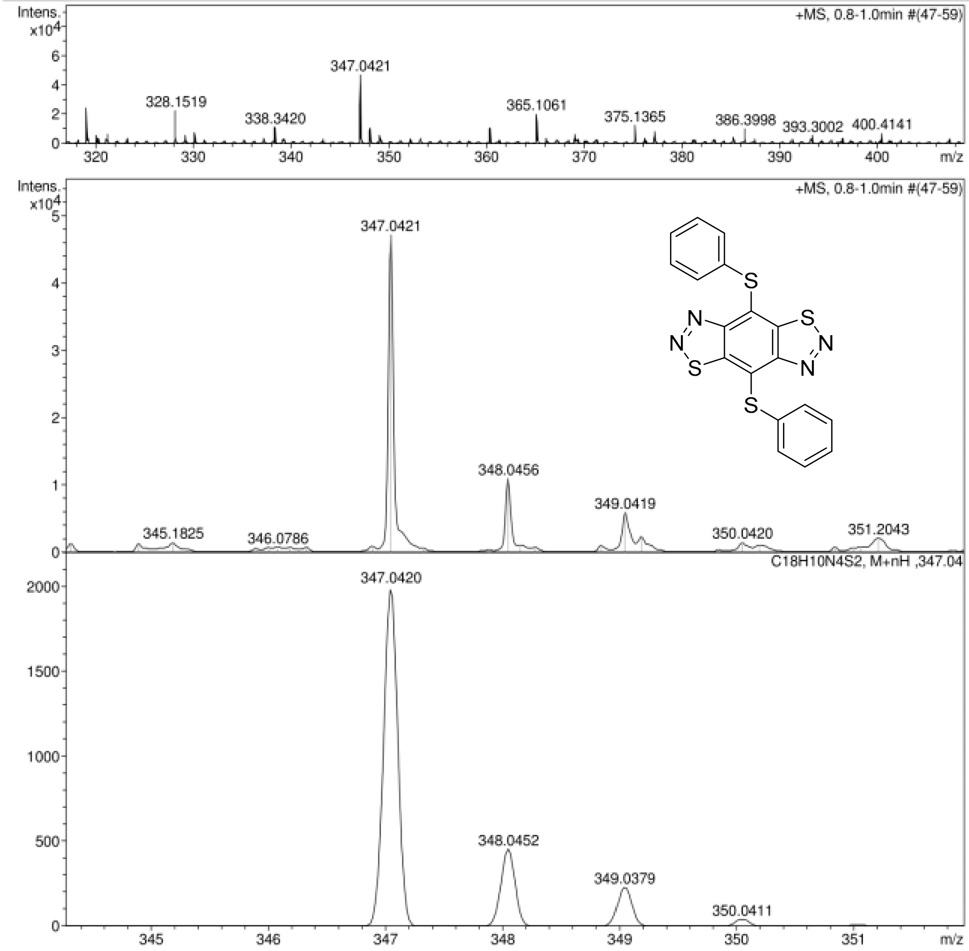

Bruker Compass DataAnalysis 4.0 printed: 08.06.2022 16:50:39 Page 1 of 1

4,8-Bis(hexylthio)benzo[1,2-d:4,5-d']bis([1,2,3]thiadiazole) (15b)

^{1}H NMR (300 MHz)

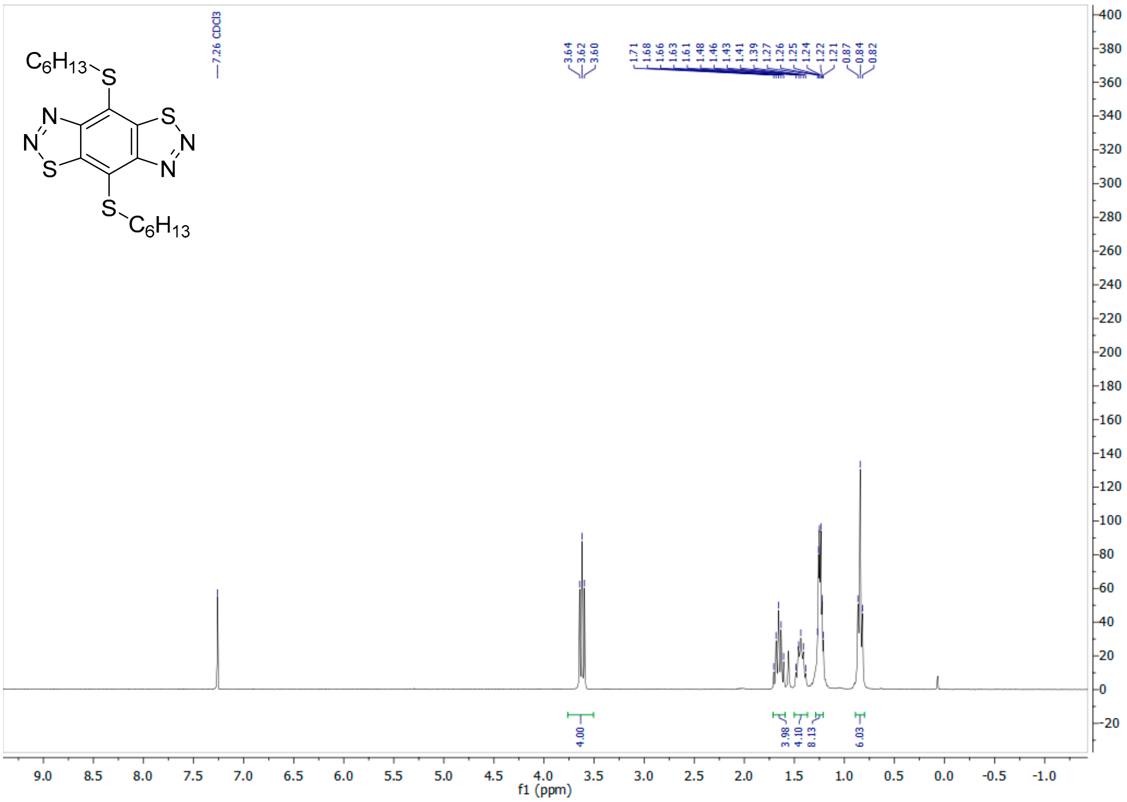

^{13}C NMR(75 MHz)

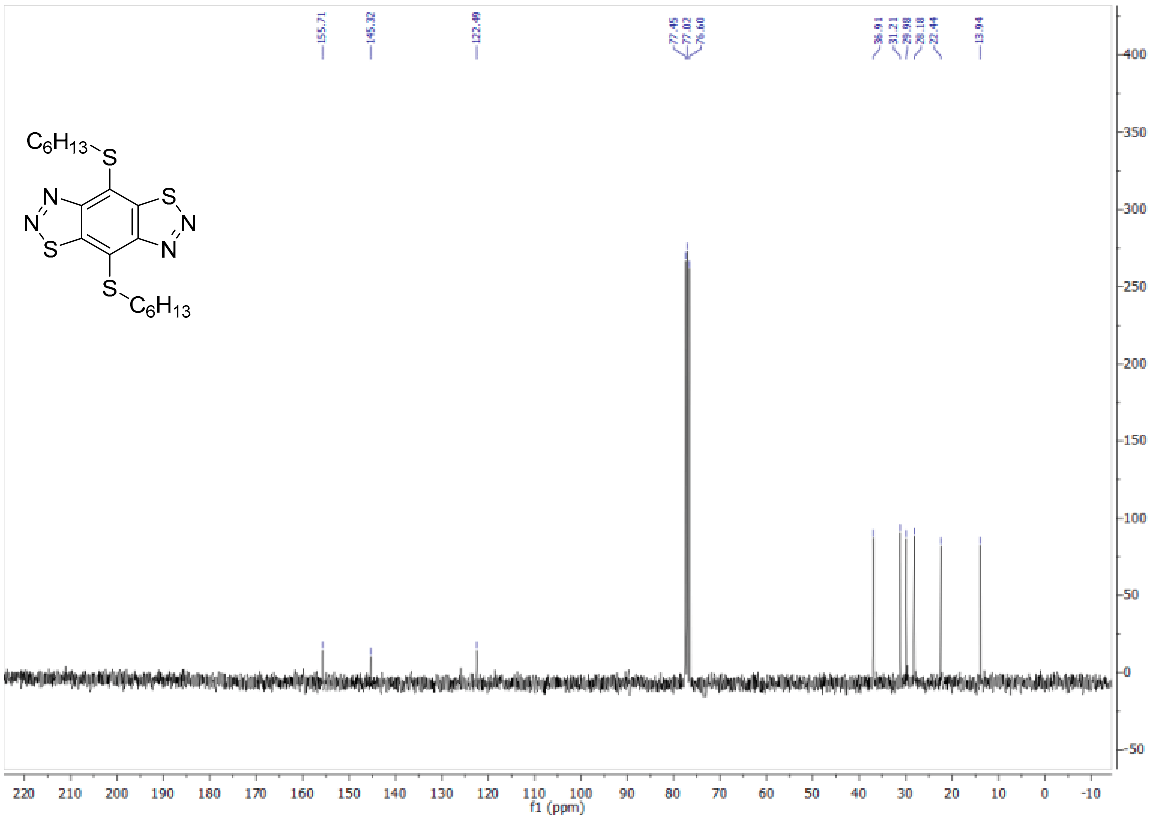

155.71
145.32
122.49
77.45
77.02
76.60
36.91
31.21
29.98
28.18
22.44
13.94
f1 (ppm)

Display Report

Analysis Info

Analysis Name	D:\Data\Kolotyrkina\2022\Chmovsh\0526020.d	Acquisition Date	26.05.2022 15:05:39
Method	tune_50-1600_pos_15_12.m	Operator	BDAL@DE
Sample Name	/SUSU Tim-526	Instrument / Ser#	micrOTOF 10248
Comment	C18H26N4S4 mH 427.1124 calibrant added CH3CN		

Acquisition Parameter

Source Type	ESI	Ion Polarity	Positive	Set Nebulizer	0.4 Bar
Focus	Not active			Set Dry Heater	180 °C
Scan Begin	50 m/z	Set Capillary	4500 V	Set Dry Gas	4.0 l/min
Scan End	1600 m/z	Set End Plate Offset	-500 V	Set Divert Valve	Waste

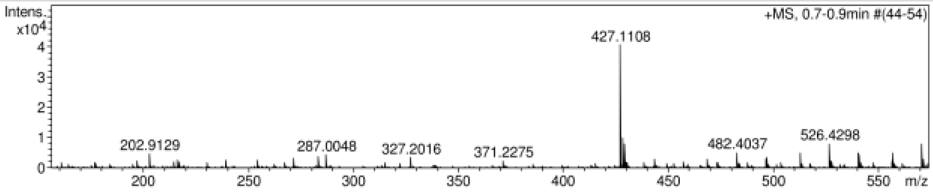

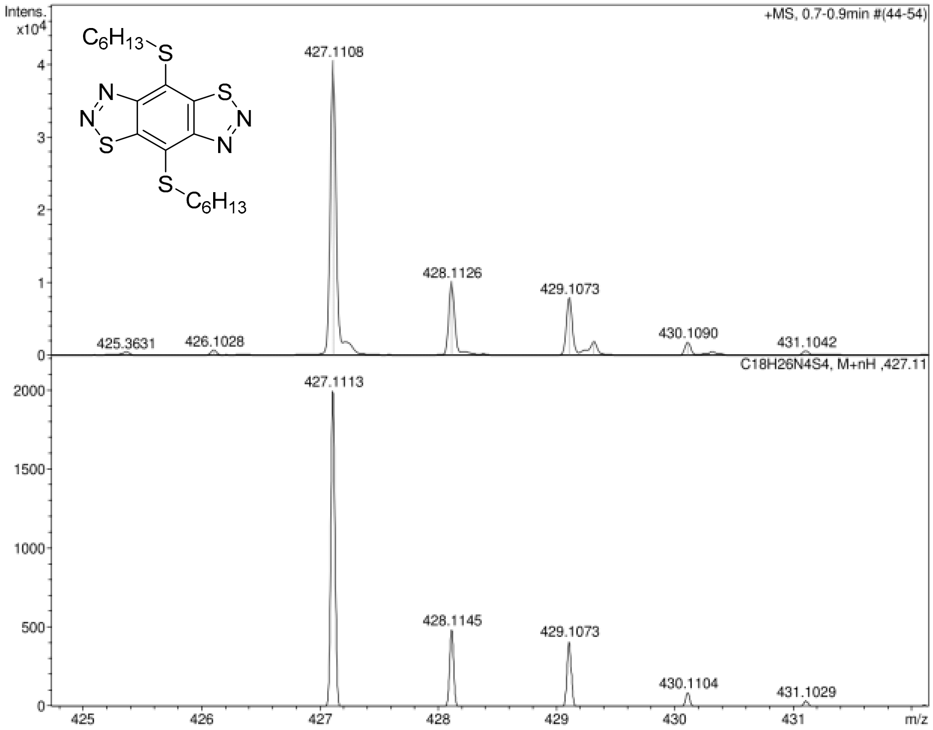

4,8-Bis(dodecylthio)benzo[1,2-d:4,5-d']bis([1,2,3]thiadiazole) (15c)

^{1}H NMR (300 MHz)

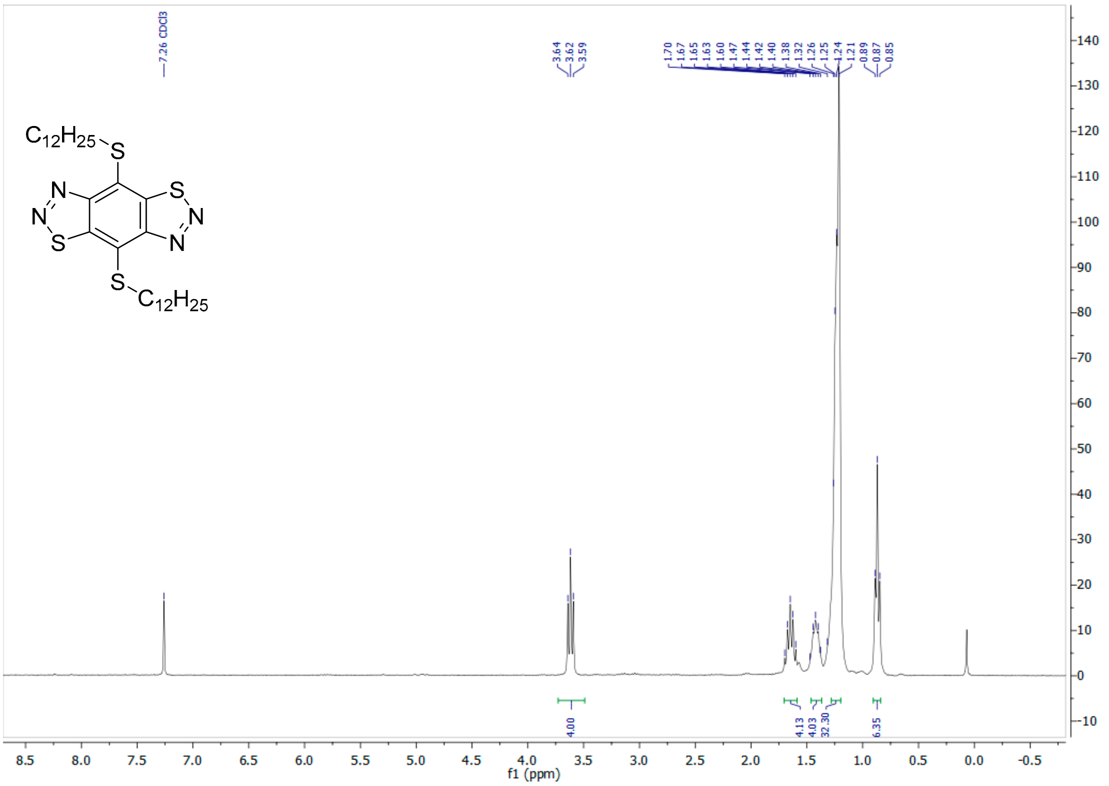

^{13}C NMR(75 MHz)

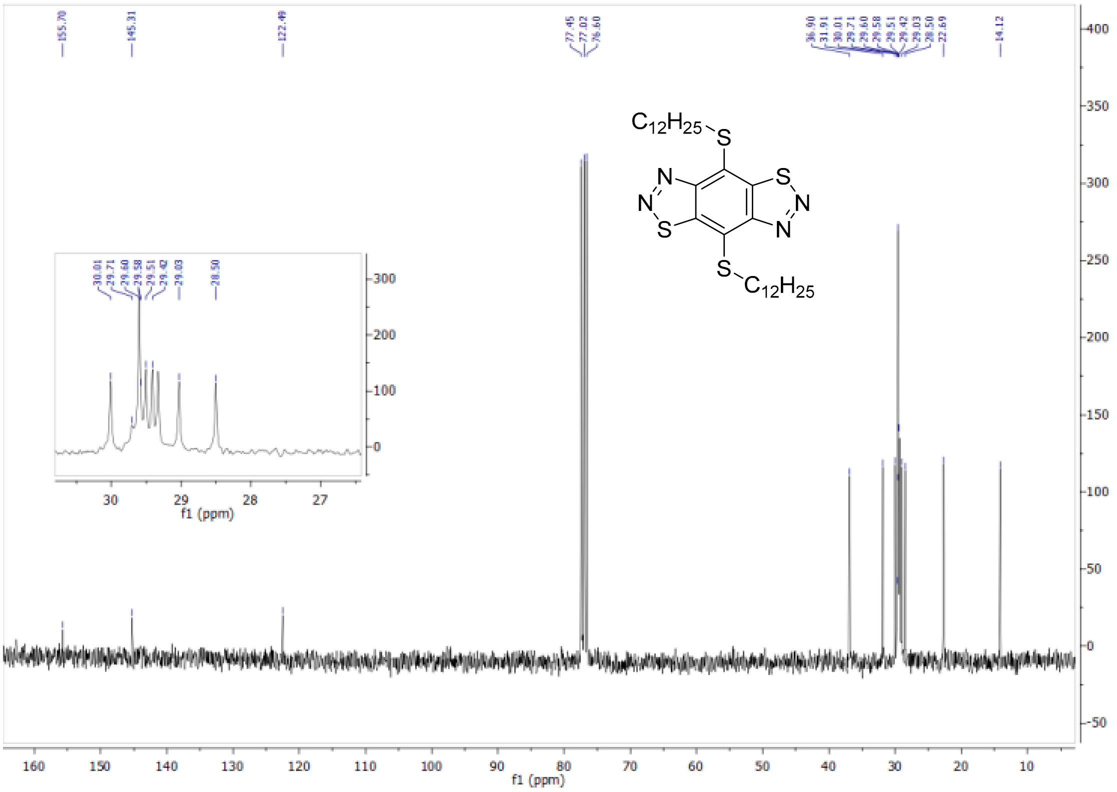

Display Report

Analysis Info

Analysis Name	D:\Data\Kolotyrkina\2022\Chmovsh\0526023.d	Acquisition Date	26.05.2022 15:27:22
Method	tune_50-1600_pos_15_12.m	Operator	BDAL@DE
Sample Name	/SUSU Tim-527	Instrument / Ser#	micrOTOF 10248
Comment	C30H50N4S4 mH 595.3002 calibrant added CH3CN		

Acquisition Parameter

Source Type	ESI	Ion Polarity	Positive	Set Nebulizer	0.4 Bar
Focus	Not active			Set Dry Heater	180 °C
Scan Begin	50 m/z	Set Capillary	4500 V	Set Dry Gas	4.0 l/min
Scan End	1600 m/z	Set End Plate Offset	-500 V	Set Divert Valve	Waste

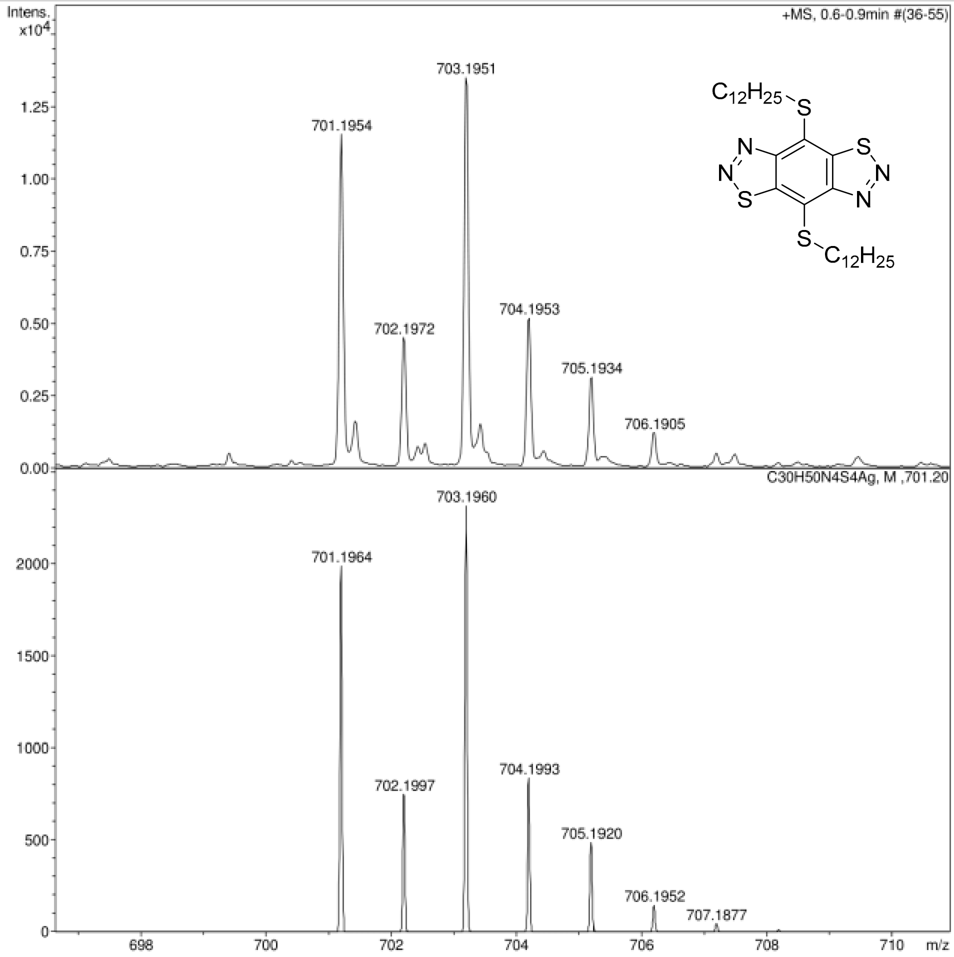

4-Bromo-8-(thiophen-2-yl)benzo[1,2-d:4,5-d']bis([1,2,3]thiadiazole) (18a)

^{1}H NMR (300 MHz)

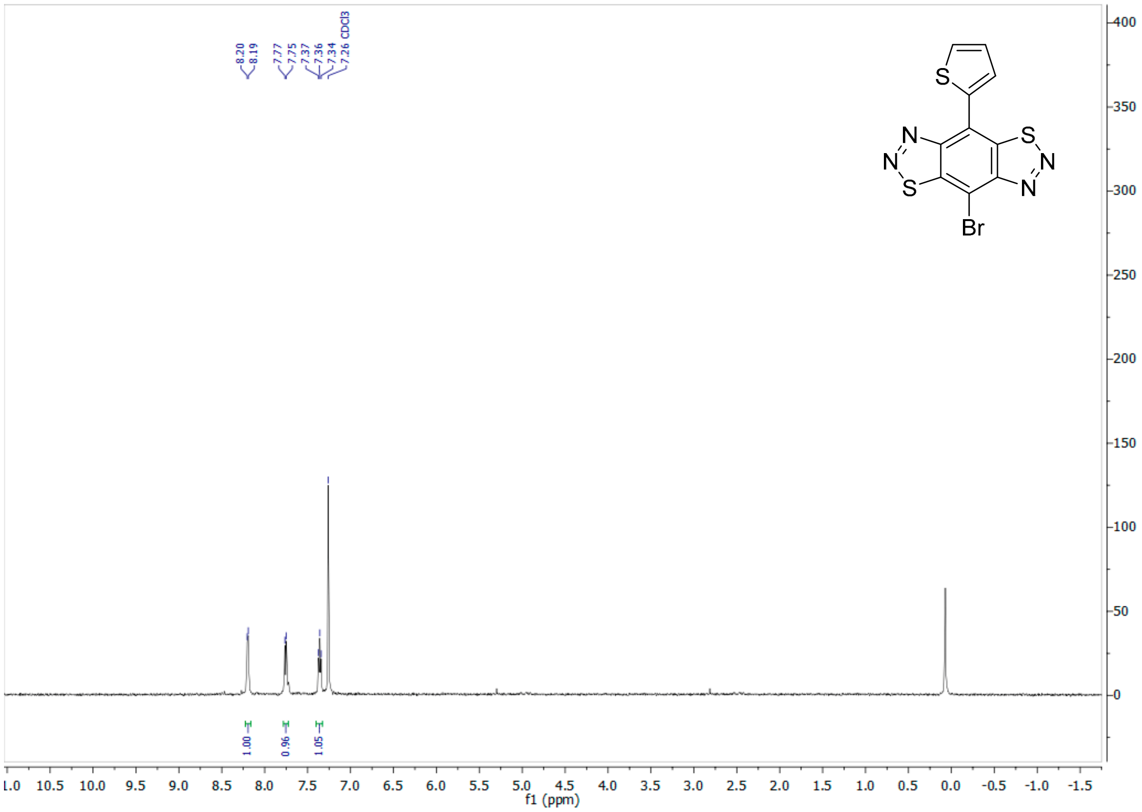

^{13}C NMR(75 MHz)

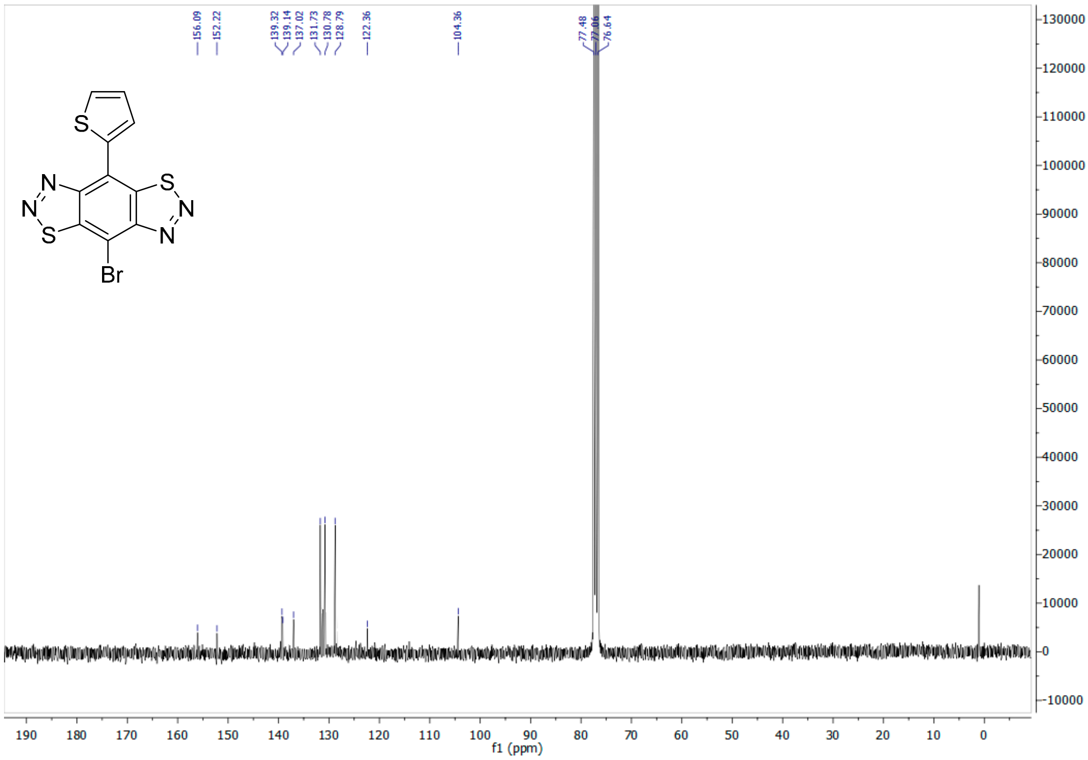

Display Report

Analysis Info

Analysis Name	D:\Data\Kolotyrkina\2022\Kudryashov\0920002.d	Acquisition Date	20.09.2022 11:28:31
Method	tune_50-1600_pos_15_12.m	Operator	BDAL@DE
Sample Name	/SUSU Tim-554	Instrument / Ser#	micrOTOF 10248
Comment	C10H3BrN4S3 mH 354.8775 calibrant added CH3CN		

Acquisition Parameter

Source Type	ESI	Ion Polarity	Positive	Set Nebulizer	0.4 Bar
Focus	Not active			Set Dry Heater	180 °C
Scan Begin	50 m/z	Set Capillary	4500 V	Set Dry Gas	4.0 l/min
Scan End	1600 m/z	Set End Plate Offset	-500 V	Set Divert Valve	Waste

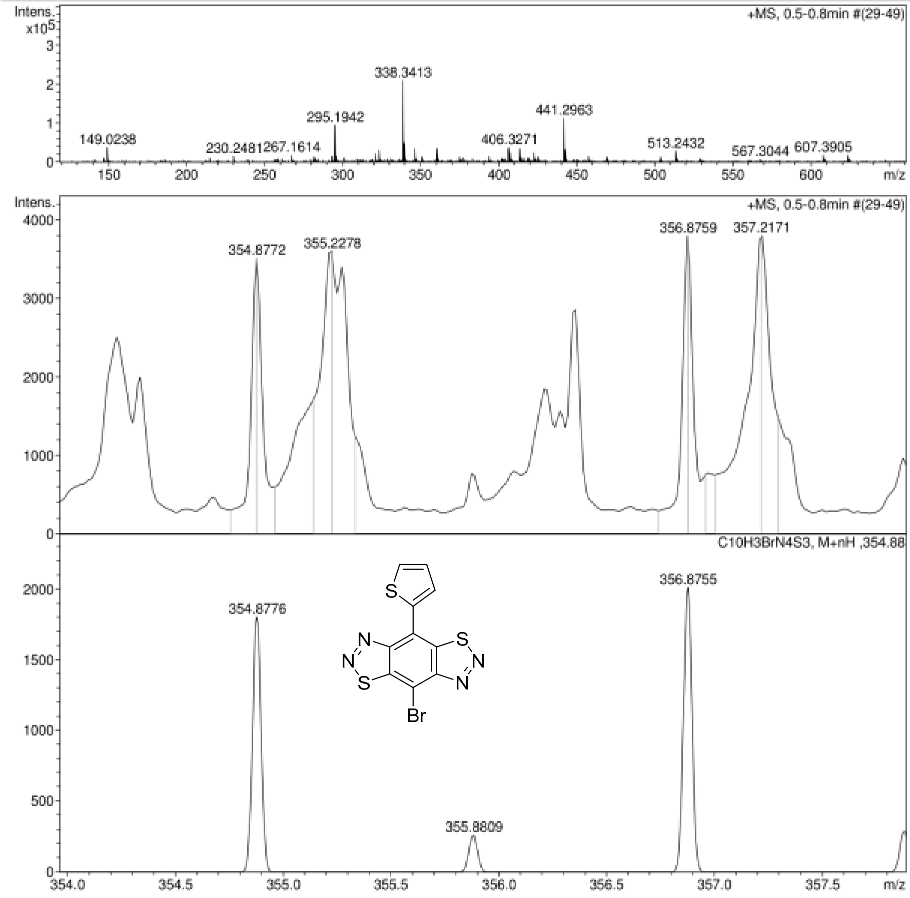

4-Bromo-8-(4-hexylthiophen-2-yl)benzo[1,2-d:4,5-d']bis([1,2,3]thiadiazole) (18b)

^{1}H NMR (300 MHz)

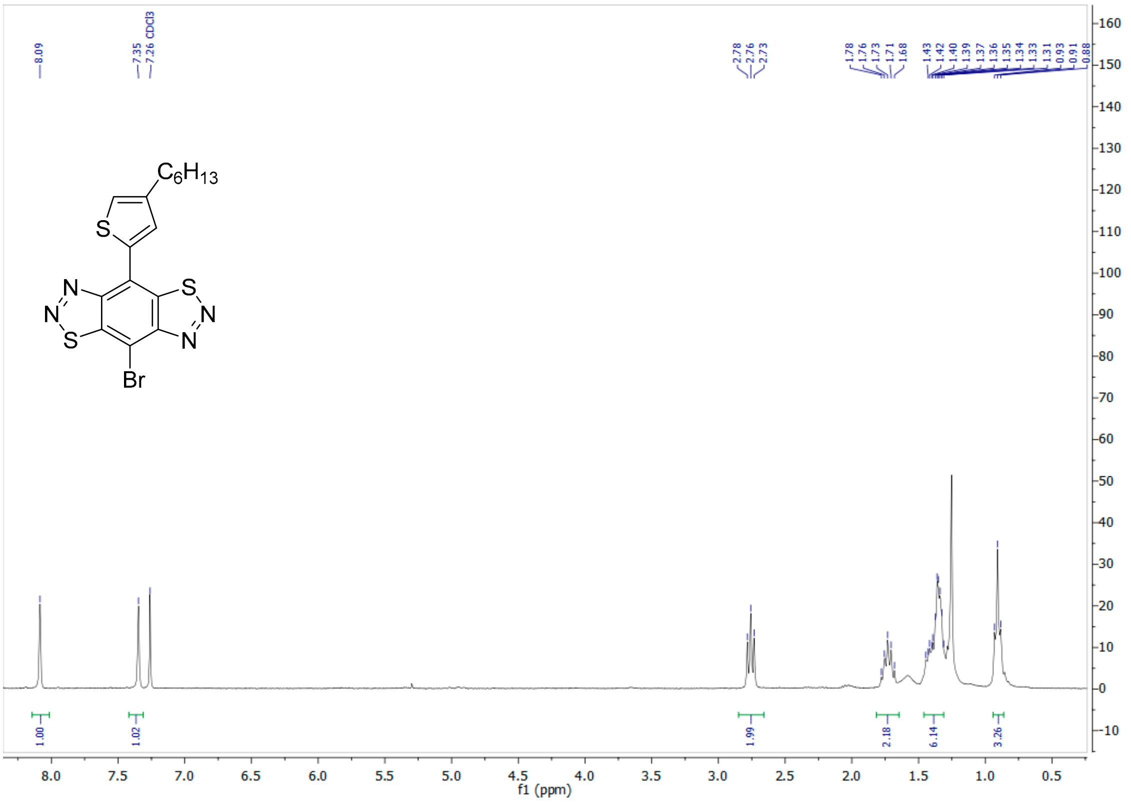

^{13}C NMR(75 MHz)

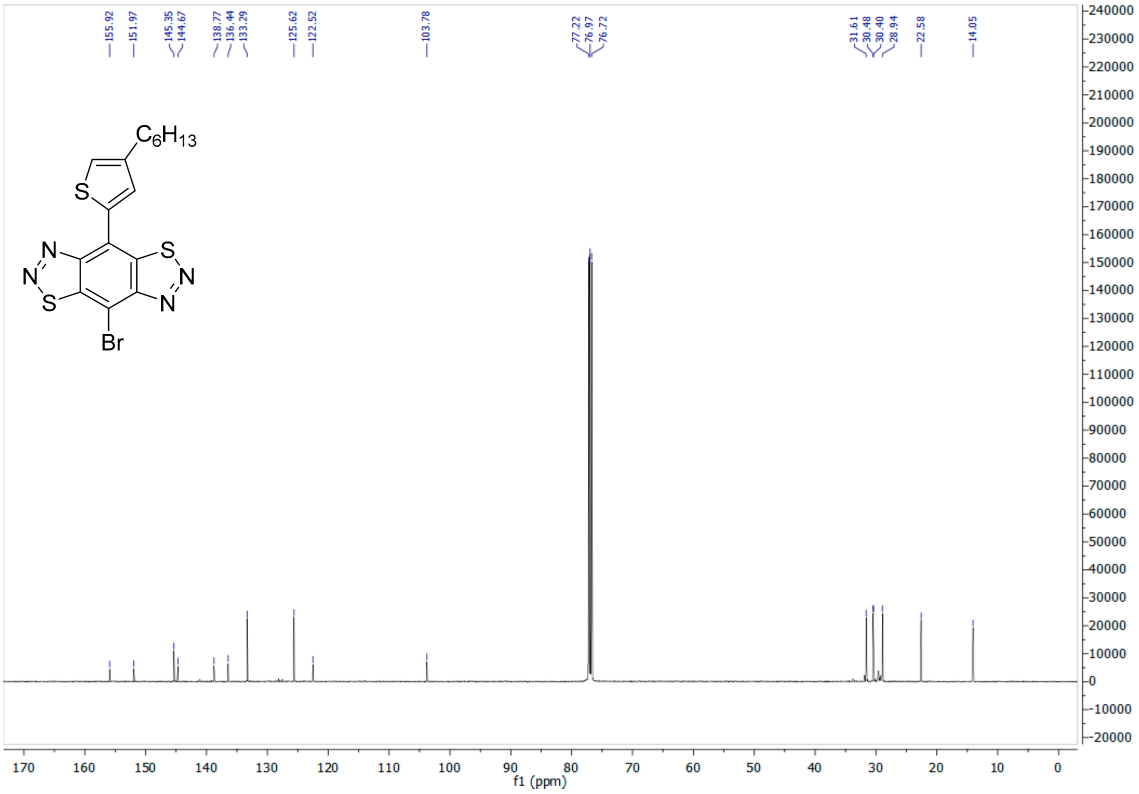

155.92
151.97
145.35
144.67
138.77
136.44
133.29
125.62
122.52
103.78
77.22
76.97
76.72
31.61
30.48
30.40
28.94
22.58
14.05
C6H13
S
N
N
S
S
N
N
Br
f1 (ppm)

Display Report

Analysis Info

Analysis Name	D:\Data\Kolotyrkina\2022\Chmovsh\0707035.d	Acquisition Date	07.07.2022 16:59:13
Method	tune_50-1600_pos_15_12.m	Operator	BDAL@DE
Sample Name	/SUSU Tim-537	Instrument / Ser#	micrOTOF 10248
Comment	C16H15BrN4S3 mH 438.9714 calibrant added CH3CN		

Acquisition Parameter

Source Type	ESI	Ion Polarity	Positive	Set Nebulizer	0.4 Bar
Focus	Not active			Set Dry Heater	180 °C
Scan Begin	50 m/z	Set Capillary	4500 V	Set Dry Gas	4.0 l/min
Scan End	1600 m/z	Set End Plate Offset	-500 V	Set Divert Valve	Waste

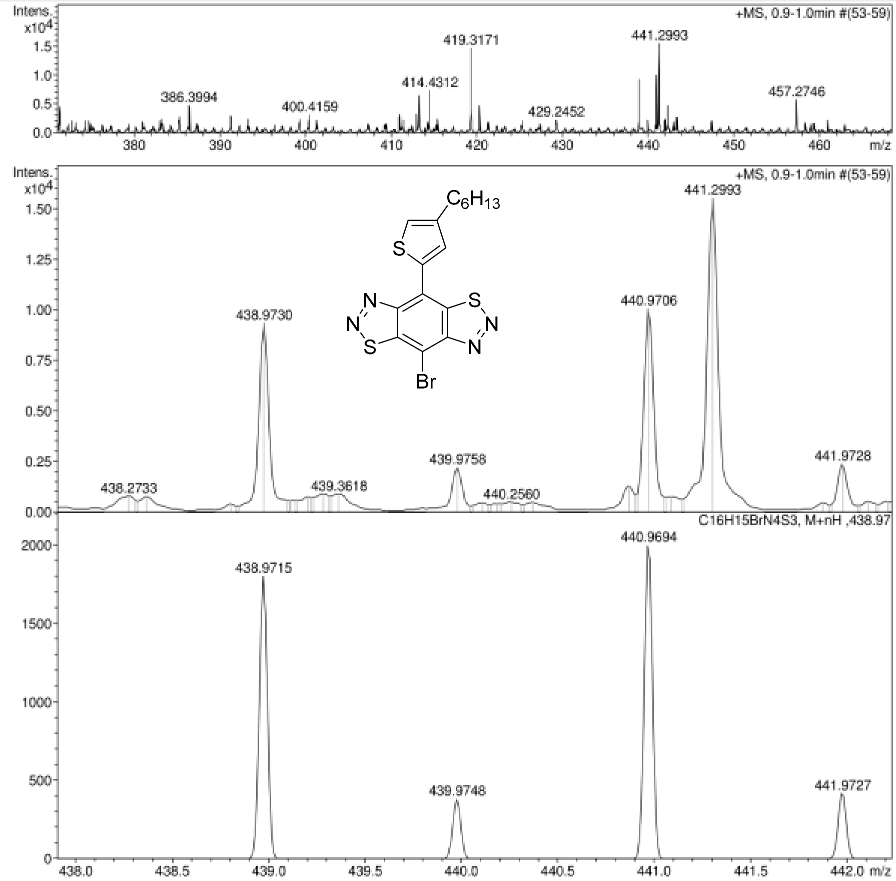

4-([2,2'-Bithiophen]-5-yl)-8-bromobenzo[1,2-d:4,5-d']bis([1,2,3]thiadiazole) (18c)

^{1}H NMR (300 MHz)

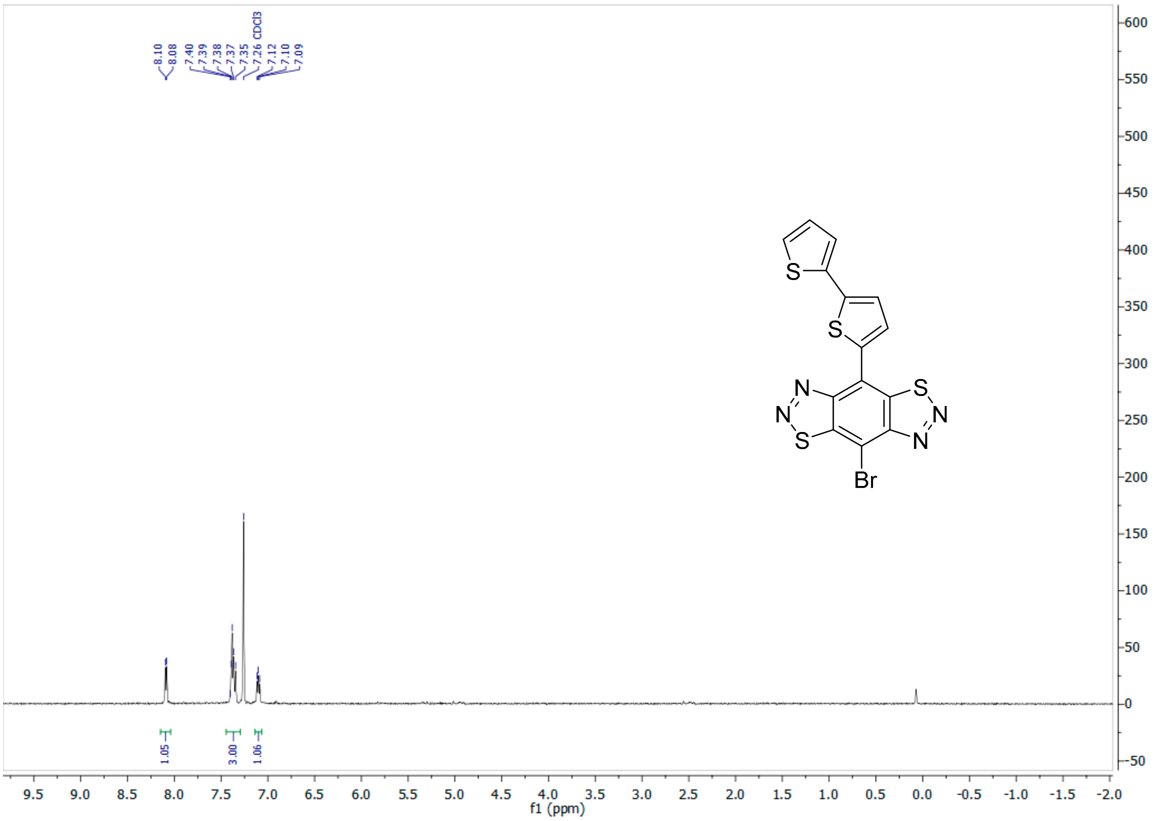

^{13}C NMR(75 MHz)

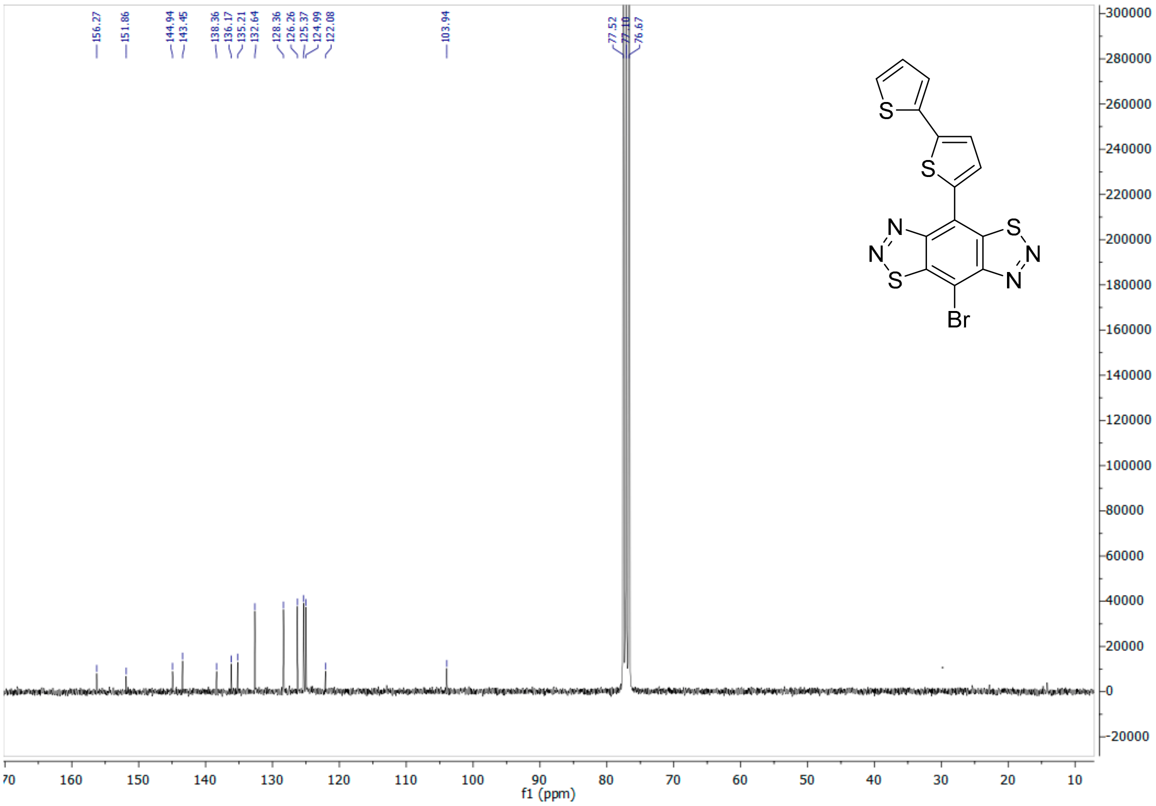

Display Report

Analysis Info

Analysis Name	D:\Data\Chizhov\Rakitin\Chmovzh\tim-535_&clb.d	Acquisition Date	29.06.2022 11:45:24
Method	tune_wide.m	Operator	BDAL@DE
Sample Name	/SUSU Tim-535	Instrument / Ser#	micrOTOF 10248
Comment	CH3CN 100 %, dil. 20, calibrant added		

Acquisition Parameter

Source Type	ESI	Ion Polarity	Positive	Set Nebulizer	0.4 Bar
Focus	Not active			Set Dry Heater	180 °C
Scan Begin	50 m/z	Set Capillary	4500 V	Set Dry Gas	4.0 l/min
Scan End	3000 m/z	Set End Plate Offset	-500 V	Set Divert Valve	Waste

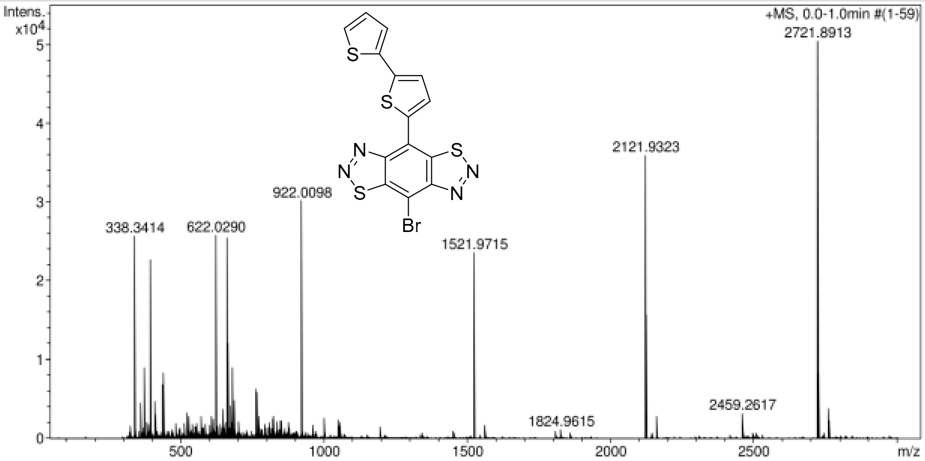

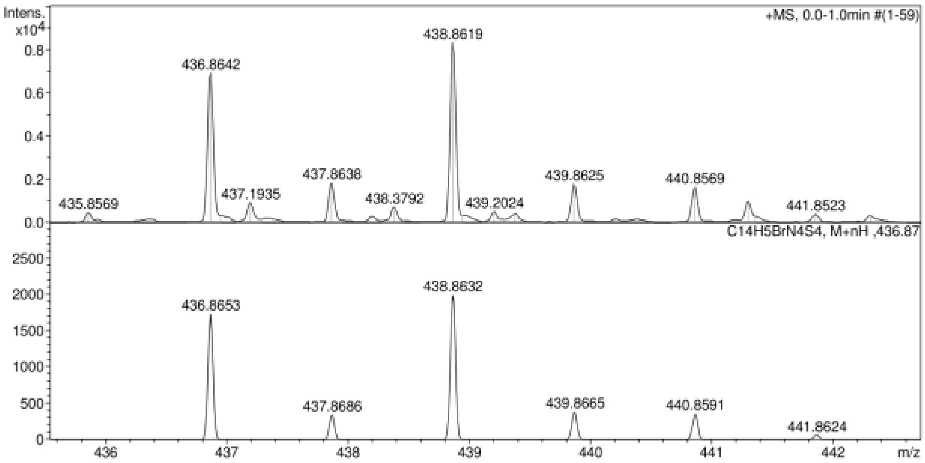

4-Bromo-8-(5-(2-ethylhexyl)thiophen-2-yl)benzo[1,2-d:4,5-d']bis([1,2,3]thiadiazole) (18d)

^{1}H NMR (300 MHz)

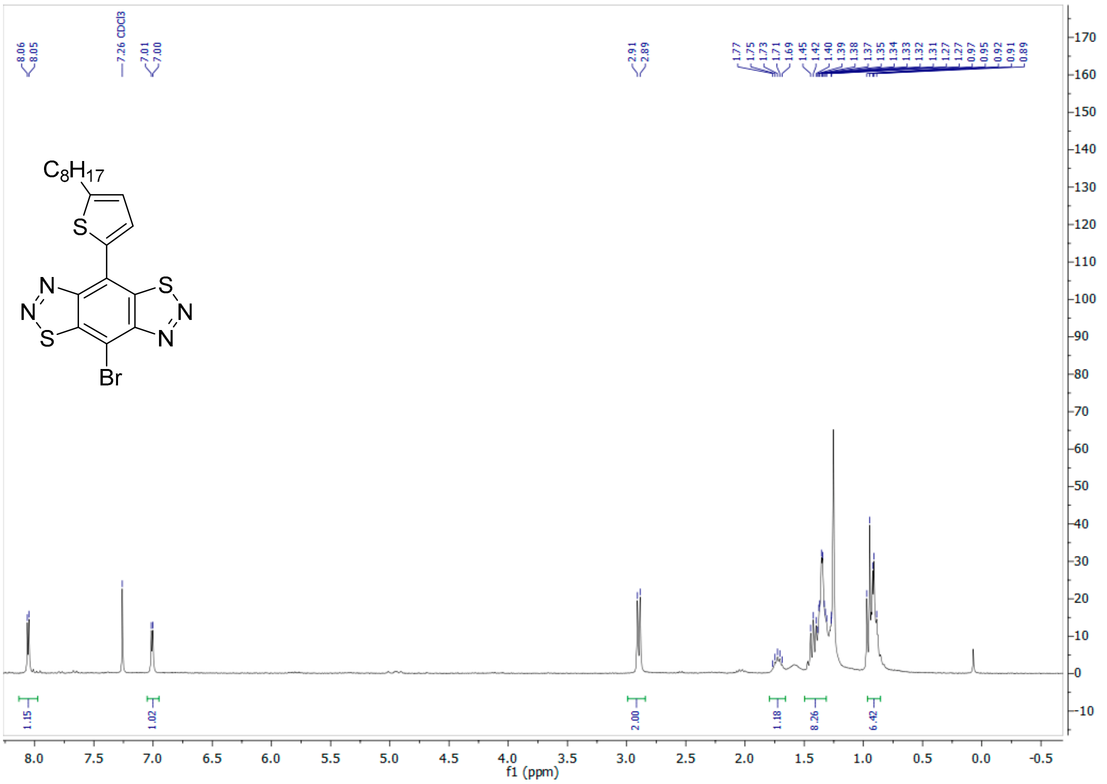

^{13}C NMR(75 MHz)

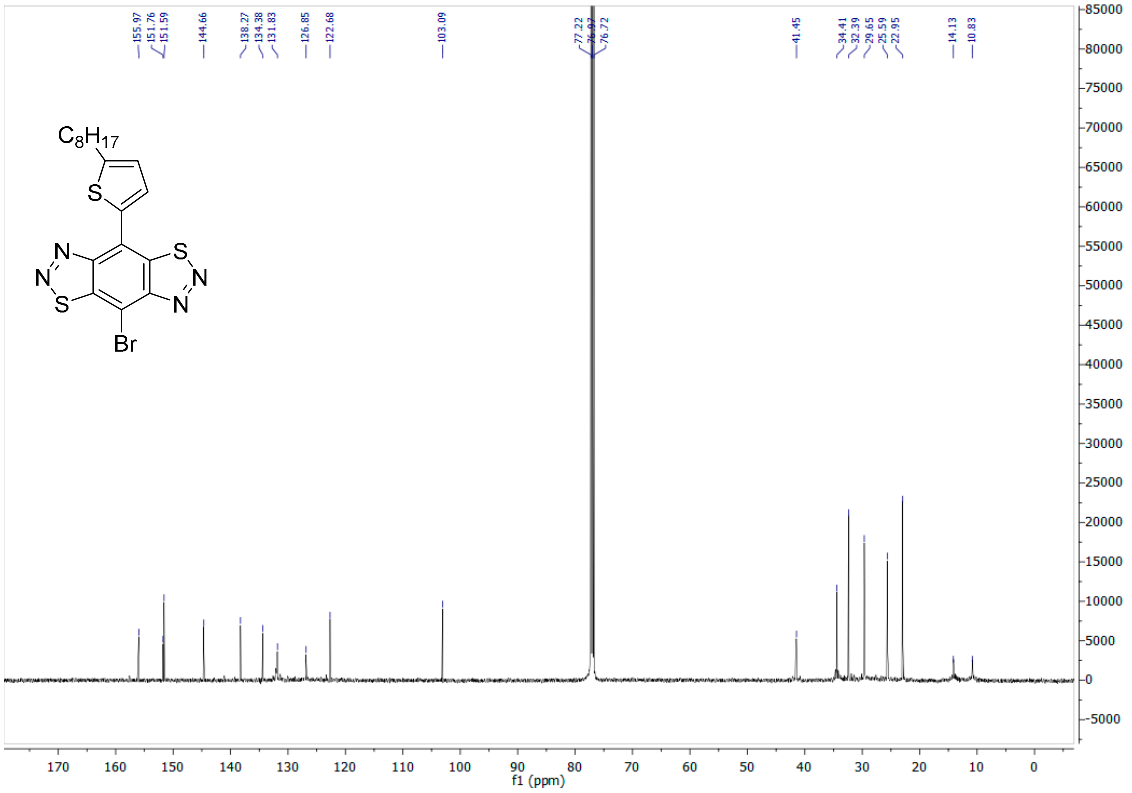

155.97
151.76
151.59
144.66
138.27
134.38
131.83
126.85
122.68
103.09
77.22
76.97
76.72
41.45
34.41
32.39
29.65
25.59
22.95
14.13
10.83
C8H17
S
N
N
S
S
N
N
Br
f1 (ppm)

Display Report

Analysis Info

Analysis Name	D:\Data\Chizhov\Miscellaneous\Rakitin\Chmovzh\tim-549_&clblow.d	Acquisition Date	25.08.2022 15:40:33
Method	tune_low_1550.m	Operator	BDAL@DE
Sample Name	/SUSU Tim-549	Instrument / Ser#	maXis 43
Comment	CH3CN 100 %, dil. 20, calibrant added		

Acquisition Parameter

Source Type	ESI	Ion Polarity	Positive	Set Nebulizer	0.4 Bar
Focus	Not active			Set Dry Heater	180 °C
Scan Begin	50 m/z	Set Capillary	4500 V	Set Dry Gas	4.0 l/min
Scan End	1550 m/z	Set End Plate Offset	-500 V	Set Divert Valve	Source

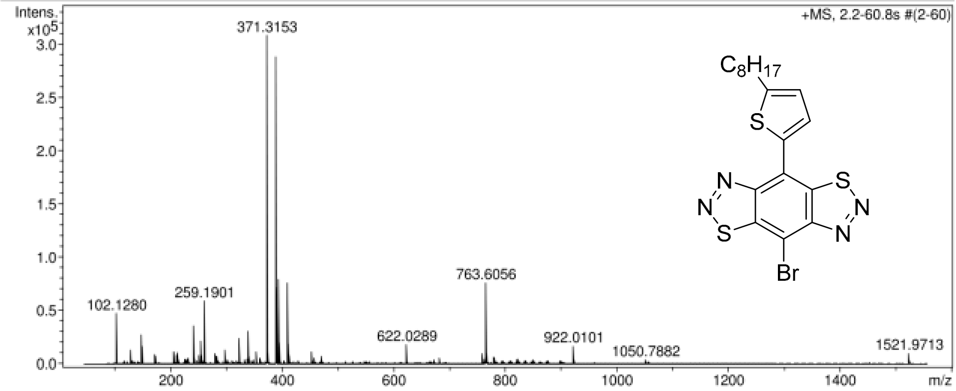

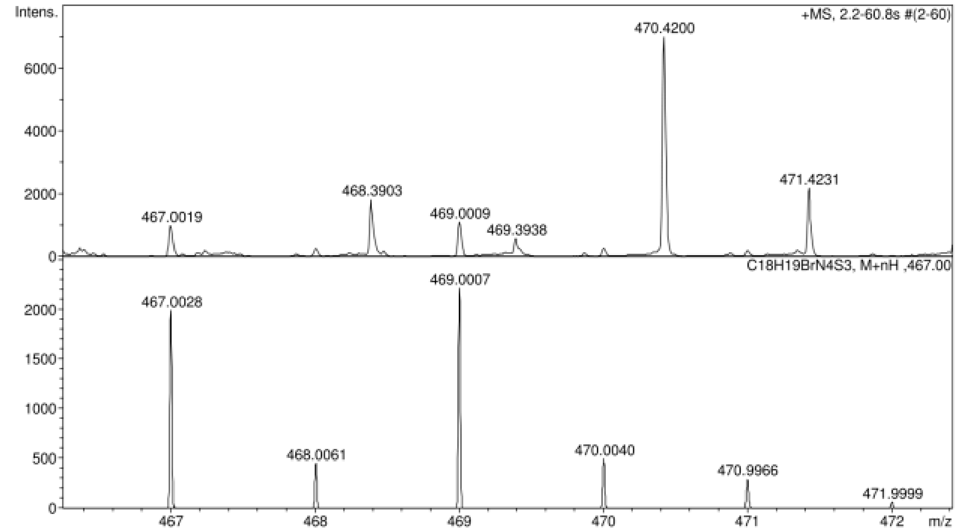

4-Bromo-8-phenylbenzo[1,2-d:4,5-d']bis([1,2,3]thiadiazole) (18e)

^{1}H NMR (300 MHz)

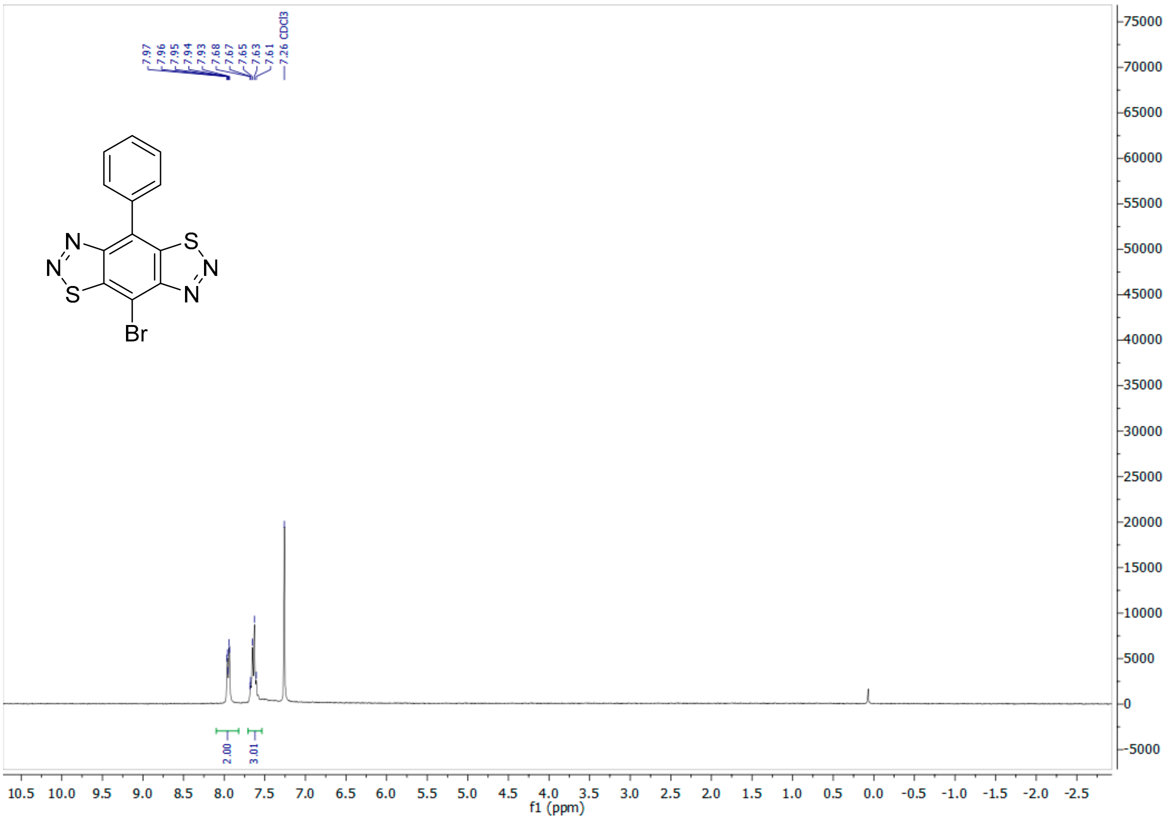

^{13}C NMR(75 MHz)

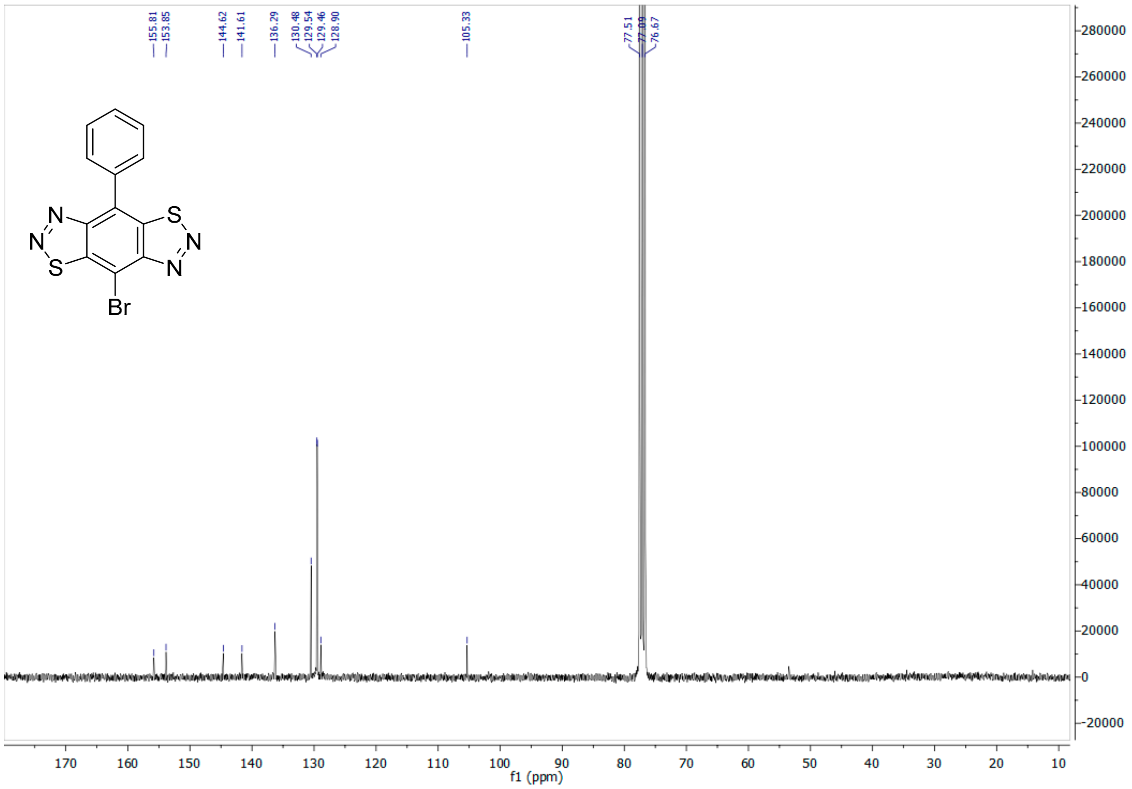
155.81
153.85
144.62
141.61
136.29
130.48
129.54
129.46
128.90
105.33
77.51
77.09
76.67
N
N
S
S
N
N
Br
f1 (ppm)

Display Report

Analysis Info

Analysis Name	D:\Data\Kolotyrkina\2022\Chmovsh\0921004.d	Acquisition Date	21.09.2022 10:25:32
Method	tune_50-1600_pos_15_12.m	Operator	BDAL@DE
Sample Name	/SUSU Tim-556	Instrument / Ser#	micrOTOF 10248
Comment	C12H5BrN4S2 mH 348.9211 calibrant added CH3OH		

Acquisition Parameter

Source Type	ESI	Ion Polarity	Positive	Set Nebulizer	0.4 Bar
Focus	Not active			Set Dry Heater	180 °C
Scan Begin	50 m/z	Set Capillary	4500 V	Set Dry Gas	4.0 l/min
Scan End	1600 m/z	Set End Plate Offset	-500 V	Set Divert Valve	Waste

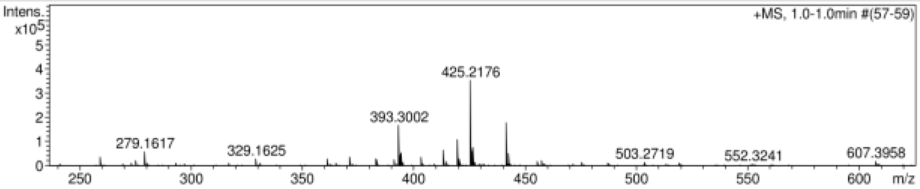

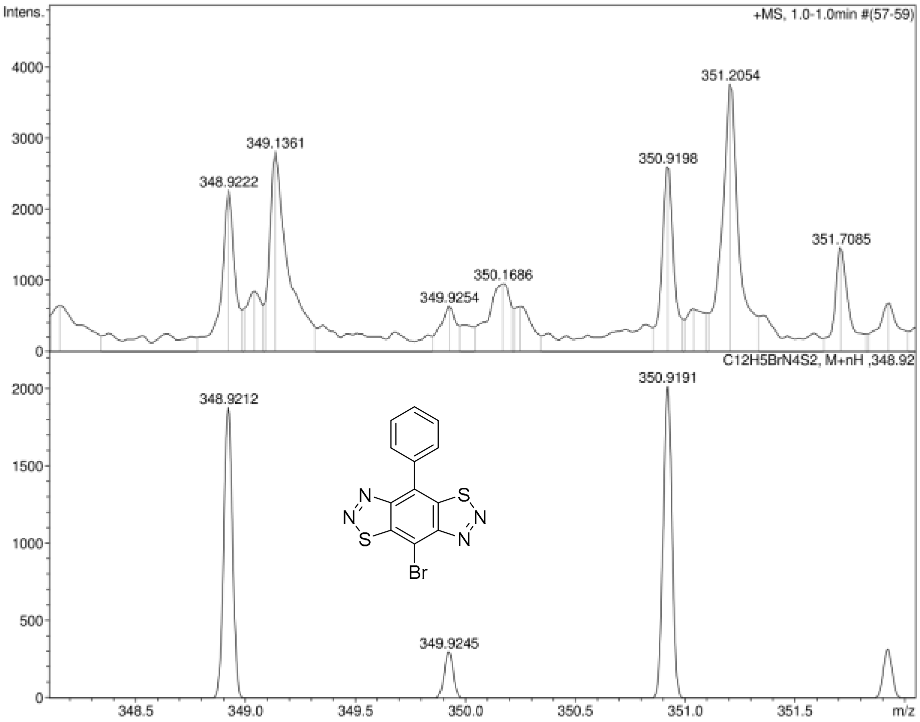

8-Bromo-4-(p-tolyl)benzo[1,2-d:5,4-d']bis([1,2,3]thiadiazole) (18f)

^{1}H NMR (300 MHz)

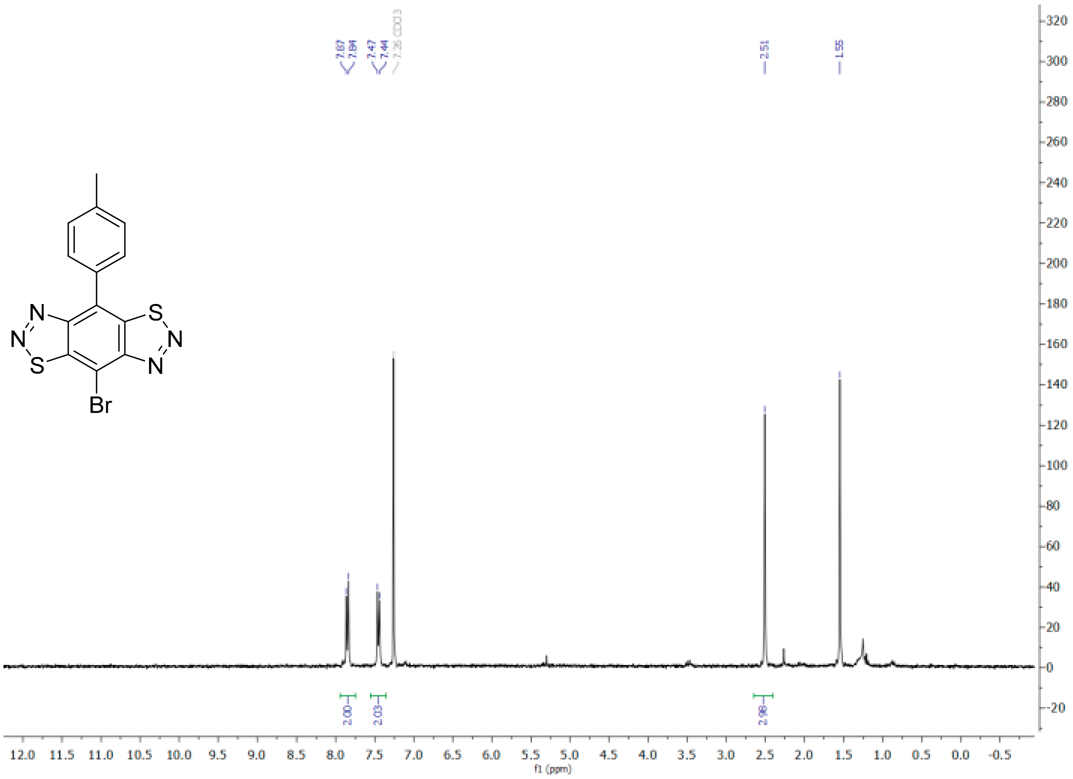

^{13}C NMR(75 MHz)

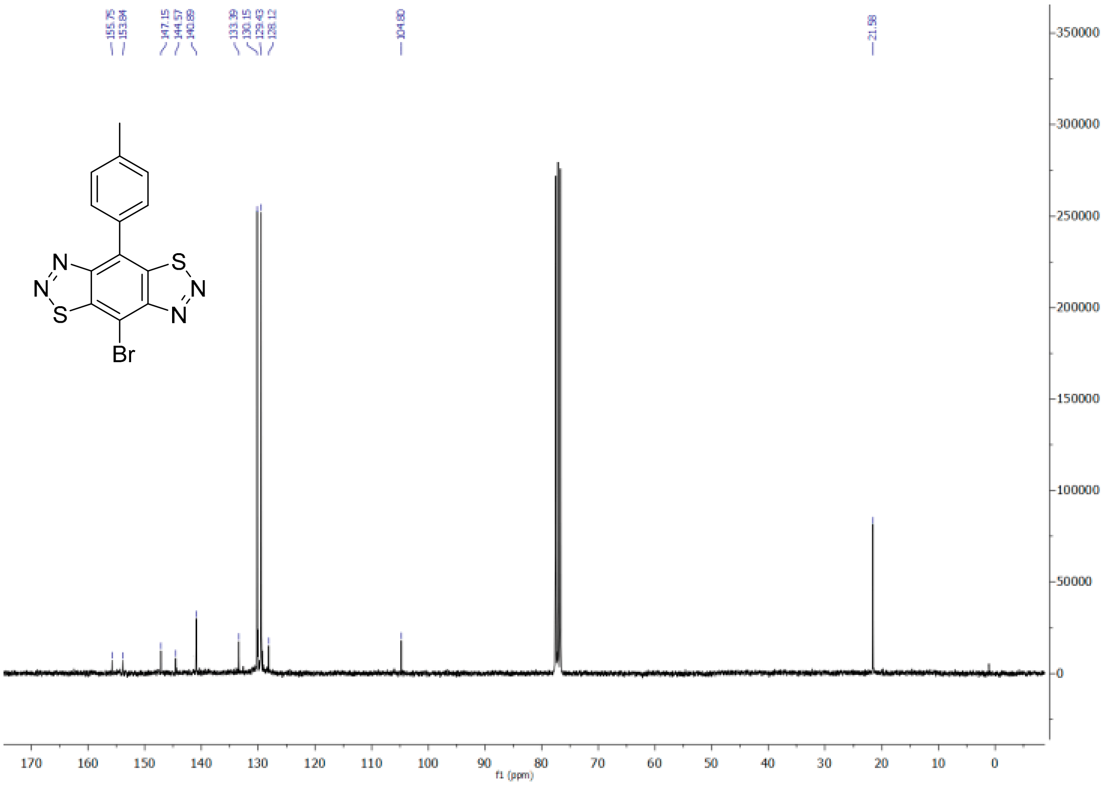
155.75
153.84
147.15
144.57
140.89
133.39
130.15
129.43
128.12
104.80
21.58
N
N
S
S
N
N
Br
350000
300000
250000
200000
150000
100000
50000
0
170
160
150
140
130
120
110
100
90
80
70
60
50
40
30
20
10
0
f1 (ppm)

Display Report

Analysis Info

Analysis Name	D:\Data\Kolotyrkina\2022\Chmovsh\0915007.d	Acquisition Date	15.09.2022 13:26:12
Method	tune_50-1600_pos_15_12.m	Operator	BDAL@DE
Sample Name	/SUSU Tim-553	Instrument / Ser#	micrOTOF 10248
Comment	C13H7BrN4S2 mH 362.9368 calibrant added CH3CN		

Acquisition Parameter

Source Type	ESI	Ion Polarity	Positive	Set Nebulizer	0.4 Bar
Focus	Not active			Set Dry Heater	180 °C
Scan Begin	50 m/z	Set Capillary	4500 V	Set Dry Gas	4.0 l/min
Scan End	1600 m/z	Set End Plate Offset	-500 V	Set Divert Valve	Waste

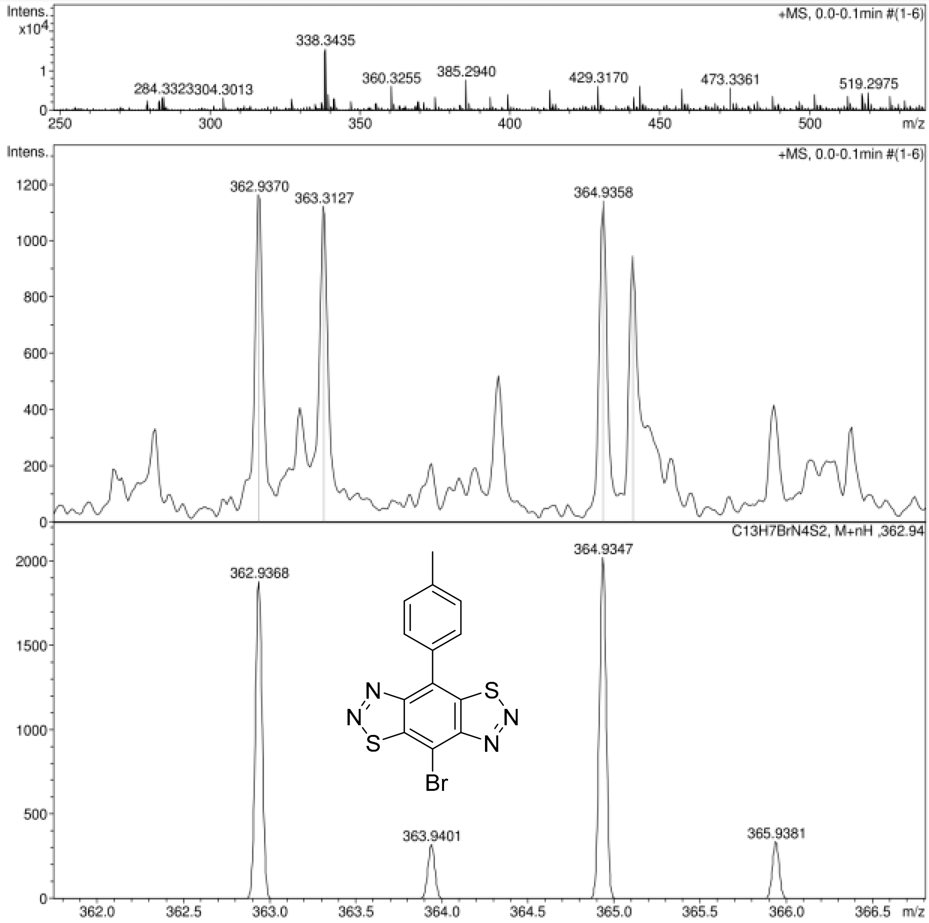

Bruker Compass DataAnalysis 4.0 printed: 15.09.2022 13:31:59 Page 1 of 1

4-Bromo-8-(4-methoxyphenyl)benzo[1,2-d:4,5-d']bis([1,2,3]thiadiazole) (18g)

^{1}H NMR (300 MHz)

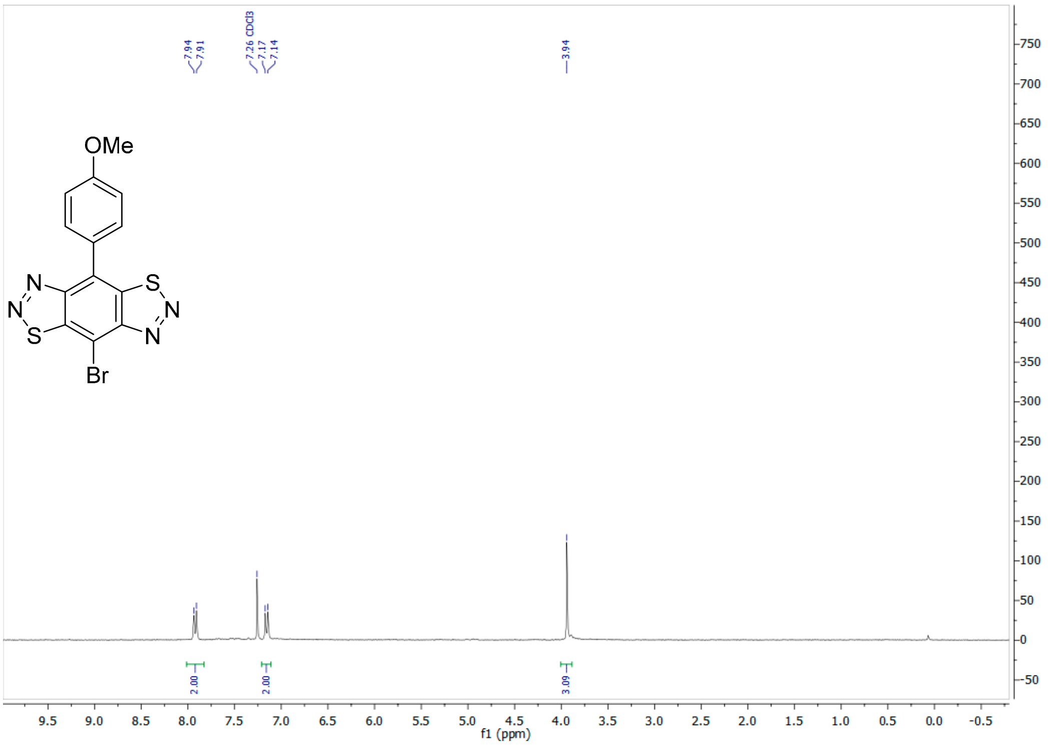

^{13}C NMR(75 MHz)

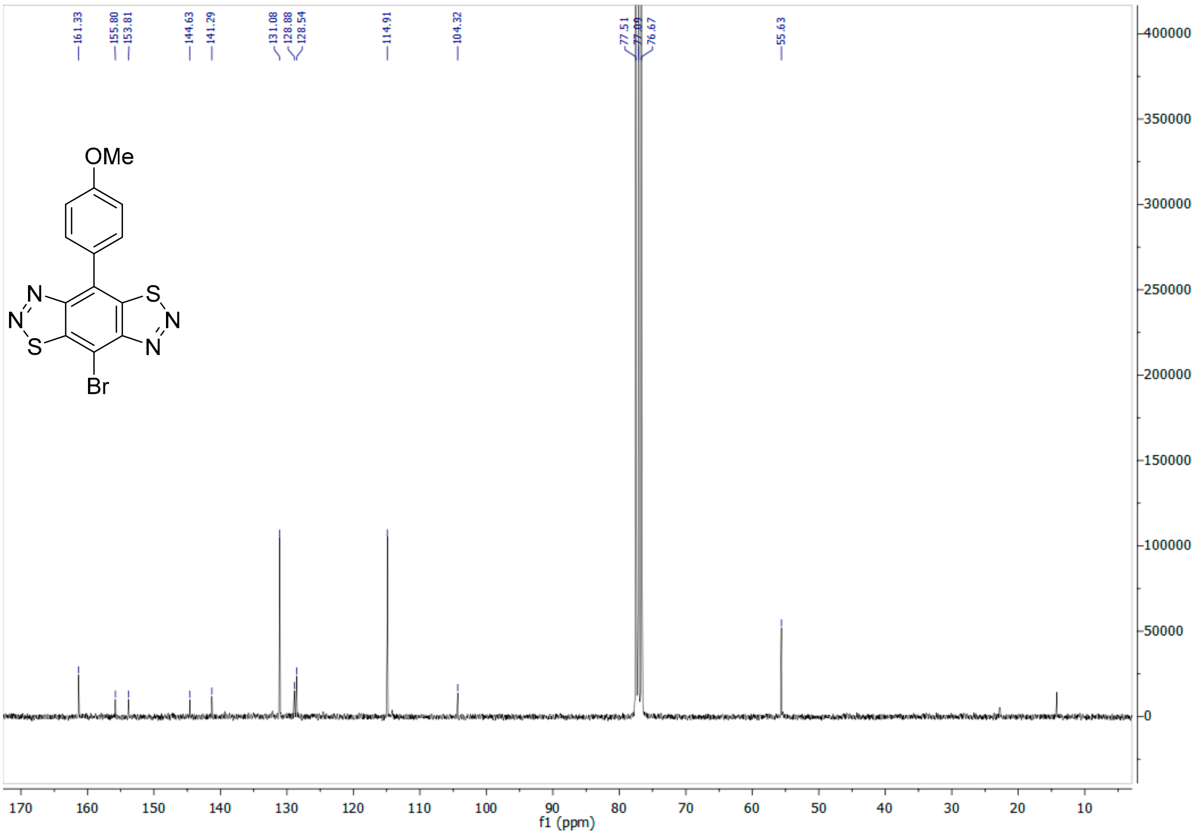

Display Report

Analysis Info

Analysis Name	D:\Data\Kolotyrkina\2022\Chmovsh\1004026.d	Acquisition Date	04.10.2022 19:31:48
Method	tune_50-1600_pos_15_12.m	Operator	BDAL@DE
Sample Name	/SUSU Tim-558	Instrument / Ser#	micrOTOF 10248
Comment	C13H7BrN4OS2 mH 378.9317 clb added CH3CN		

Acquisition Parameter

Source Type	ESI	Ion Polarity	Positive	Set Nebulizer	0.4 Bar
Focus	Not active			Set Dry Heater	180 °C
Scan Begin	50 m/z	Set Capillary	4500 V	Set Dry Gas	4.0 l/min
Scan End	1600 m/z	Set End Plate Offset	-500 V	Set Divert Valve	Waste

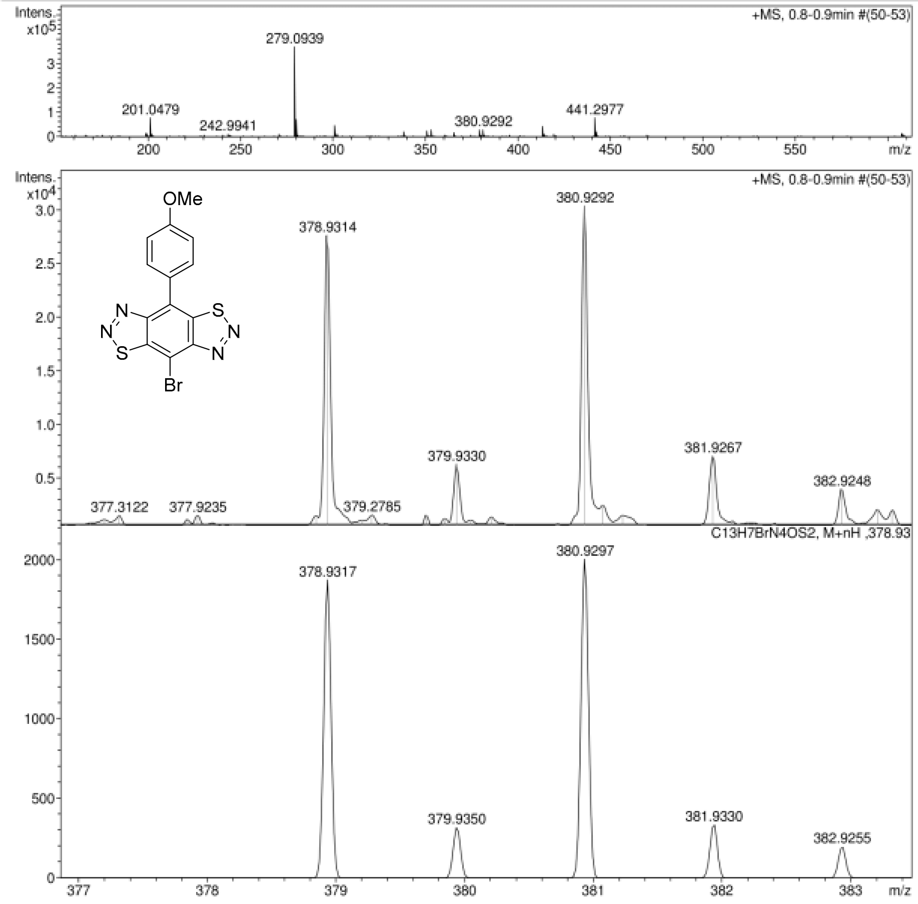

4-(8-Bromobenzo[1,2-d:4,5-d']bis([1,2,3]thiadiazole)-4-yl)-N,N-diphenylaniline (18h)

^{1}H NMR (300 MHz)

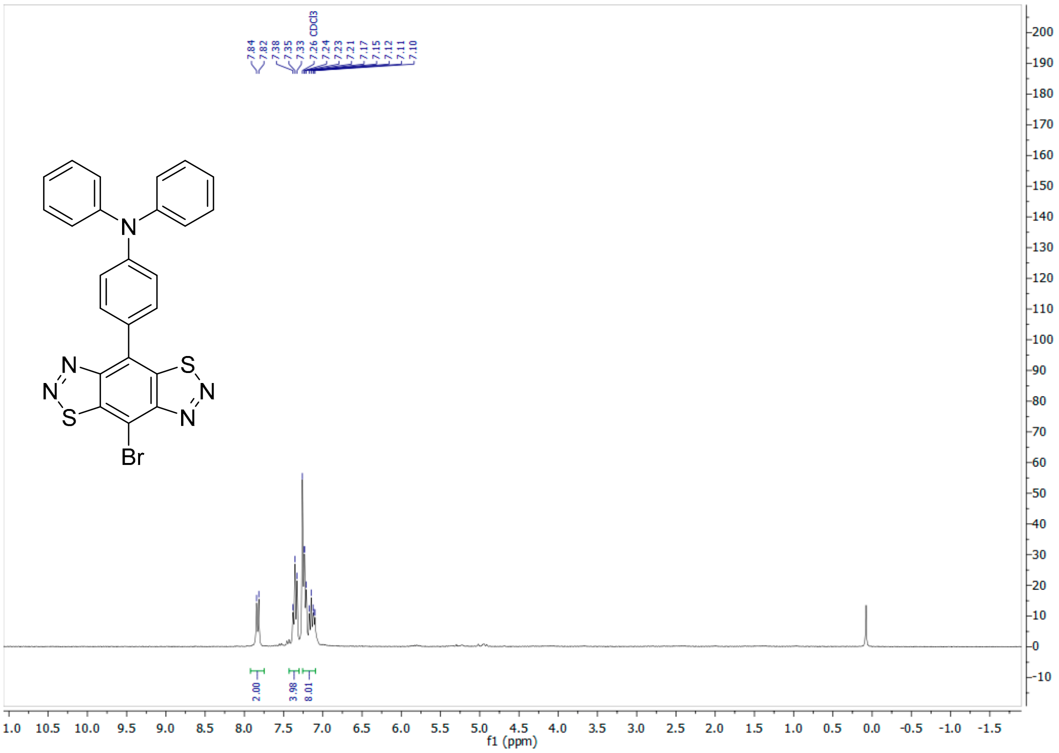

^{13}C NMR(75 MHz)

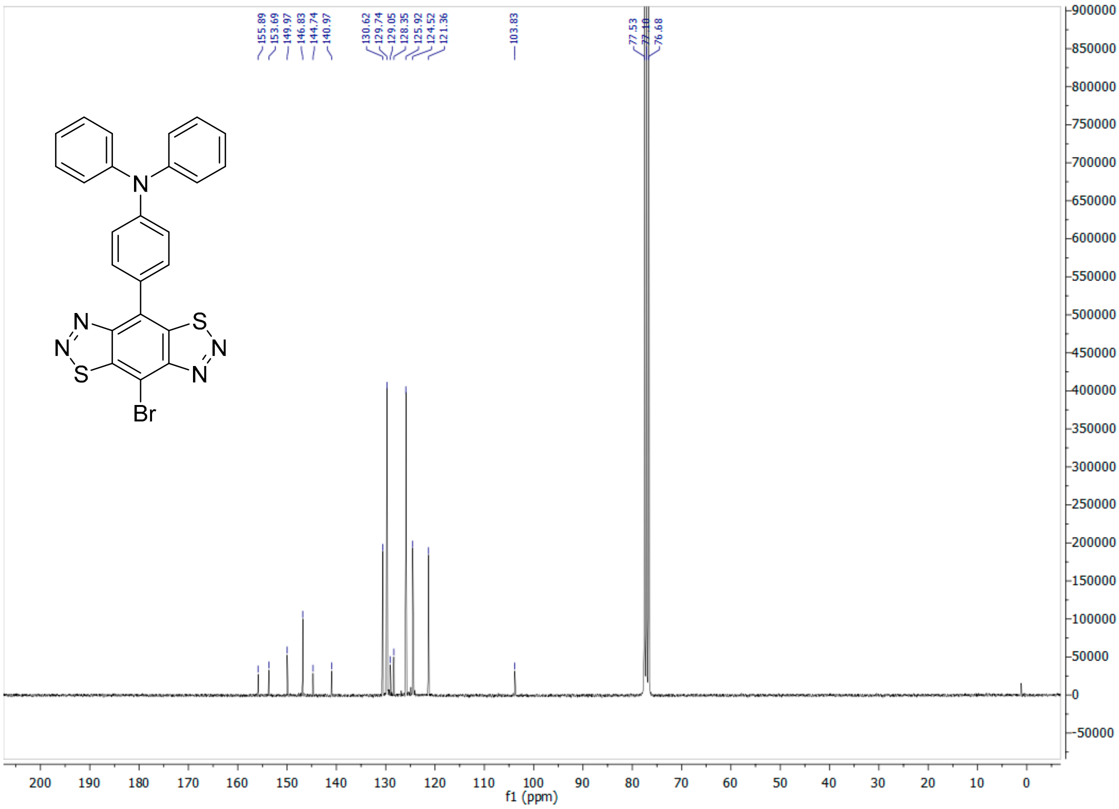

Display Report

Analysis Info

Analysis Name	D:\Data\Kolotyrkina\2022\Chmovsh\0728022.d	Acquisition Date	28.07.2022 15:44:25
Method	tune_wide.m	Operator	BDAL@DE
Sample Name	/SUSU Tim-546	Instrument / Ser#	maXis 43
Comment	C24H14BrN5S2 mH 515.9946 clb added CH3CN		

Acquisition Parameter

Source Type	ESI	Ion Polarity	Positive	Set Nebulizer	0.5 Bar
Focus	Active			Set Dry Heater	180 °C
Scan Begin	50 m/z	Set Capillary	4500 V	Set Dry Gas	4.0 l/min
Scan End	3000 m/z	Set End Plate Offset	-500 V	Set Divert Valve	Waste

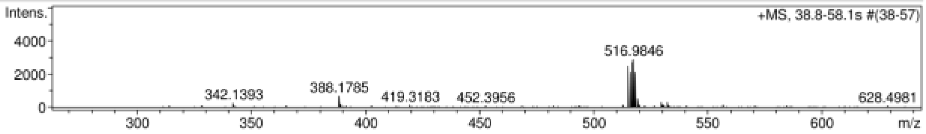

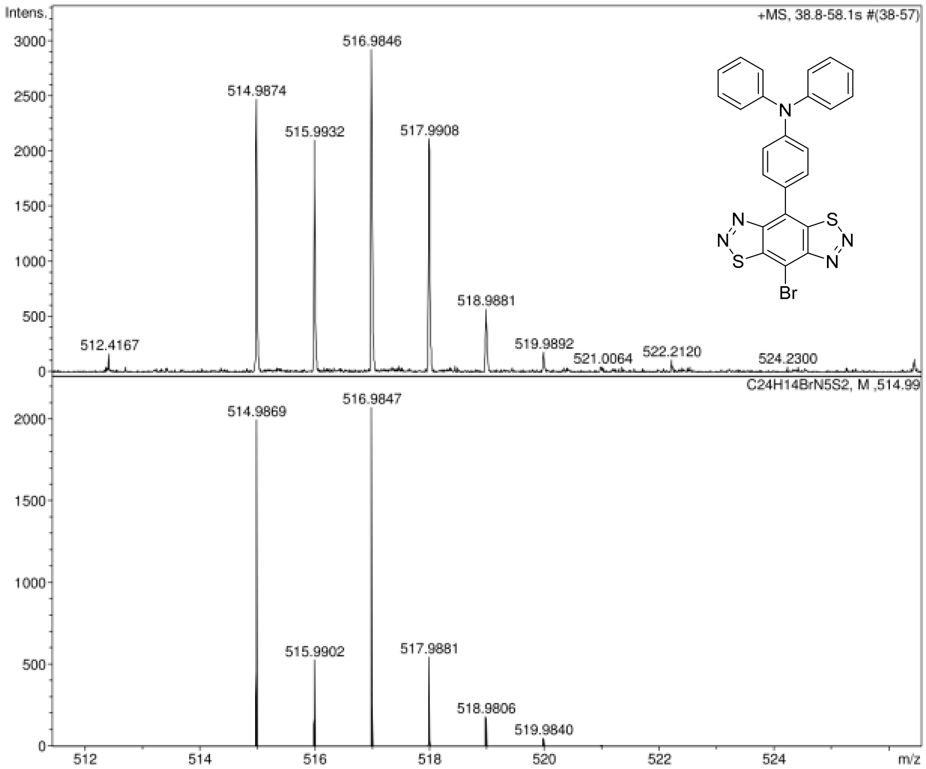

4,8-Di(thiophen-2-yl)benzo[1,2-d:4,5-d']bis([1,2,3]thiadiazole) (19a)

^{1}H NMR (300 MHz)

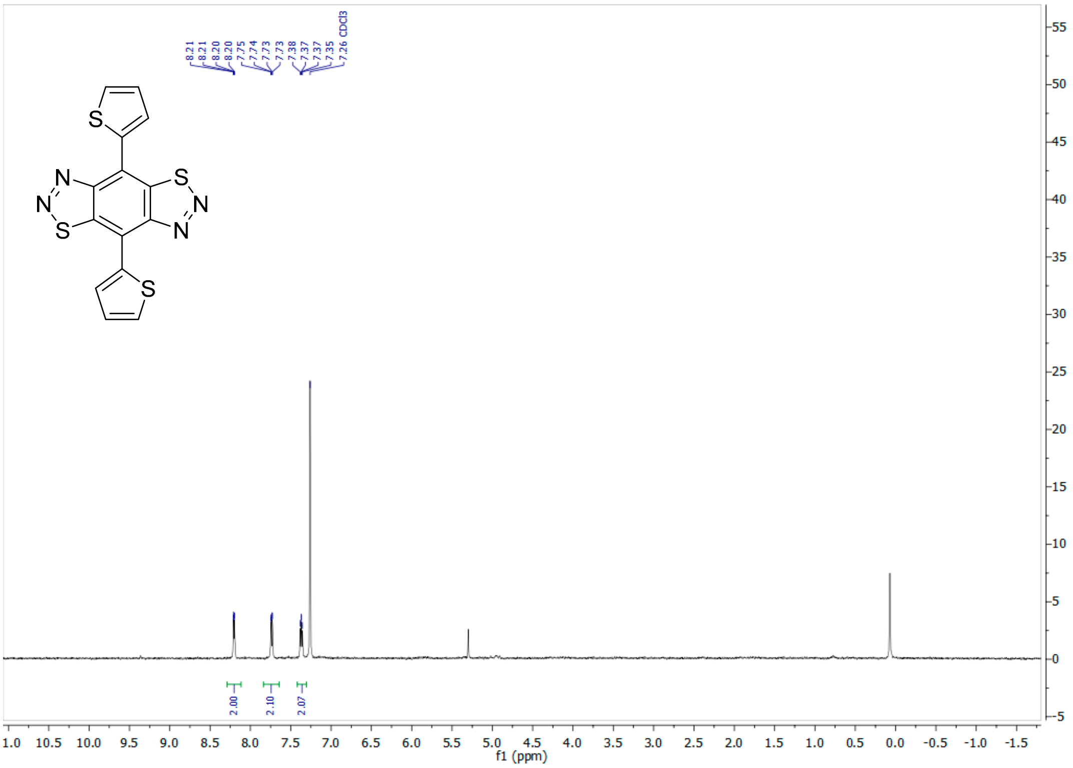

^{13}C NMR(75 MHz)

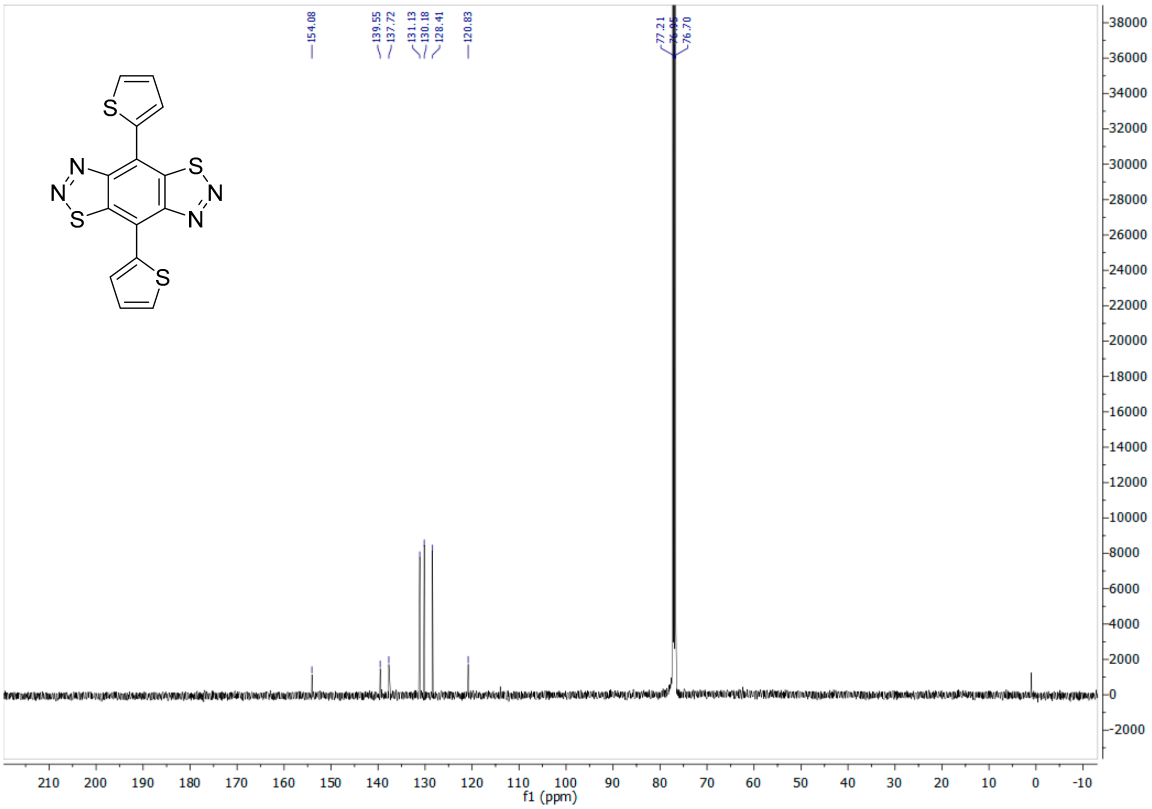

154.08
139.55
137.72
131.13
130.18
128.41
120.83
77.21
76.95
76.70
f1 (ppm)

Display Report

Analysis Info

Analysis Name	D:\Data\Kolotyrkina\2022\Chmovsh\0413003.d	Acquisition Date	13.04.2022 10:32:53
Method	tune_50-1600_pos_15_12.m	Operator	BDAL@DE
Sample Name	/SUSU Tim-519	Instrument / Ser#	micrOTOF 10248
Comment	C14H6N4S4 mH 358.9548 clb added CH3CN		

Acquisition Parameter

Source Type	ESI	Ion Polarity	Positive	Set Nebulizer	0.4 Bar
Focus	Not active			Set Dry Heater	180 °C
Scan Begin	50 m/z	Set Capillary	4500 V	Set Dry Gas	4.0 l/min
Scan End	1600 m/z	Set End Plate Offset	-500 V	Set Divert Valve	Waste

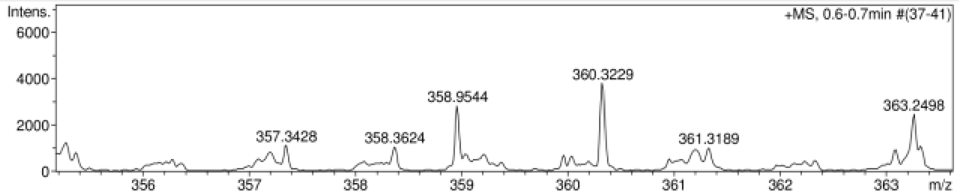

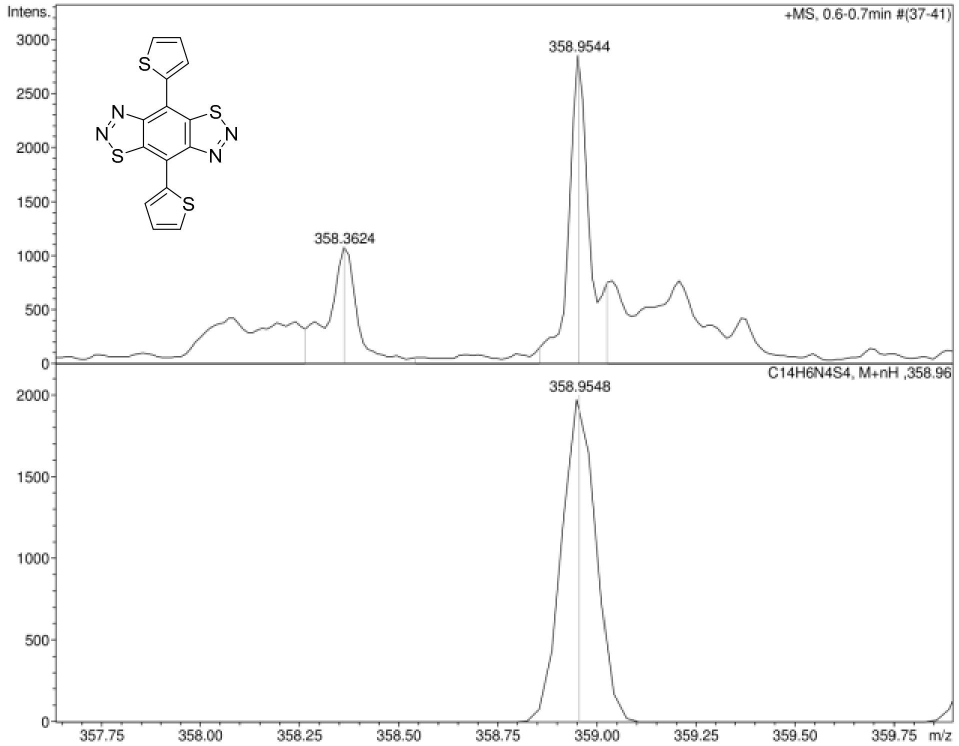

4,8-Bis(4-hexylthiophen-2-yl)benzo[1,2-d:4,5-d']bis([1,2,3]thiadiazole) (19b)

^{1}H NMR (300 MHz)

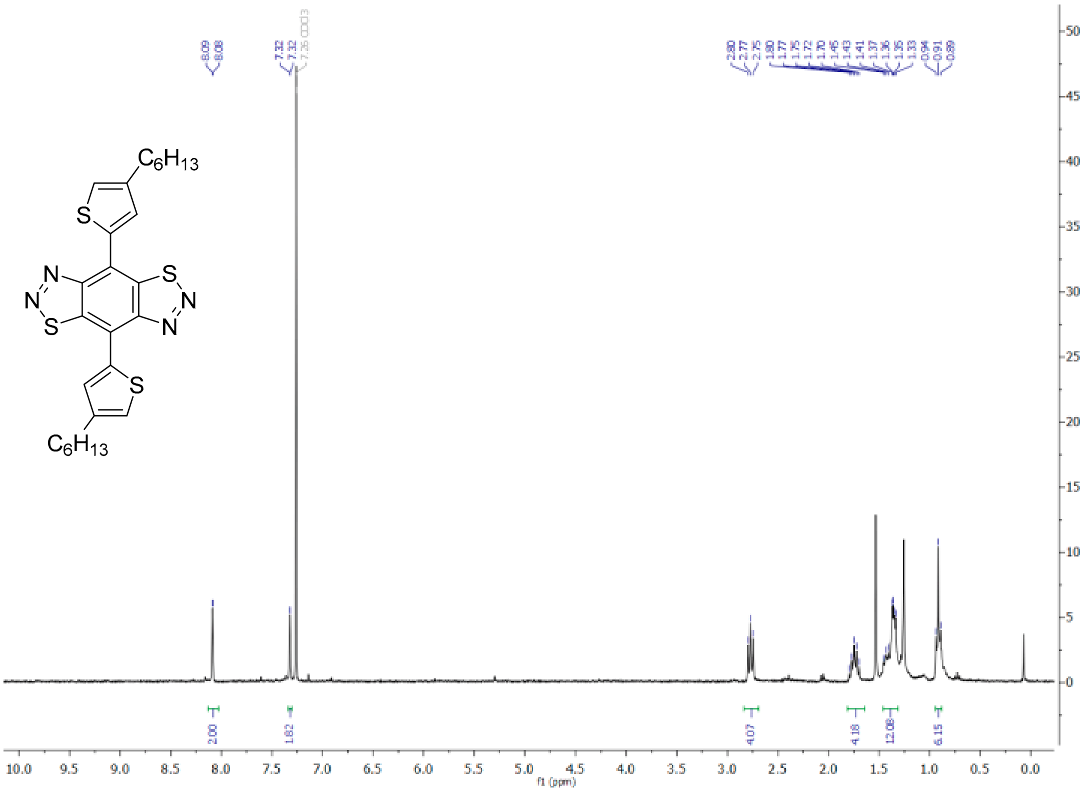

^{13}C NMR(75 MHz)

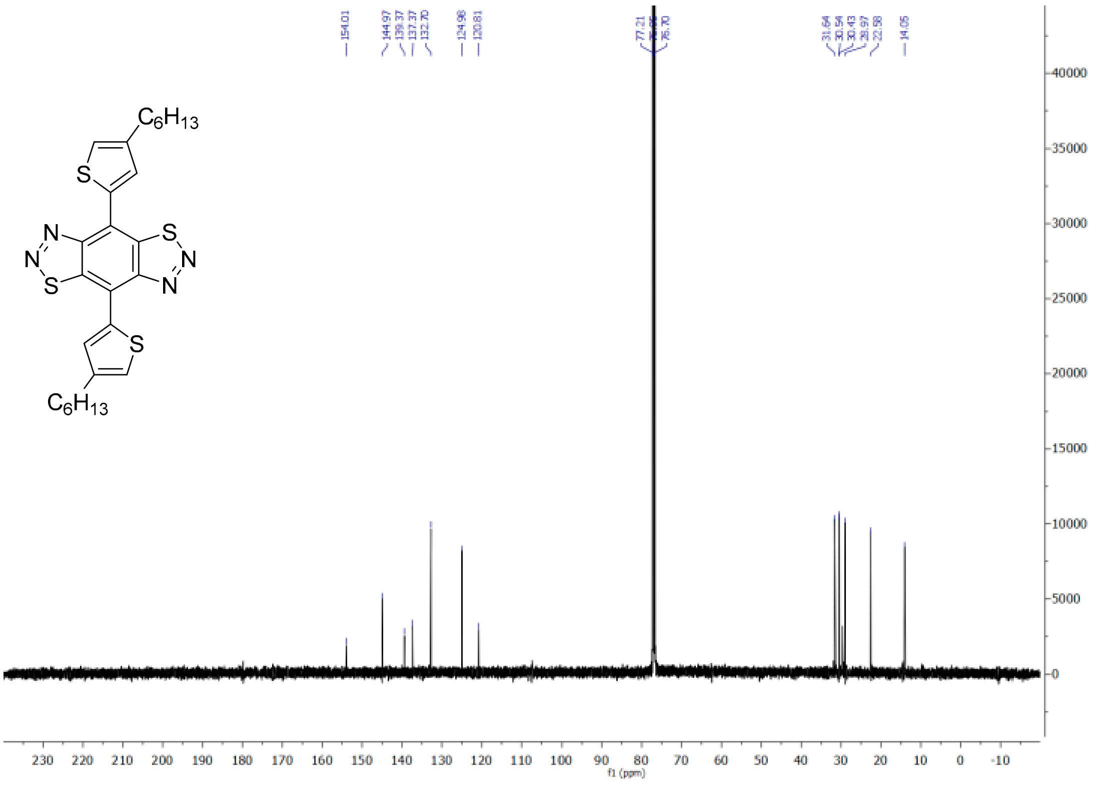

Display Report

Analysis Info

Analysis Name	D:\Data\Kolotyrkina\2022\Chmovsh\0324040.d	Acquisition Date	24.03.2022 19:08:23
Method	tune_50-1600_pos_15_12.m	Operator	BDAL@DE
Sample Name	/SUSU Tim-517	Instrument / Ser#	micrOTOF 10248
Comment	C26H30N4S4 mH 527.1426 calibrant added CN3CN		

Acquisition Parameter

Source Type	ESI	Ion Polarity	Positive	Set Nebulizer	0.4 Bar
Focus	Not active			Set Dry Heater	180 °C
Scan Begin	50 m/z	Set Capillary	4500 V	Set Dry Gas	4.0 l/min
Scan End	1600 m/z	Set End Plate Offset	-500 V	Set Divert Valve	Waste

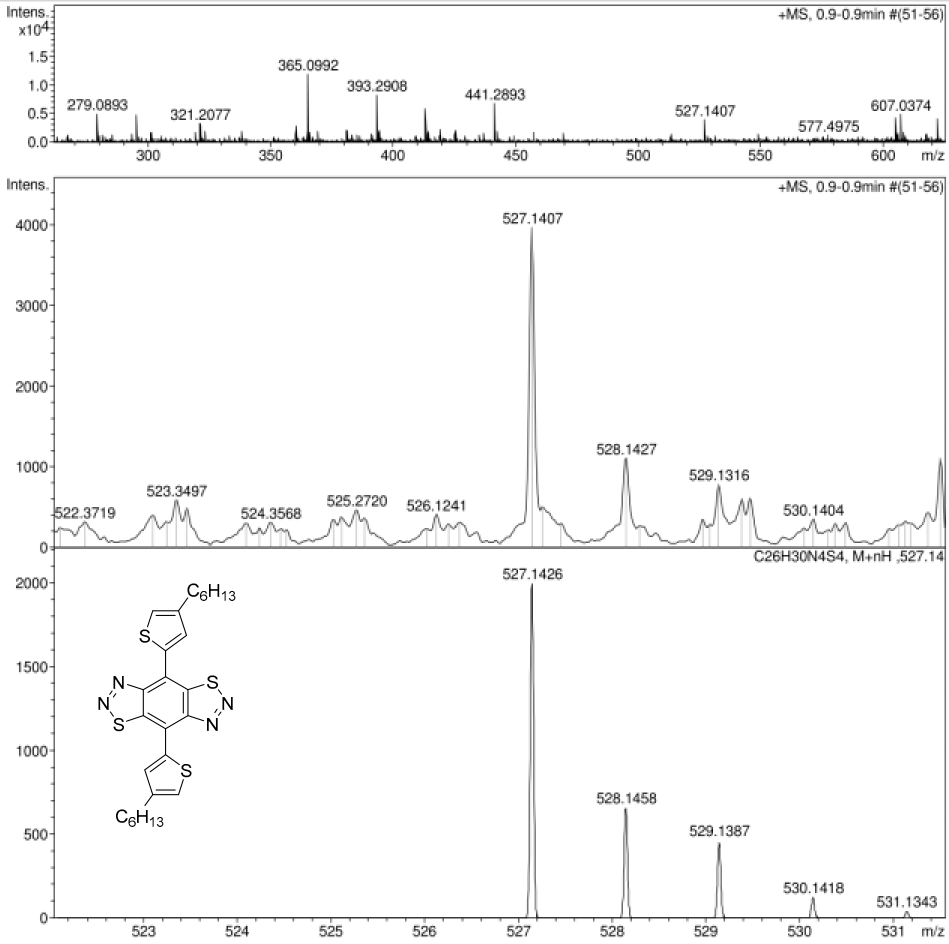

4,8-Di([2,2'-bithiophen]-5-yl)benzo[1,2-d:4,5-d']bis([1,2,3]thiadiazole) (19c)

^{1}H NMR (300 MHz)

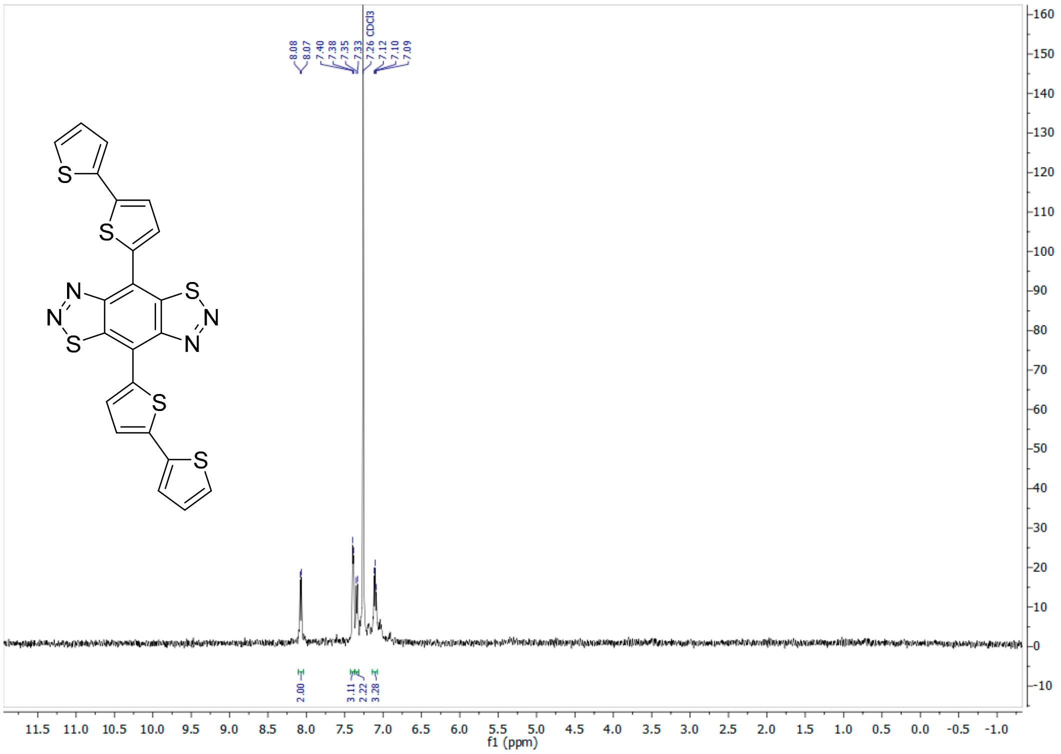

^{13}C NMR(75 MHz)

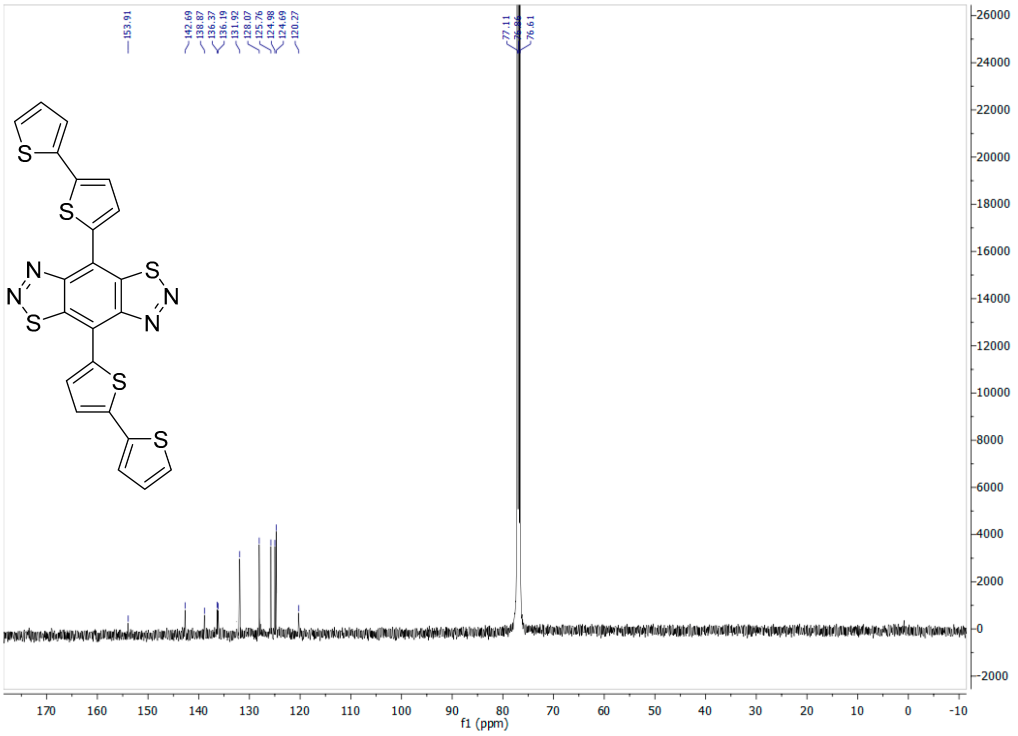

Display Report

Analysis Info

Analysis Name	D:\Data\Kolotyrkina\2022\Chmovsh\0920003.d
Method	tune_50-1600_pos_15_12.m
Sample Name	/SUSU Tim-555
Comment	C22H10N4S6 mH 522.9302 calibrant added CH3CN

Acquisition Date	20.09.2022 11:35:18
Operator	BDAL@DE
Instrument / Ser#	micrOTOF 10248

Acquisition Parameter

Source Type	ESI	Ion Polarity	Positive	Set Nebulizer	0.4 Bar
Focus	Not active			Set Dry Heater	180 °C
Scan Begin	50 m/z	Set Capillary	4500 V	Set Dry Gas	4.0 l/min
Scan End	1600 m/z	Set End Plate Offset	-500 V	Set Divert Valve	Waste

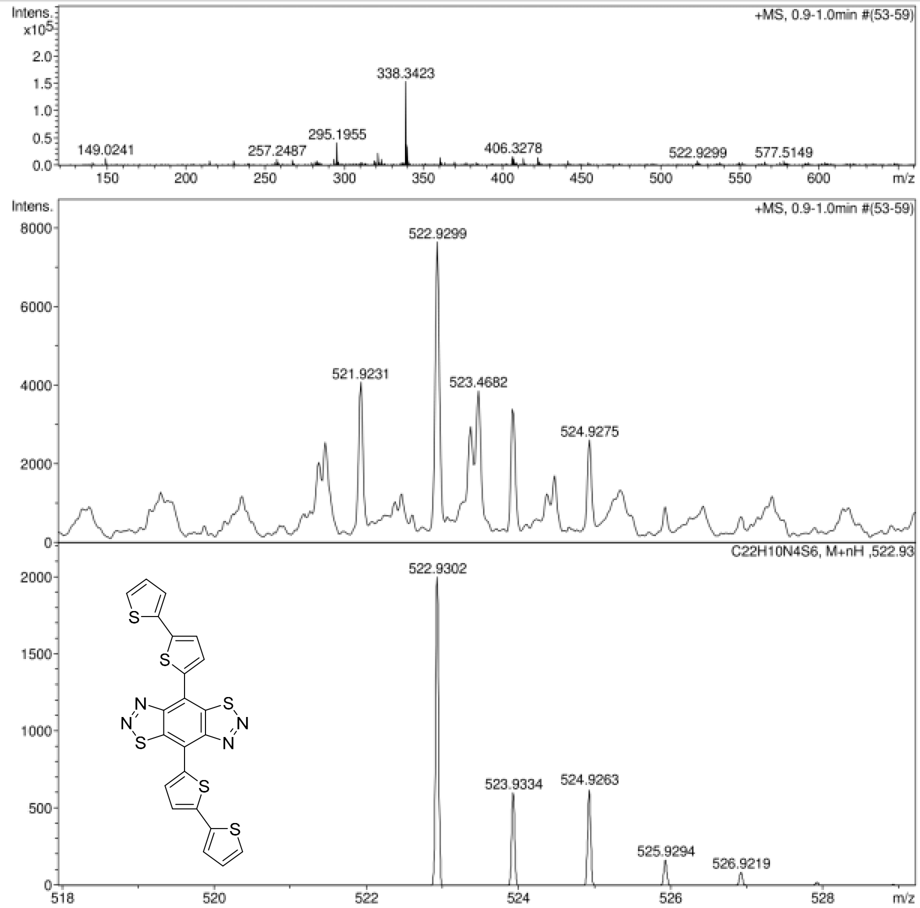

4,8-Bis(5-(2-ethylhexyl)thiophen-2-yl)benzo[1,2-d:4,5-d']bis([1,2,3]thiadiazole) (19d)

^{1}H NMR (300 MHz)

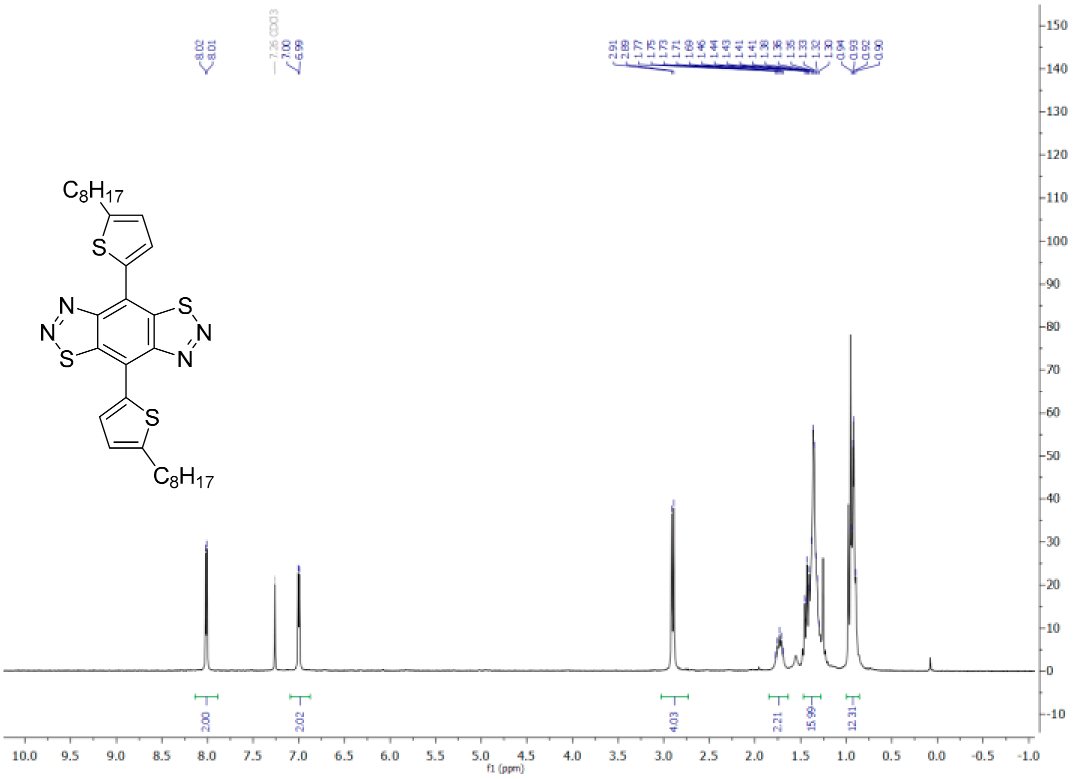

^{13}C NMR(75 MHz)

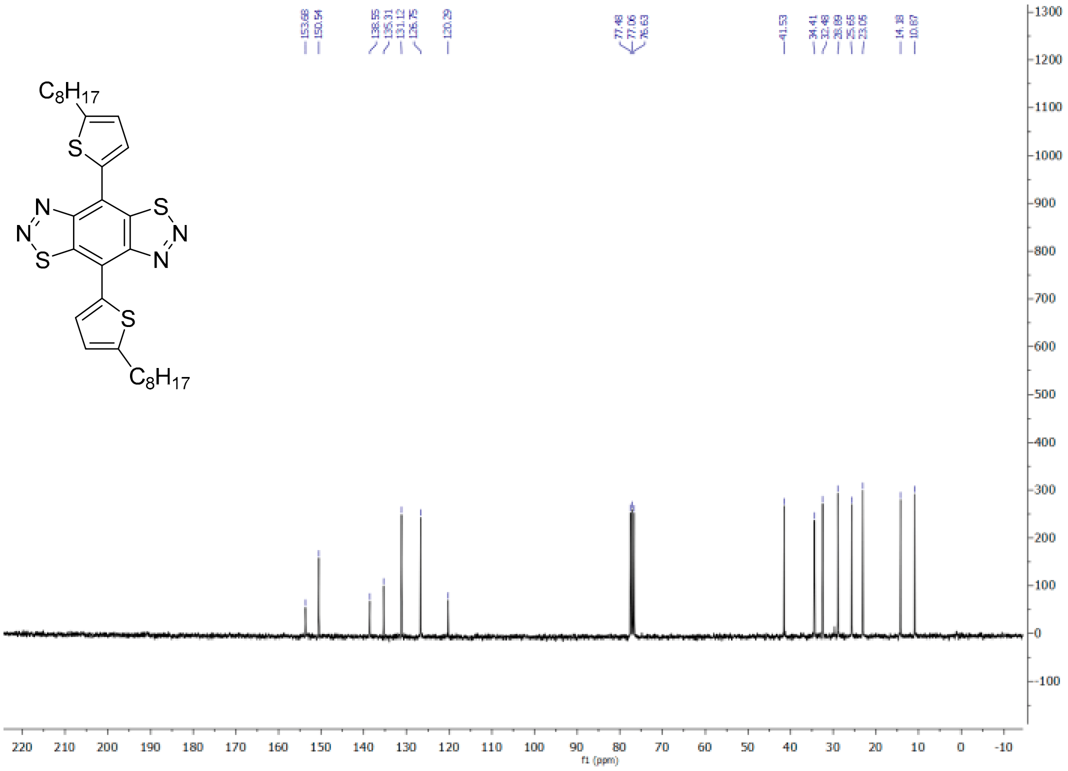

C8H17
S
N
N
S
S
N
N
S
C8H17
153.68
150.54
138.55
135.31
131.12
126.75
120.29
77.48
77.06
76.63
41.53
34.41
32.48
28.89
25.65
23.05
14.18
10.67
f1 (ppm)

Display Report

Analysis Info

Analysis Name	D:\Data\Chizhov\Rakitin\Chmovzh\tim-507_&clb.d	Acquisition Date	01.02.2022 16:28:52
Method	tune_wide.m	Operator	BDAL@DE
Sample Name	/SUSU Tim-507	Instrument / Ser#	micrOTOF 10248
Comment	CH3CN 100 %, dil. 200, calibrant added		

Acquisition Parameter

Source Type	ESI	Ion Polarity	Positive	Set Nebulizer	0.4 Bar
Focus	Not active			Set Dry Heater	180 °C
Scan Begin	50 m/z	Set Capillary	4500 V	Set Dry Gas	4.0 l/min
Scan End	3000 m/z	Set End Plate Offset	-500 V	Set Divert Valve	Waste

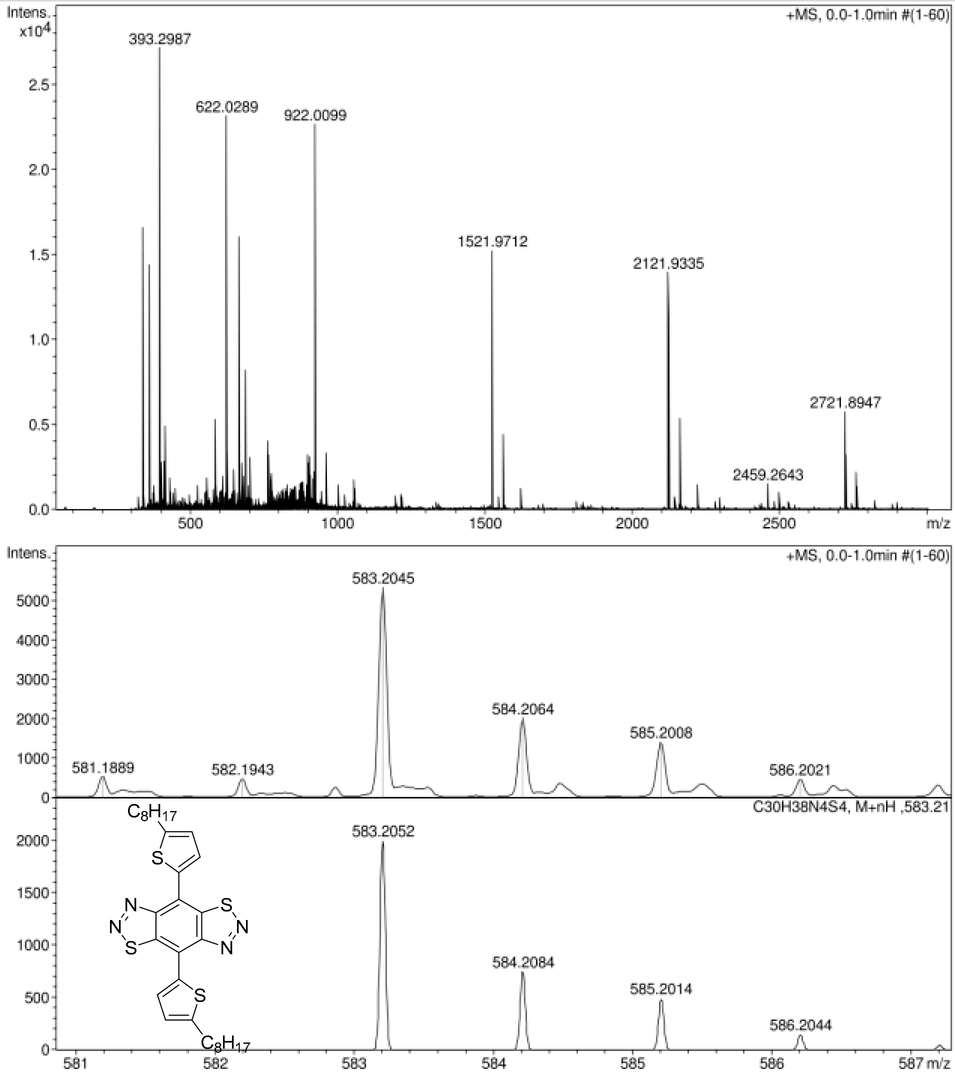

Bruker Compass DataAnalysis 4.0 printed: 01.02.2022 16:34:00 Page 1 of 1

4,8-Diphenylbenzo[1,2-d:4,5-d']bis([1,2,3]thiadiazole) (19e)

^{1}H NMR (300 MHz)

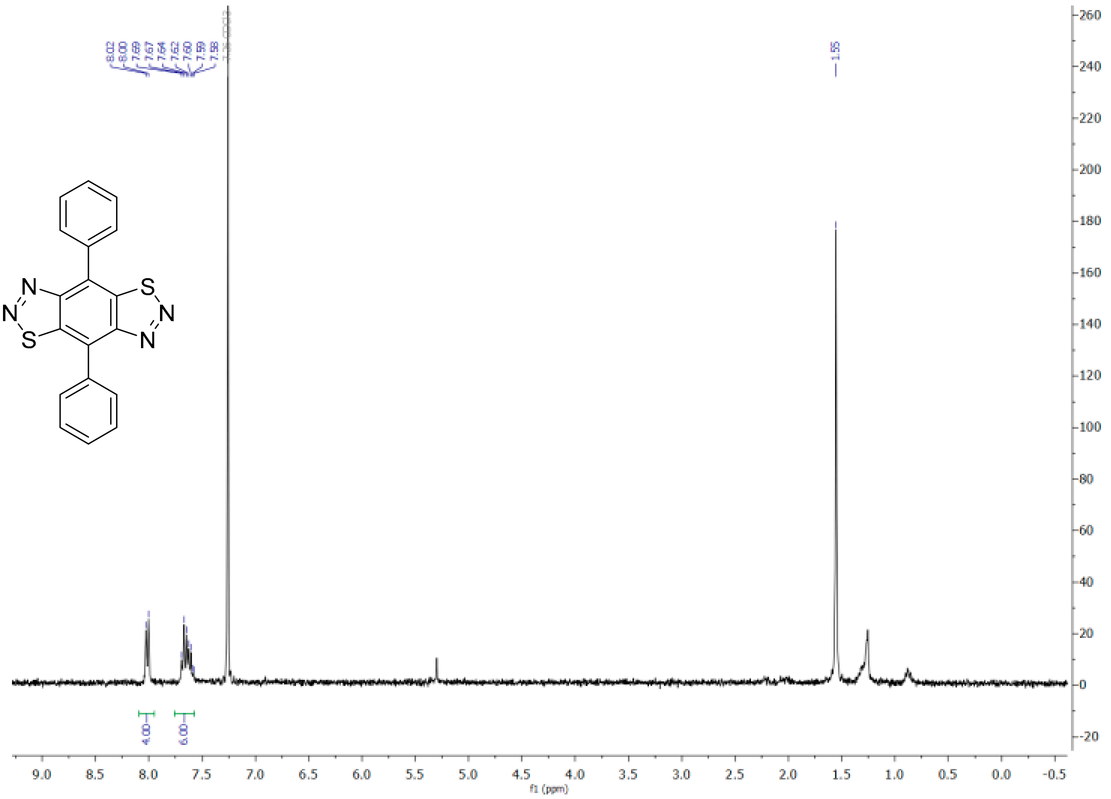

^{13}C NMR(75 MHz)

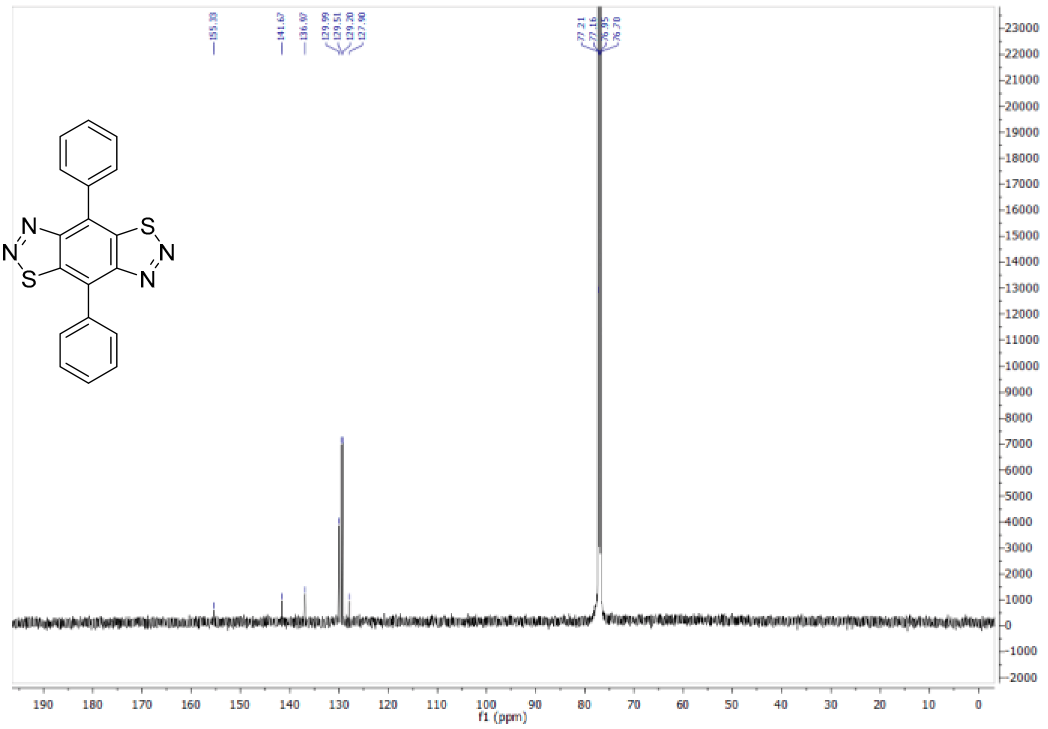

]

Display Report

Analysis Info

Analysis Name	D:\Data\Kolotyrkina\2022\Chmovsh\0608028.d	Acquisition Date	08.06.2022 16:45:25
Method	tune_50-1600_pos_15_12.m	Operator	BDAL@DE
Sample Name	/SUSU Tim-533	Instrument / Ser#	micrOTOF 10248
Comment	C18H10N4S2 mH 347.0419 clb added CH3CN		

Acquisition Parameter

Source Type	ESI	Ion Polarity	Positive	Set Nebulizer	0.4 Bar
Focus	Not active			Set Dry Heater	180 °C
Scan Begin	50 m/z	Set Capillary	4500 V	Set Dry Gas	4.0 l/min
Scan End	1600 m/z	Set End Plate Offset	-500 V	Set Divert Valve	Waste

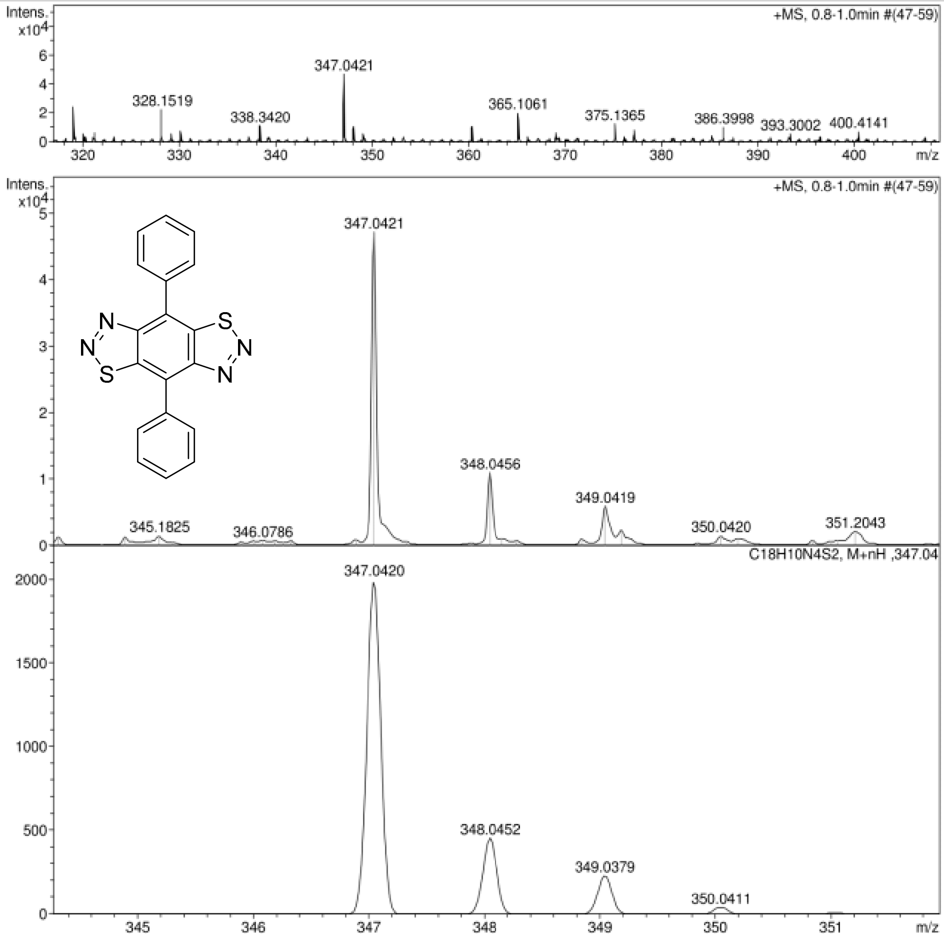

4,8-Di-p-tolylbenzo[1,2-d:4,5-d']bis([1,2,3]thiadiazole) (19f)

^{1}H NMR (300 MHz)

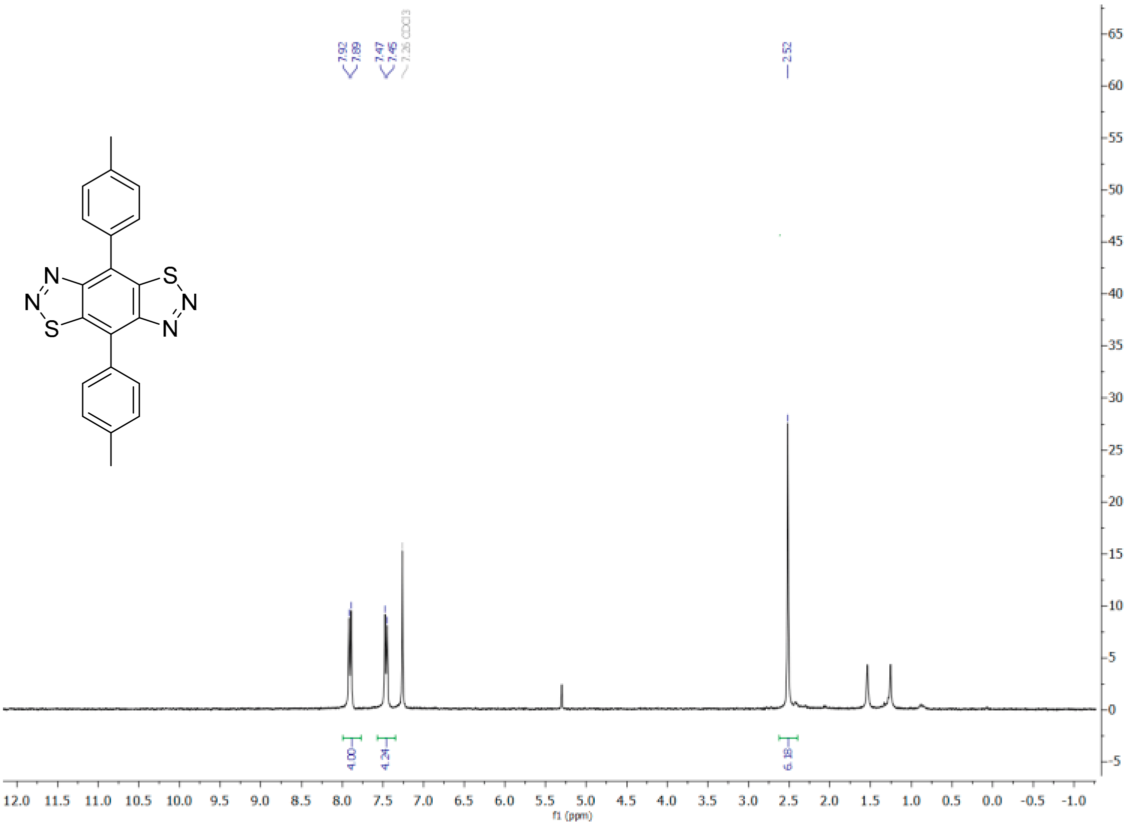

^{13}C NMR(75 MHz)

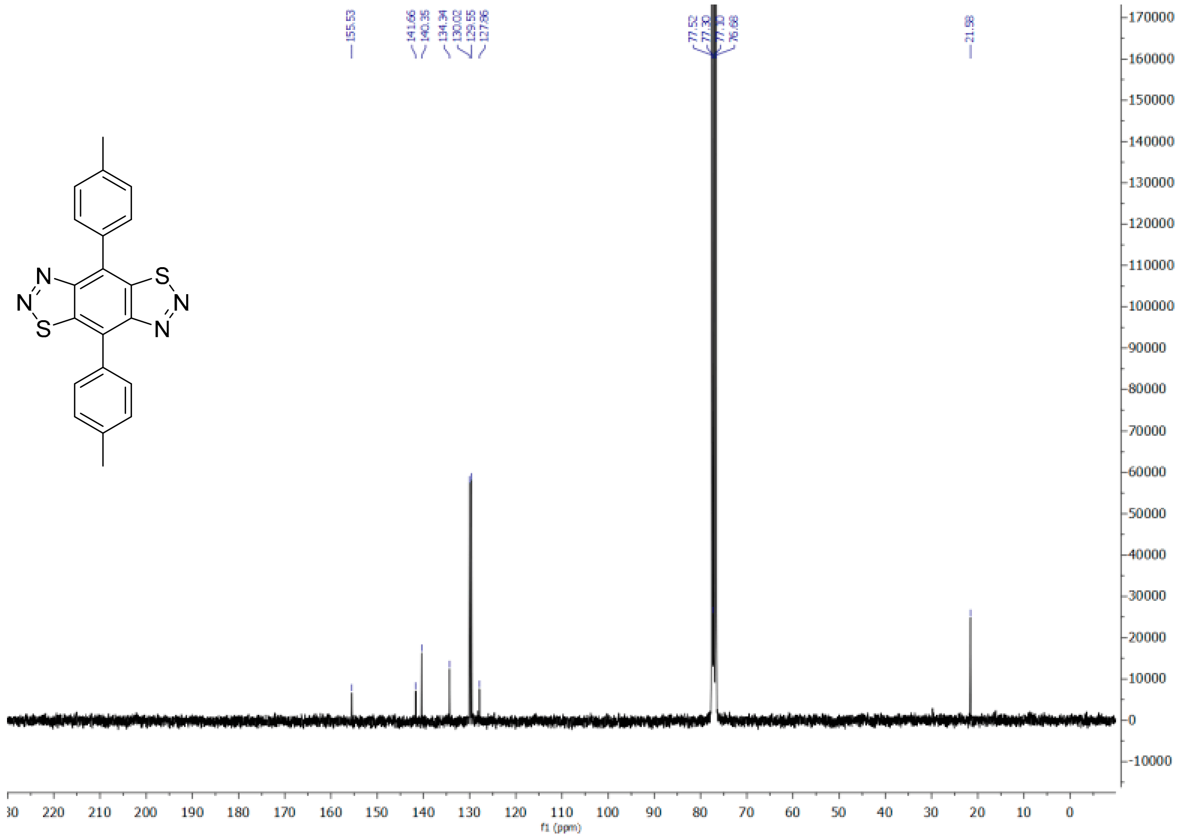

Display Report

Analysis Info

Analysis Name	D:\Data\Kolotyrkina\2022\Chmovsh\0330001.d	Acquisition Date	30.03.2022 10:00:29
Method	tune_50-1600_pos_15_12.m	Operator	BDAL@DE
Sample Name	/SUSU Tim-518	Instrument / Ser#	micrOTOF 10248
Comment	C20H14N4S2 mH 375.0732 calibrant added CH3CN		

Acquisition Parameter

Source Type	ESI	Ion Polarity	Positive	Set Nebulizer	0.4 Bar
Focus	Not active			Set Dry Heater	180 °C
Scan Begin	50 m/z	Set Capillary	4500 V	Set Dry Gas	4.0 l/min
Scan End	1600 m/z	Set End Plate Offset	-500 V	Set Divert Valve	Waste

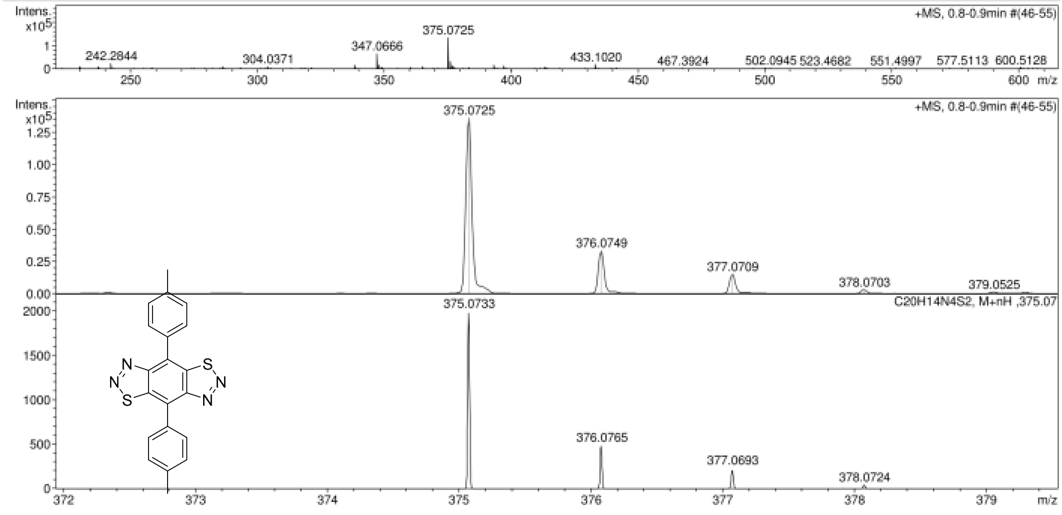

Bruker Compass DataAnalysis 4.0 printed: 30.03.2022 10:03:42 Page 1 of 1

4,8-Bis(4-methoxyphenyl)benzo[1,2-d:4,5-d']bis([1,2,3]thiadiazole) (19g)

^{1}H NMR (300 MHz)

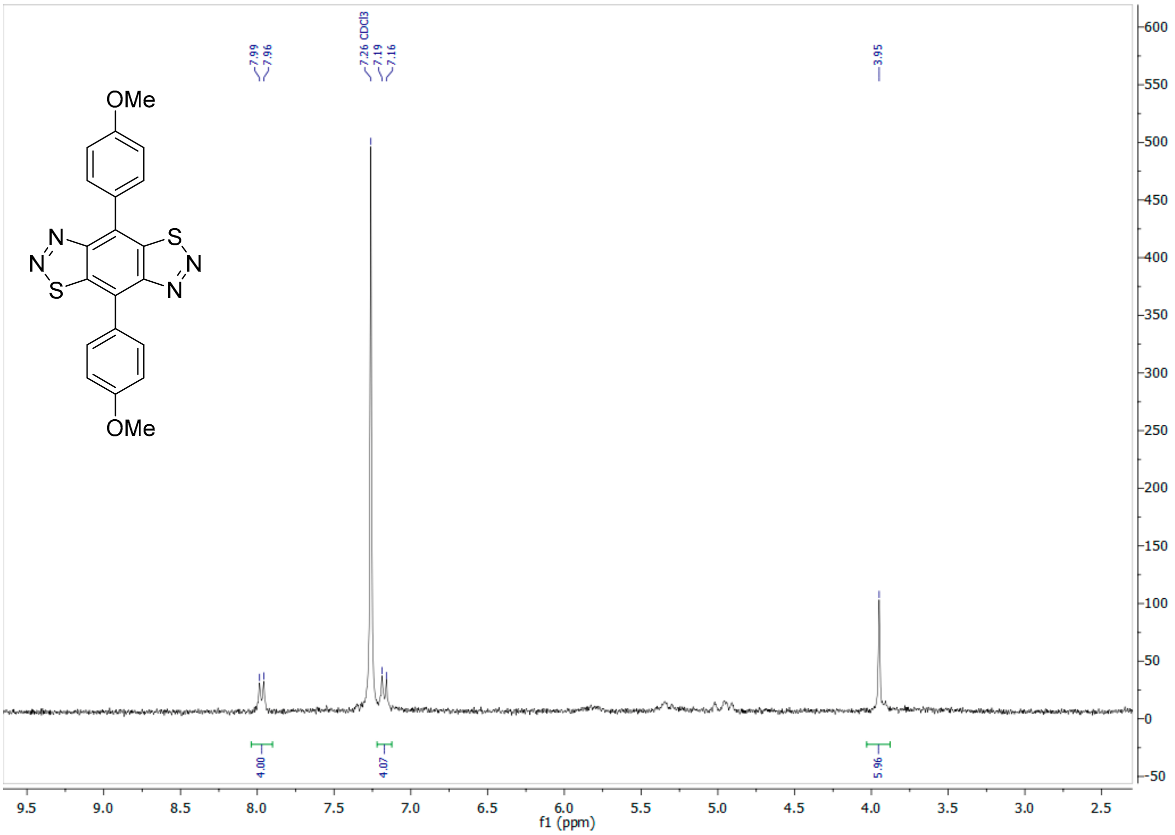

Display Report

Analysis Info

Analysis Name	D:\Data\Chizhov\Rakitin\Chmovzh\tim-534_&clb.d	Acquisition Date	28.06.2022 17:07:17
Method	tune_wide.m	Operator	BDAL@DE
Sample Name	/SUSU Tim-534	Instrument / Ser#	micrOTOF 10248
Comment	CH3CN 100 %, dil. 20, calibrant added		

Acquisition Parameter

Source Type	ESI	Ion Polarity	Positive	Set Nebulizer	0.4 Bar
Focus	Not active			Set Dry Heater	180 °C
Scan Begin	50 m/z	Set Capillary	4500 V	Set Dry Gas	4.0 l/min
Scan End	3000 m/z	Set End Plate Offset	-500 V	Set Divert Valve	Waste

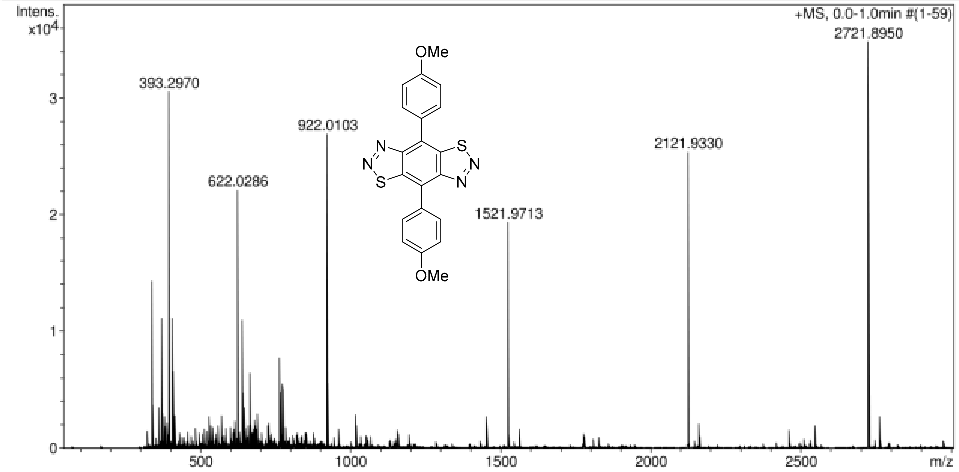

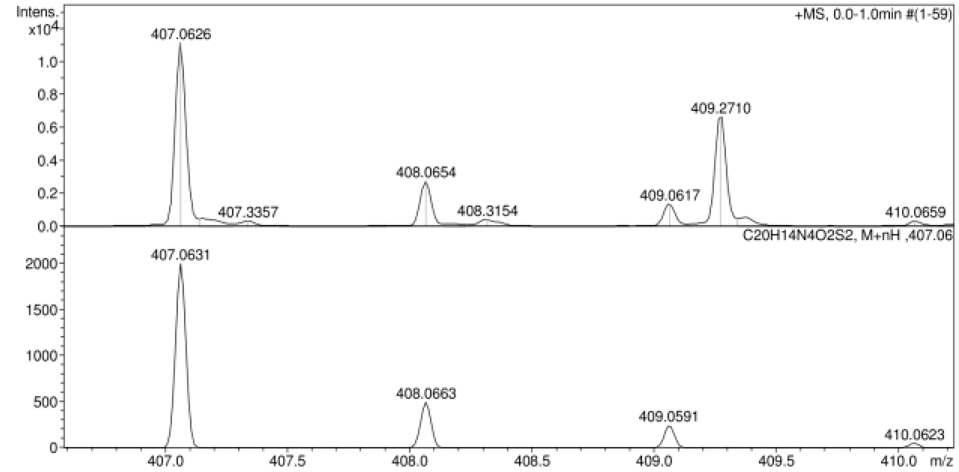

4,4'-(Benzo[1,2-d:4,5-d']bis([1,2,3]thiadiazole)-4,8-diyl)bis(N,N-diphenylaniline) (19h)

^{1}H NMR (300 MHz)

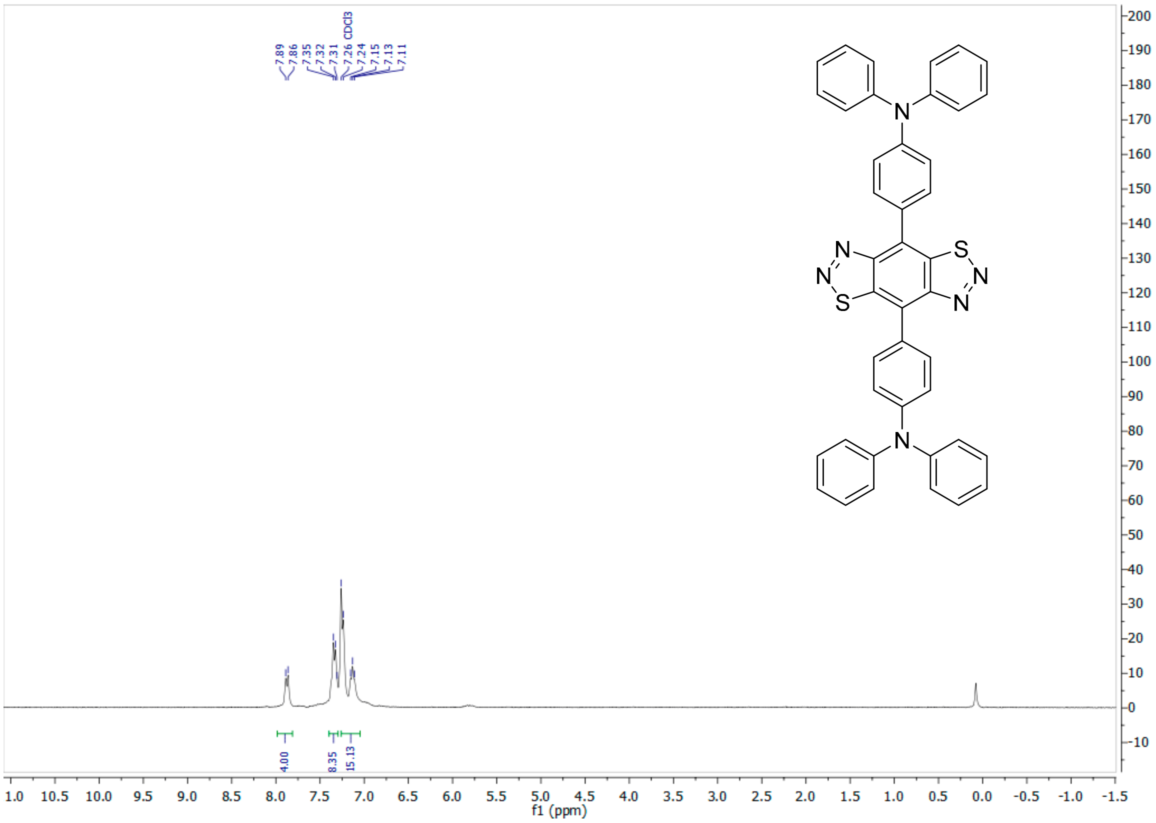

^{13}C NMR(75 MHz)

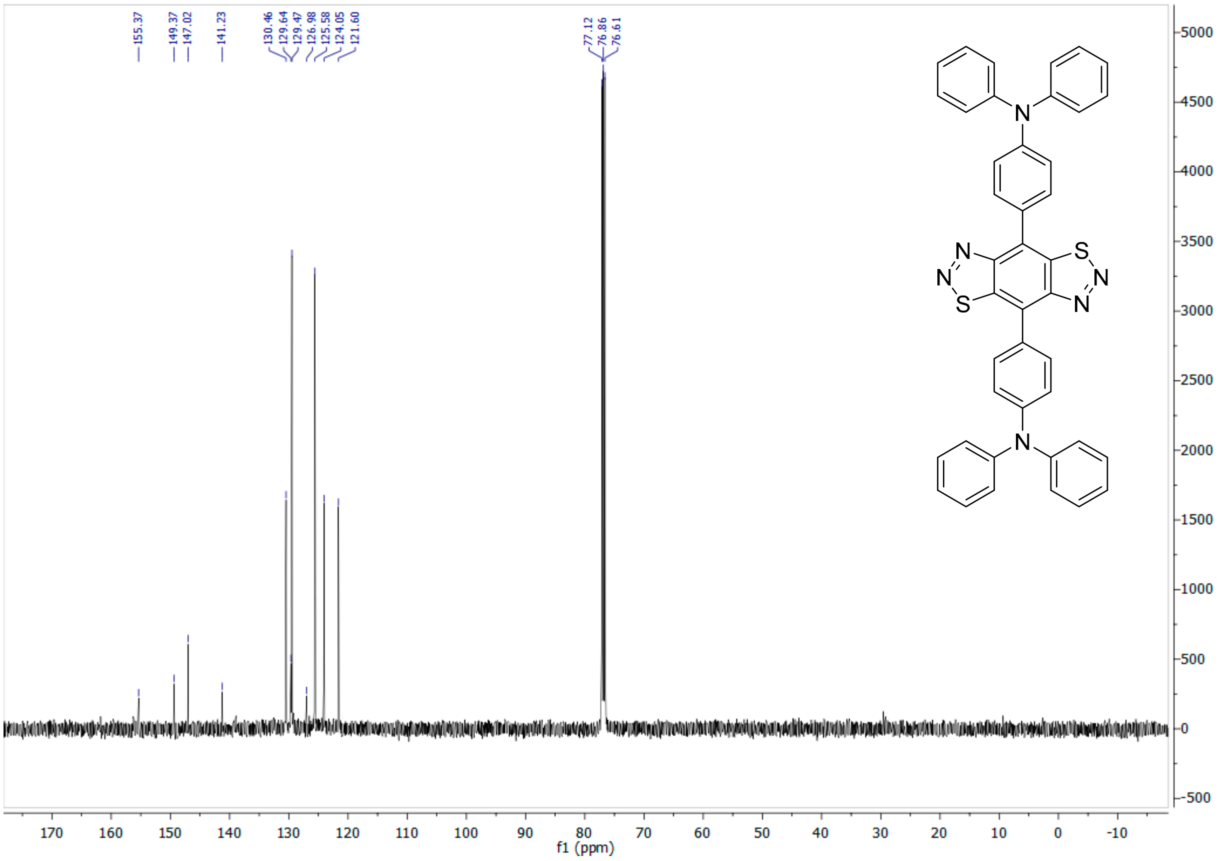
155.37
149.37
147.02
141.23
130.46
129.64
129.47
126.98
125.58
124.05
121.60
77.12
76.86
76.61
f1 (ppm)

Display Report

Analysis Info

Analysis Name	D:\Data\Kolotyrkina\2022\Chmovsh\0713009.d	Acquisition Date	13.07.2022 14:03:45
Method	tune_low.m	Operator	BDAL@DE
Sample Name	/SUSU Tim-539	Instrument / Ser#	maXis 43
Comment	C42H28N6S2 mH 681.1890clb added CH3CN		

Acquisition Parameter

Source Type	ESI	Ion Polarity	Positive	Set Nebulizer	0.4 Bar
Focus	Not active			Set Dry Heater	180 °C
Scan Begin	50 m/z	Set Capillary	4500 V	Set Dry Gas	4.0 l/min
Scan End	1500 m/z	Set End Plate Offset	-500 V	Set Divert Valve	Source

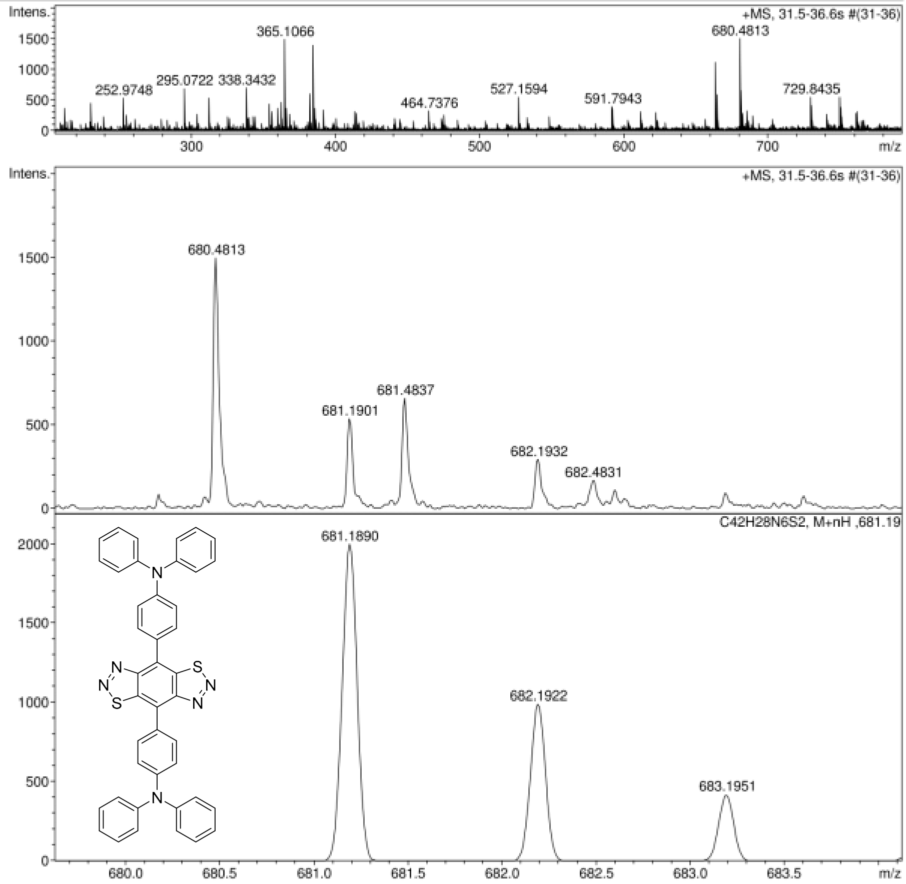

Quantum-chemical calculations

DFT calculations were performed with the Gaussian 16 Rev C.01.[1] DFT calculations were performed with the Gaussian 16 Rev C.01. M11 DFT functional with 6-31+g(d) basis set was used for all calculations. Calculations were performed in dichloromethane (PCM model). Data from X-ray diffraction experiments were used as starting points for geometry optimization. Cartesian coordinates are given in angstroms; absolute energies for all substances are given in hartrees. Analysis of vibrational frequencies was performed for the optimized structure. The compound was characterized by only real vibrational frequencies. Wavefunction stability, using *stable* keyword, was also checked for the molecule.

For calculations of optimized geometries, frequencies, thermodynamics, HOMO and LUMO energies following keywords were used:

# opt freq 6-31+g(d) scrf=(solvent=dichloromethane) nosymm m11 test

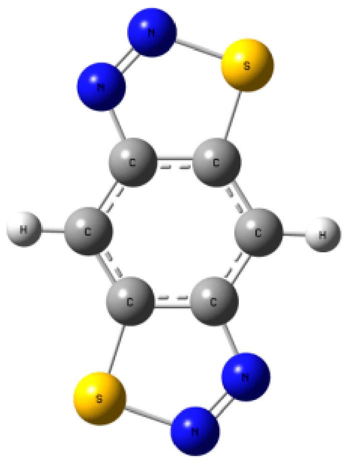

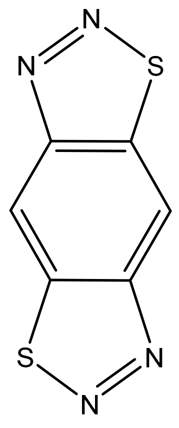

Charge 0; multiplicity 1

.

S	6.50031800	2.71554400	5.46659000
N	4.44929700	3.60408300	4.33387400
N	5.56231600	3.07917600	4.07791200
C	2.98324600	4.44144200	7.59676800
C	3.04398300	4.34416300	6.21269500
C	4.20732800	3.78281500	5.69345300
S	1.75621700	5.05904400	8.64216800
N	3.80724800	4.17051900	9.77488200
N	2.69423600	4.69543800	10.03084600
C	5.27329500	3.33315800	6.51198800
C	5.21255800	3.43043700	7.89606200
C	4.04921600	3.99178500	8.41530400
H	6.01272100	3.09520200	8.55422700
H	2.24382100	4.67939500	5.55453000

DFT M11 / 6-31+g(d) solvent dichloromethane, PCM model		
Total electronic energy=	-1244.877827	E_0
Sum of electronic and zero-point Energies=	-1244.797330	$E_0 + E_{ZPE}$
Sum of electronic and thermal Energies=	-1244.789137	$E_0 + E_{tot}$
Sum of electronic and thermal Enthalpies=	-1244.788193	$E_0 + H_{corr}$
Sum of electronic and thermal Free Energies=	-1244.831394	$E_0 + G_{corr}$
Zero-point correction *(unscaled)* =	0.080497	
Number of imaginary vibrational frequencies = 0		
E HOMO = -9.23 eV		
E LUMO = -1.04 eV		
E gap = 8.19 eV		

HOMO:

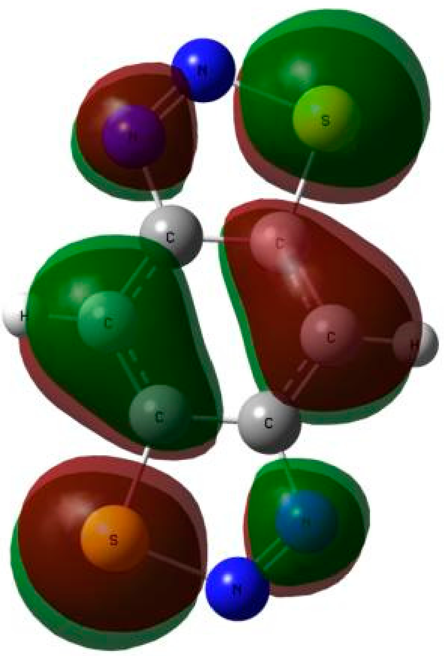

LUMO:

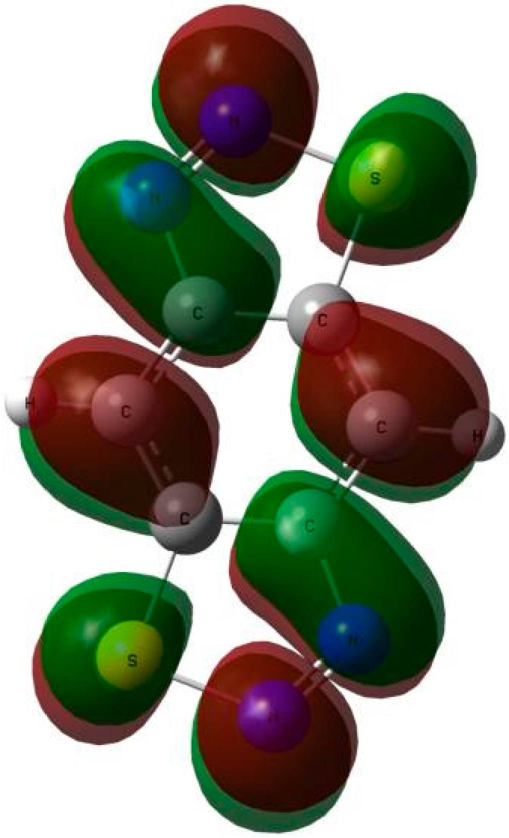
S
C
C
H
C
C
H
C
C
S
